# RAF1 deficiency causes a lethal syndrome that underscores RTK signaling during embryogenesis

**DOI:** 10.15252/emmm.202217078

**Published:** 2023-04-17

**Authors:** Samantha Wong, Yu Xuan Tan, Abigail Yi Ting Loh, Kiat Yi Tan, Hane Lee, Zainab Aziz, Stanley F Nelson, Engin Özkan, Hülya Kayserili, Nathalie Escande‐Beillard, Bruno Reversade

**Affiliations:** ^1^ Institute of Molecular and Cellular Biology A*STAR Singapore Singapore; ^2^ Experimental Drug Development Centre A*STAR Singapore Singapore; ^3^ Genome Institute of Singapore A*STAR Singapore Singapore; ^4^ Department of Pathology and Laboratory Medicine, David Geffen School of Medicine University of California Los Angeles Los Angeles CA USA; ^5^ Department of Human Genetics, David Geffen School of Medicine University of California Los Angeles Los Angeles CA USA; ^6^ 3billion, Inc Seoul South Korea; ^7^ Department of Biochemistry and Molecular Biology The University of Chicago Chicago IL USA; ^8^ Department of Medical Genetics Koç University, School of Medicine Istanbul Turkey; ^9^ Department of Physiology Cardiovascular Disease Translational Research Programme Yong Loo Lin School of Medicine National University of Singapore Singapore Singapore; ^10^ Smart‐Health Initiative, BESE KAUST Thuwal Saudi Arabia

**Keywords:** ASK1, ERK, RAF1, RASopathy, *Xenopus*, Development, Genetics, Gene Therapy & Genetic Disease

## Abstract

Somatic and germline gain‐of‐function point mutations in RAF, one of the first oncogenes to be discovered in humans, delineate a group of tumor‐prone syndromes known as the RASopathies. In this study, we document the first human phenotype resulting from the germline loss‐of‐function of the proto‐oncogene *RAF1* (a.k.a. *CRAF*). In a consanguineous family, we uncovered a homozygous p.Thr543Met variant segregating with a neonatal lethal syndrome with cutaneous, craniofacial, cardiac, and limb anomalies. Structure‐based prediction and functional tests using human knock‐in cells showed that threonine 543 is essential to: (i) ensure RAF1's stability and phosphorylation, (ii) maintain its kinase activity toward substrates of the MAPK pathway, and (iii) protect from stress‐induced apoptosis mediated by ASK1. In *Xenopus* embryos, mutant RAF1^T543M^ failed to phenocopy the effects of normal and overactive FGF/MAPK signaling, confirming its hypomorphic activity. Collectively, our data disclose the genetic and molecular etiology of a novel lethal syndrome with progeroid features, highlighting the importance of RTK signaling for human development and homeostasis.

## Graphical abstract

A novel recessive variant in RAF1 was identified as a possible cause of a hitherto unknown lethal neonatal syndrome with progeroid features. This private p.Thr543Met mutation abolished downstream ERK pathway signaling while increasing susceptibility to apoptosis via ASK1 de‐repression.

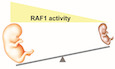

**The paper explained**

**Problem**

We have studied a severe syndrome causing early death in two siblings. The clinical presentation comprises skin, face, heart, and limbs malformation with progeroid features. This bears some resemblance to Acrocardiofacial syndrome (ACFS), the genetic etiology of which remains unknown.

**Results**

Exome sequencing revealed that the disease was segregating with a private germline homozygous p.T453M variant in *RAF1*. Traditionally studied in the context of cancer, RAF1 is a protein kinase responsible for driving cellular proliferation. Functional investigations relying on *in vitro* and *in vivo* tests demonstrated that tThr543 is necessary for RAF1's protein stability, to transduce signaling to the MAPK pathway and to respond to stress‐induced apoptosis. Normally, RAF1 typically blocks cell death by sequestering the death‐promoting protein ASK1. However, mutant RAF1^T543M^ appears to lose this property, thereby allowing ASK1 to induce cell death in the face of cellular insults.

**Impact**

Our results suggest that loss‐of‐function mutations in RAF1 may underlie some characteristics of ACFS. Given the crucial role of RAF1 in mediating cell growth via mitogen‐induced signaling, it is fitting that a loss‐of‐function mutation would cause severe developmental defects leading to early demise. This is in contrast to a class of syndromes known as RASopathies that are driven by gain‐of‐function mutations in genes of this same pathway. RASopathies tend to lead to overgrowth and cancer‐predisposition, a set of phenotypes that is opposite to what is witnessed in these affected siblings. Our collective findings illustrate the physiological importance of MAPK signaling during embryogenesis and, for the first time, reveal the consequence of a whole body loss‐of‐function variant in RAF1 in humans.

## Introduction

Rapidly Accelerated Fibrosarcoma 1 (RAF1, MIM164760) is one of three mammalian RAF isozymes that delineate the conserved family of receptor tyrosine kinase (RTK) effectors in metazoans (Leicht *et al*, 2007). This 70 kDa serine–threonine kinase is part of the Ras/Mitogen‐activated kinase (MAPK) pathway. Binding of extracellular growth factors/mitogens to cell‐surface RTKs (such as Fibroblast Growth Factor [Receptor] FGF/FGFR) activates Rat sarcoma protein (RAS) and subsequently RAF (ARAF [MIM311010], BRAF [MIM164757], and/or RAF1), triggering a phosphorylation cascade through mitogen‐activated protein kinase kinase (MEK1/MEK2) and the ultimate effectors extracellular signal‐regulated protein kinase (ERK1/ERK2; Rauen, 2013). ERK1/ERK2 exert their function on numerous downstream nuclear and cytosolic targets culminating in increased cell proliferation. Unsurprisingly, the earliest studies documented the somatic dysregulation of RAF1 as a primary driver of tumorigenesis (Leicht *et al*, 2007). However, for the same reasons, more recent RAF research has revealed the regulatory role of RAF in physiological growth and most importantly in the context of this work, human embryogenesis (Baccarini, 2005; Rauen, 2013). Many of these new insights arose from clinical observations of how germline mutations in regulators or components of the Ras/MAPK pathway exhibit a distinct but overlapping phenotype, owing to their common underlying mechanism (Rauen, 2013).

This class of congenital developmental disorders came to be referred to as “RASopathies.” It encompasses syndromes such as Noonan syndrome (caused by mutations in *RAF1* [MIM611553], *PTPN11* [MIM151110], *SOS1* [MIM610733], *KRAS* [MIM609942] or *SHOC2* [MIM607721]), Costello Syndrome (*HRAS*, MIM218040), cardiofaciocutaneous syndrome (*BRAF*, *MAP2K1*/*2*, *KRAS*), neurofibromatosis type I (*NF1*, MIM162200), capillary malformation‐arteriovenous malformation (*RASA1*, MIM139150), Noonan syndrome 13 (MAPK1, MIM176948), and Noonan syndrome 14 (SPRED2, MIM619745; Motta *et al*, 2021). Afflicted individuals often present with craniofacial dysmorphisms, ocular, musculoskeletal and cutaneous abnormalities, hypotonia, neurocognitive impairment, cardiac malformations, and increased cancer risk (Rauen, 2013; Aoki *et al*, 2016). While these syndromes tend to be inherited in an autosomal dominant manner, many of them arise *de novo* in the parental germline or somatically in the patient and exhibit variable penetrance and severity (Tajan *et al*, 2018).

Of the approximately 22 genes implicated in RASopathies, those of the RAF family remain one of the best‐studied owing to their cancer‐associated roles. Genetic inactivations in mice have confirmed their nonredundant roles during development. *Araf* ^−/−^ pups die postnatally with distinct neurological and gastrointestinal abnormalities while conditional *Braf* ^−/−^ mice have defective oligodendrocyte development and constitutive *Braf* ^−/−^ mice are embryonic lethal due to placental vascular defects (Pritchard *et al*, 1996; Galabova‐Kovacs *et al*, 2006, 2008)*. Raf1* ^−/−^ mice die by embryonic day e12.5, as a consequence of increased placental and liver apoptosis, while cardiac‐specific *Raf1* ^−/−^ mice exhibit extensive cardiac abnormalities (Wojnowski *et al*, 1997, 1998; Yamaguchi *et al*, 2004). RAF1 is intricately controlled by numerous phosphorylation sites (Dumaz & Marais, 2005). In quiescent cells, phosphorylated Ser621 and Ser259 recruit 14‐3‐3, which enhances the binding of the inhibitory N‐terminal conserved region (CR1) domain of RAF1 to the catalytic C‐terminal kinase domain to dampen its activity (Dhillon *et al*, 2002; Dougherty *et al*, 2005; Dumaz & Marais, 2005). Upon mitogenic stimulation, key activating residues such as Ser338, Tyr341, Thr491, and Ser494 are phosphorylated (Diaz *et al*, 1997; Chong *et al*, 2001). Interestingly, residues such as Ser29, Ser43, Ser289, Ser296, Ser301, and Ser642 are also phosphorylated to boost RAF1 to a hyperphosphorylated state, which then renders RAF1 refractory to further stimulation and promote its downregulation instead (Dougherty *et al*, 2005 ). Concurrently, inhibitory residues such as Ser259 are dephosphorylated by a SHOC2 complex to support RAF1 activation (Dhillon *et al*, 2002; Jones *et al* 2019). Given the intricacy of RAF1 regulation at multiple residues, missense variants have been associated with detrimental phenotypes, such as cardiac defects. p.S257L, p.P261T, p.L613V, and p.T491I are implicated in hypertrophic cardiomyopathy (HCM) (Chen *et al*, 2001), while p.L603P, p.H626A, p.T641M, and p.T310A are associated with dilated cardiomyopathy (DCM) (Dhandapany *et al*, 2014; Jaffré *et al*, 2019). Consistent with their dominant mode of inheritance, the majority of these detrimental RAF1 germline mutations exert gain‐of‐function activity vis‐a‐vis MAPK signaling. To date, no genetic data have been documented about the physiological requirement of wild‐type RAF1 during human development.

Here, we describe a previously unreported p.T543M variant in RAF1 segregating with a neonatal lethal phenotype in a consanguineous Turkish family. Afflicted neonates presented with split hand/foot malformations, craniofacial, cutaneous, and cardiac abnormalities, which are matching the commonly affected organs seen in RASopathies. However, instead of the characteristic autosomal dominant inheritance, this phenotype was inherited in an autosomal recessive manner. Our collective *in vitro* and *in vivo* functional tests demonstrate that p.T543M behaves as a hypomorphic loss‐of‐function RAF1 mutant with impaired kinase activity, reduced protein half‐life, and increased cellular susceptibility to stress‐induced apoptosis.

## results

### Clinical phenotype and genetic analysis

**Figure 1:**
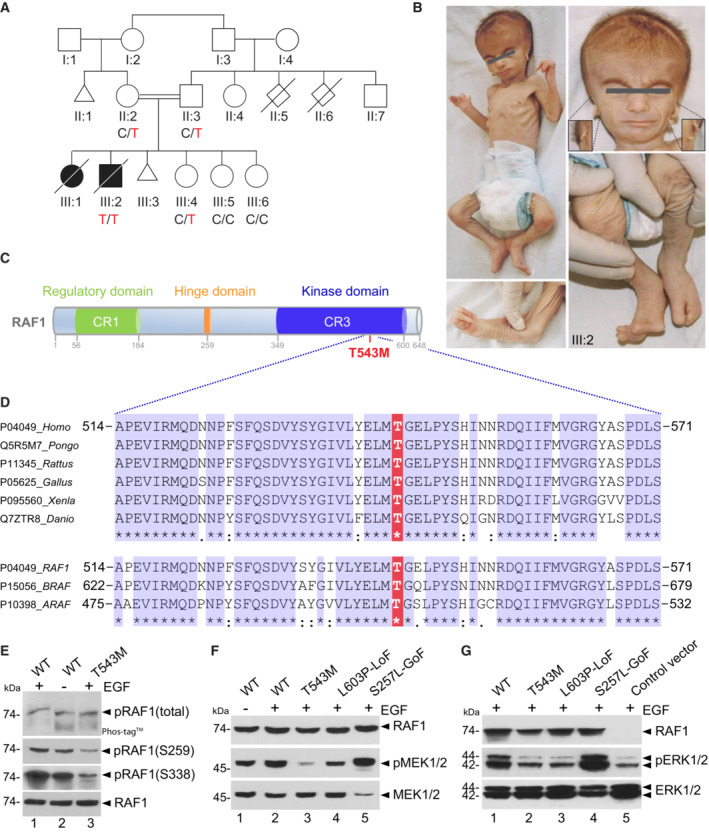
RAF1 germline homozygous missense mutation causes a neonatal lethal syndrome with progeroid features APedigree of the Turkish consanguineous family reporting the neonatal progeroid syndrome. Square, male; circle, female; black shading, affected individuals; small triangle, aborted; double lines, consanguineous marriages; diagonal line, deceased.BPictures of affected proband (III:2) at 50 days showing general progeroid features. Note subcutaneous lipoatrophy, severe hypotonia, distinctive craniofacial features with absence of external ears (insets), cleft hands and feet with oligodactyly.CSchematic of the RAF1 protein structure showing the three conserved regions (CRs). p.T543M is located in the catalytic kinase domain (CR3).DMultiple sequence alignment showing conservation of T543 across species and among RAF family members.ERAF1^WT^ or RAF1^T543M^ were transiently overexpressed in HEK293T cells for 48 h, followed by 16 h of serum starvation and EGF stimulation for 15 min. Phos‐tag™ gel showing global levels of RAF1 phosphorylation and corresponding Western blot showing phosphorylation levels of Ser259 and Ser338 residues and total RAF1.F, GAll the RAF1 constructs were transiently overexpressed in HEK293T cells as described in (E). Western blotting showing (F) phosphorylated MEK1/2, total MEK1/2 and total RAF1 and (G) phosphorylated ERK1/2, total ERK1/2 and total RAF1. Source data are available online for this figure.

Here, we report two affected siblings born to healthy, consanguineous first‐cousin Turkish parents (Fig 1A). Both siblings, one male and one female, exhibited similar craniofacial, cardiac, and limb abnormalities suggestive of autosomal recessive inheritance. The first affected girl (III:1) had died on the seventh day of life due to necrotizing enterocolitis progressing to septicemia, and no medical autopsy was available. The male proband was born via normal spontaneous delivery with a birth weight of 2,670 g and length of 47.2 cm. He was hospitalized after birth for a week due to poor adaptation. He had craniofacial anomalies, complex cardiac anomalies, and cleft hands and feet. He was seen at genetics outpatient clinics at 50 days of age and had a weight of 1950 g, length of 51 cm, and an occipital frontal circumference (OFC) of 33 cm. His general condition was very poor, and found to be hypotonic with no newborn reflexes. He had dry, lax, translucent skin with no subcutaneous fat. Craniofacial anomalies included the following: overriding cranial sutures with open frontal fontanelle of 2 × 2 cm, a triangular face, prominent nasal root with hypoplastic alae nasi, narrow palpebral fissures, cleft palate, very small external ears with atresia of the right external meatus and a preauricular tag. In terms of extremities, the neonate had split hands with missing second and third fingers, complete cutaneous syndactyly of fourth‐to‐fifth fingers with fused nails on right and separate nail structures on left. The palmar lines were aberrant in both hands. There were three toes in each foot, second and third missing with cleft extending till distal metatarsal joints, total cutaneous syndactyly of fourth‐to‐fifth toes and hypoplastic distal phalanx of hallux with rudimentary nail on one side and missing on the other. He had male genitalia and left cryptorchidism (Fig 1B). He had cardiac systolic murmur of 3/6 and an echocardiogram revealed severe aortic stenosis, dysplastic aortic valve, muscular ventricular septal defect (VSD), patent doctus arteriosus (PDA), patent foramen ovale (PFO), and pulmonary hypertension and left ventricular dysfunction. Thin partial corpus callosum agenesis leading to wide occipital horns, colpocephaly, was detected during cerebral ultrasonography. He had bilateral grade 3 hydroureteronephrosis and hypoplastic kidneys (R: 31 × 17 × 19 and L: 32 × 19 × 16 mm) with no corticomedullary differentiation. Death occurred at 50 days of age due to cardiopulmonary arrest. No autopsy was performed. The aforementioned clinical features, albeit more severe, were reminiscent of Acro‐Cardio‐Facial Syndrome (ACFS, MIM600460). Acro‐Cardio‐Facial Syndrome is a very rare congenital malformation syndrome with no identified genetic etiology (Richieri‐Costa, 1987; Giannotti *et al*, 1995; Guion‐Almeida *et al*, 2000; Mingarelli *et al*, 2005; Sivasli *et al*, 2007; Kariminejad *et al*, 2008; Tanpaiboon *et al*, 2009; Toschi *et al*, 2012; Hudson *et al*, 2014). Table 1 summarizes and compares the 20 historical ACFS cases with the phenotypes of two affected siblings reported in this study.

**Table 1:** Clinical table comparing all the published ACFS cases with the two patients from this study.

	Patient ID	1	2	3	4	5	6	7	8	9	10	11	Present cases	
	Reference	Richeri‐Costa & Orquizas (1987)	Giannotti *et al* (1995)		Guion‐Almeida *et al* (2000)	Mingarelli *et al* (2005)	Sivasli *et al*, 2007	Kariminejad *et al* (2008)		Tanpaiboon *et al* (2009)	Toschi *et al* (2012)	Hudson *et al* (2014)	This study	
	PubMed ID	N.A.	7897634		11045583	15937946	17710878	18627040		19606477	22740423	24715610	N.A.	
	Gender	M	M	F	M	M	F	M	F	M	M	F	F	M
	Ethnicity	Brazilian	Brazilian		Brazilian	Italian	Turkish	Iranian		Thai	Italian	N.A.	Turkish	
	Consanguinity	Yes	No		No	No	Yes	Yes		No	No	No	Yes	
	Survival	Alive at 4 years	1 month	6 hours	4 months	1 month	50 days	Alive at 25 years	4 days	14 years	Alive at 7 months	Alive at 2 months	7 days	50 days
**Ectrodactyly**	**HPO terms**													
Cleft hand	HP:0001171	+	+	+	+	+	+	+	+	+	+	+	+	+
Cleft foot	HP:0001839	+	−	−	−	+	−	−	+	−	−	−	+	+
Syndactyly of toes	HP:0001770	N.D.	−	+	−	+	−	−	−	−	N.D.	N.D.	+	+
**Facial anomalies**														
Dolichocephalic skull	HP:0000268	N.D.	+	N.D.	N.D.	N.D.	N.D.	N.D.	N.D.	N.D.	N.D.	N.D.	+	+
High forehead	HP:0000348	−	+	N.D.	+	+	N.D.	+	+	−	+	−	+	+
Flat bridge / broad nasal ridge	HP:0000431	+	+	N.D.	+	+	N.D.	N.D.	N.D.	+	+	N.D.	+/−	+/−
Upslanting palpebral fissures	HP:0000582	N.D.	−	N.D.	−	N.D.	N.D.	−	−	+	−	−	+/−	+/−
Blepharophimosis/ptosis	HP:0000581	−	+	N.D.	+	N.D.	N.D.	+	+	−	N.D.	N.D.	+	+
Prominent eyes and ears	HP:HP:0000520 HP:HP:0000411	−	+	N.D.	+	+	N.D.	N.D.	N.D.	−/+	−	N.D.	−	−
Hypertelorism	HP:0000316	−	−	N.D.	+	+	N.D.	+	+	+	−	+	−	−
Flat/long philtrum	HP:0000319	N.D.	+	N.D.	N.A.	+	N.D.	N.D.	N.D.	N.A.	+	N.D.	+/+	+/+
Cleft lip/palate	HP:0000202	+/+	−	−/+	+/+	−	+	−	−	+/+	−	N.D.	+/−	+/−
Micrognathia	HP:0000347	N.D.	+	N.D.	−	N.D.	N.D.	N.D.	N.D.	+	+	+	+	+
Dysmorphic/low set ears	HP:0000369	+	+	N.D.	+	+	+	+	+	+	+	+	+	+
Preauricular abnormalities	HP:0000384	N.D.	−	N.D.	−	N.D.	N.D.	N.D.	N.D.	−	+	N.D.	+	+
**Cardiac defects**														
VSD	HP:0001629	+	+	−	−	+	+	−	N.D.	+	+	−	N.D.	+
ASD	HP:0001631	−	+	−	−	−	+	−	N.D.	−	−	+	N.D.	−
Mitral atresia	HP:0011560	−	+	−	−	−	−	−	N.D.	−	−	−	N.D.	−
Hypoplastic left ventricle	HP:0001711	−	+	−	−	−	−	−	N.D.	−	−	−	N.D.	+
Aortic coarctation	HP:0001680	−	+	−	+	−	−	−	N.D.	−	−	−	N.D.	−
Patent Ductus Arteriosus	HP:0001643	−	+	−	+	−	+	−	N.D.	−	−	−	N.D.	+
Truncus arteriosus	HP:0001660	−	−	−	−	+	−	−	N.D.	−	+	−	N.D.	−
Dysplastic aortic valve / Stenosis	HP:0005176/HP:0001650	−	−	−	−	+	−	−	N.D.	−	−	−	N.D.	+
Patent foramen ovale	HP:0001655	−	−	−	−	−	−	−	N.D.	−	−	−	N.D.	+/+
Tetralogy of Fallot	HP:0001636	−	−	−	−	−	−	−	N.D.	+	−	−	N.D.	−
**Genital anomalies**														
Micropenis	HP:0000054	+	+	N.A.	−	−	N.A.	+	N.A.	+	−	N.A.	N.A.	+
Cryptorchidism	HP:0000028	+	+	N.A.	−	+	N.A.	+	N.A.	−	−	N.A.	N.A.	+
Hypospadias	HP:0000047	+	+	N.A.	−	−	N.A.	+	N.A.	−	−	N.A.	N.A.	−
**Neurological anomalies**														
Hypertonia/Hypotonia	HP:0001276/HP:0001252	+	−	N.A.	N.D.	+	N.D.	−	N.D.	−	+	+	−/+	−/+
Developmental Delay	HP:0012758	+	N.A.	N.A.	+	N.A.	N.D.	−	N.A.	+	+	+	N.A.	+
Intellectual disability	HP:0001249	+	N.A.	N.A.	N.A.	N.A.	N.A.	−	N.A.	+	+	N.A.	N.A.	N.A.
Cortical brain atrophy	HP:0002120	+	−	N.D.	+	−	N.D.	−	N.D.	−	−	N.D.	N.D.	+
Neuroepithelial cyst	HP:0030063	−	−	N.D.	−	−	+	−	N.D.	−	−	N.D.	N.D.	N.D.
Subependymal cysts	HP:0002416	−	−	N.D.	−	−	N.D.	−	N.D.	−	+	N.D.	N.D.	N.D.
Colpocephaly	HP:0030048	−	−	N.D.	−	−	N.D.	−	N.D.	−	−	N.D.	N.D.	+
Underdeveloped corpus callosum	HP:0002079	−	−	N.D.	−	−	N.D.	−	N.D.	−	+	N.D.	+	+
**Growth abnormalities**														
Low birth weight (< 3^rd^ centile)	HP:0001518	−	+	+	−	−	−	+	+	+	−	−	−	−
Postnatal growth retardation	HP:0001510	+	+	N.A.	+	N.D.	N.D.	+	N.A.	+	−	N.D.	N.A.	+
**Others**														
			Oligohydramnios; mesenterium communis; rudimentry atretic vagina in scrotum	Oligohydramnios	Polyhydramnios; scarring cornea clouding; seizures; gastroesophageal reflux; large mega cisterna magna		Hydrocephaly; polydactyly of the feet	Pre‐eclampsia; obesity	Imperforate anus	Absent left pulmonary artery; mild scoliosis (hemivertebrae T4, 5, 8, 9, 11); hyperthyroidism	Large lateral ventricles, simplified cortex gyration, subcortical and periventricular white matter hyperintensities; pulmonary atresia; 12.5 Mb interstitial deletion at 6q21‐22.31	Strabismus; seizure activity on EEG; 11.15 Mb interstitial deletion at 6q21‐22.3		Lack of subcutaneous fat; lax, translucent skin; hydroureteronephrosis; kidney hypoplasia

ASD, atrial septal defect; F, female; M, male; N.A., not applicable/available; N.D., not determined; VSD, ventricular septal defect.

**Figure EV1:**
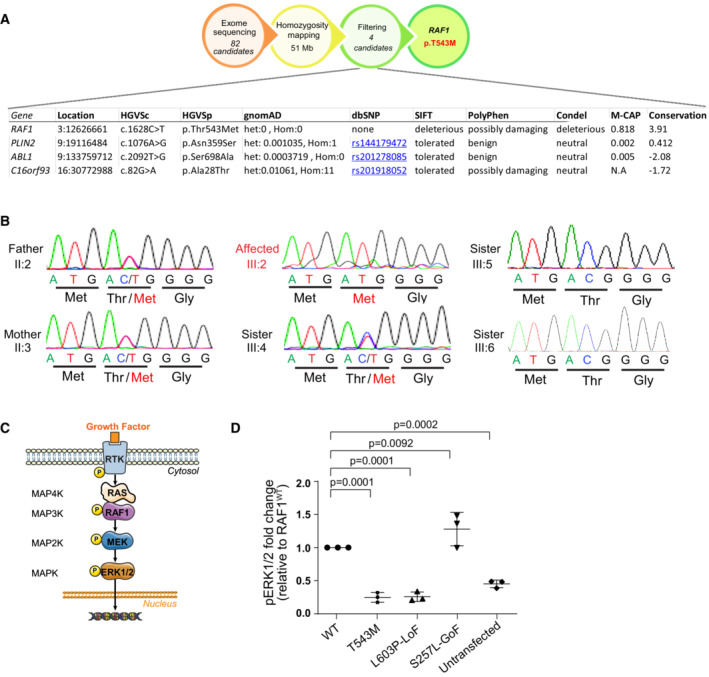
RAF1^T543M^ allele behaves as a loss‐of‐function mutation *in vitro* AGenetic strategy. Combination of exome sequencing and homozygosity mapping. resulted in four candidate genes. Germline homozygous variants in *PLIN2*, *ABL1*, and *C16orf93* were filtered out due to their low phylogenetic conservation and low predicted deleterious potential.BSanger sequencing chromatogram showing variant c.1628C>T (p.T543M) segregating with disease: homozygous in the proband (III:2), heterozygous in the parents (II:2 and II:3) and one sister (III:4) and absent in two other siblings (III:3 and III:6).CSchematic representation of ERK1/2 MAP Kinase pathway.DQuantification of phosphorylated ERK1/2, normalized to WT RAF1 in three independent Western blots (Error bars indicate mean ± SEM. Ordinary one‐way ANOVA).

Homozygosity mapping followed by exome sequencing revealed a unique germline homozygous missense mutation in *RAF1* (Chr:3 c.1628C>T, p.T543M) previously unrecorded in ExAC, UK10K, GnomAD, or within our in‐house exome databases (Fig EV1A). Sanger sequencing confirmed that this novel *RAF1* mutation segregated with the disease in all available family members (Fig EV1B). Thr543 is highly conserved across vertebrate RAF1 orthologues (PhyloP = 6.2, PhastCons = 1) and RAF paralogues (Figs 1D and EV1A). It is situated in the kinase domain of RAF1 (Fig 1C), and p.T543M is predicted deleterious in all databases (SIFT = 0, PolyPhen = 0.999, MCAP = 0.818, PROVEAN = ‐4.12 and MutationTaster). Given RAF1's role in transducing the growth‐promoting activities of numerous RTKs during embryogenesis, we hypothesized that this homozygous germline RAF1^T543M^ allele might be the cause of this hitherto unknown neonatal lethal syndrome.

### The RAF1^T543M^ allele mimics a loss‐of‐function mutation in cultured cells

To investigate the pathogenicity of p.T543M, we assessed RAF1 and downstream MAPK/ERK activity in cultured cells (Fig EV1C). RAF1 activity is controlled by multiple phosphorylation sites (Dumaz & Marais, 2005). A Phos‐tag® assay revealed that mutant RAF1^T543M^ remained globally hypophosphorylated (i.e., reduced electrophoretic mobility) compared with wild‐type RAF1 even if stimulated by exogenous epidermal growth factor (EGF) (Fig 1E). Consistent with this, phospho‐specific RAF1 antibodies against Ser259 and Ser338 showed a significantly lower signal for RAF1^T543M^ relative to RAF1^WT^, suggesting that it was refractory to EGF‐triggered transduction (Fig 1E). We next examined the MAPK/ERK pathway using three variants of RAF1 against which the RAF1^T543M^ can be evaluated: RAF1^WT^, a gain‐of‐function (GoF) allele RAF1^S257L^ (positive control), the most common activating mutation associated with HCM in Noonan syndrome, and the heterozygous‐acting loss‐of‐function (LoF) RAF1^L603P^ mutation associated with childhood‐onset dilated cardiomyopathy (negative control) (Dhandapany *et al*, 2014; Jaffré *et al*, 2019). Western blot analyses showed that upon EGF stimulation, RAF1^T543M^ like the LoF construct RAF1^L603P^, failed to induce downstream MEK1/2 and ERK1/2 phosphorylation. As expected, RAF1^WT^, and even more so RAF1^S257L^, induced significantly more endogenous EGF‐triggered MEK and ERK phosphorylation downstream of RAS/RAF1 (Figs 1F and G and EV1D). We conclude from these *in vitro* experiments that the p.T543M mutation is a loss‐of‐function mutation that dampens RAF1‐mediated MEK/ERK signaling.

### The RAF1^T543M^ allele behaves as a loss‐of‐function mutation *in vivo*

**Figure 2:**
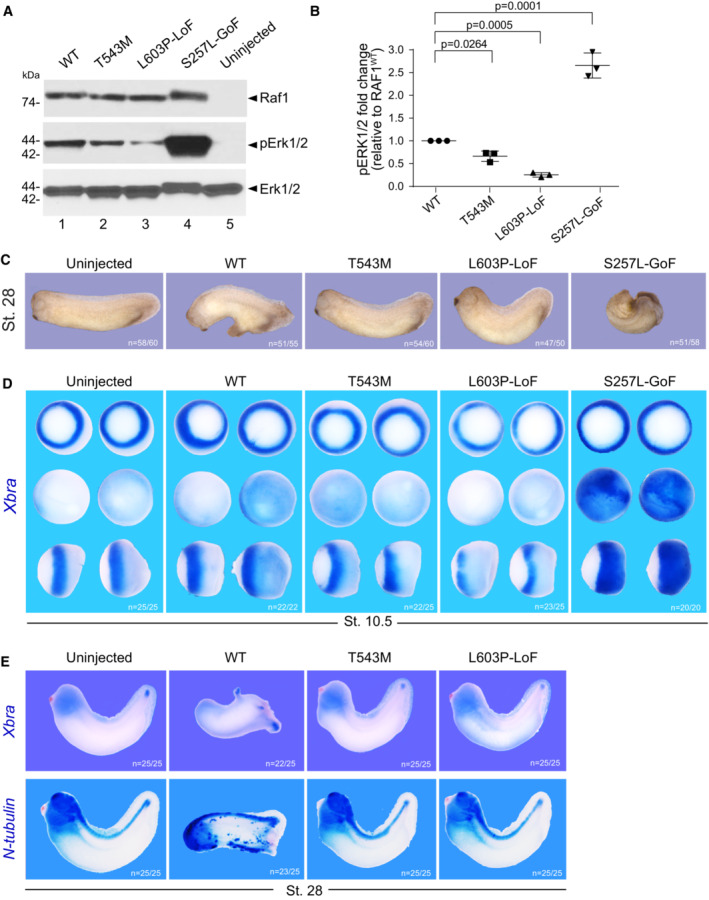
RAF1^T543M^ allele behaves as a loss‐of‐function mutation *in vivo* AWestern blotting showing phosphorylated Erk1/2, total Erk1/2 and total Raf1 from *Xenopus laevis* embryos harvested at stage 28.BQuantification of phosphorylated Erk1/2, normalized to WT Raf1 in three independent Western blots (Error bars indicate mean ± SEM. Ordinary one‐way ANOVA).CRepresentative images illustrating the effect of WT or mutants *RAF1* mRNA injection into *Xenopus* embryos, at stage 28.DWISH performed on stage 10.5 *Xenopus* embryos injected with WT or mutants *RAF1* mRNA. *Xbra* (blue staining) marking mesoderm induction. Vegetal (top), animal (middle) and lateral (bottom) views.EWISH performed on stage 28 *Xenopus* embryos injected with WT or mutants *RAF1* mRNA. Blue staining reveals *Xbra* or *N‐tubulin* markers depicting ectopic mesoderm induction, neural differentiation and brain posterization. Source data are available online for this figure.

We next assessed the signaling potential of these same RAF1 constructs in developing *Xenopus laevis* embryos, which provide a powerful *in vivo* test tube model for FGF/MAPK signaling. Endogenous FGF/FGFR signaling is required for proper mesoderm and neural induction. Forced expression of dominant‐negative or activating mutants of different components of this pathway yield tractable readouts (MacNicol *et al*, 1993; Umbhauer *et al*, 1995; Pera *et al*, 2001; Delaune *et al*, 2005). Upon overexpression in 2‐ to 4‐cell stage embryos and Western blots on extracts of stage 10 embryos showed that, like in HEK293T cells, the RAF1^T543M^ and the LoF RAF1^L603P^ cause significant reduced downstream ERK1/2 phosphorylation relative to the WT and GoF constructs (Fig 2A and B). Phenotypically, increased RAF1^WT^ activity induced ectopic mesoderm differentiation. This effect was significantly augmented by GoF RAF1^S257L^ (Fig 2C). This was readily quantified by increased *Xbra* expression in the circumferential layer of mesodermal cells at stage 10.5 and later by the development of supernumerary tails at stage 28 (Fig 2D and E). Like in HEK293T cells, RAF1^T543M^ induced minimal ectopic mesoderm which was comparable to that of LoF RAF1^L603P^ or uninjected controls (Fig 2D and E). Similarly, N‐tubulin staining of primary neuronal differentiation revealed that only overexpressed RAF1^wt^, but not RAF1^L603P^ or RAF1^T543M^, could induce ectopic neuronal differentiation, a hallmark of FGF‐mediated posterization of the CNS (Fig 2E). These results lend credence to the notion that the p.T543M allele is a hypomorphic mutation that cannot signal to its full extent. Using gastrulating *Xenopus* embryos, we provide *in vivo* evidence that the missense p.T543M is a *bona fide* loss‐of‐function allele of RAF1 that cannot sustain FGF signaling.

### Structure‐based predictions and tests validate the pathogenicity of p.T543M

**Figure 3:**
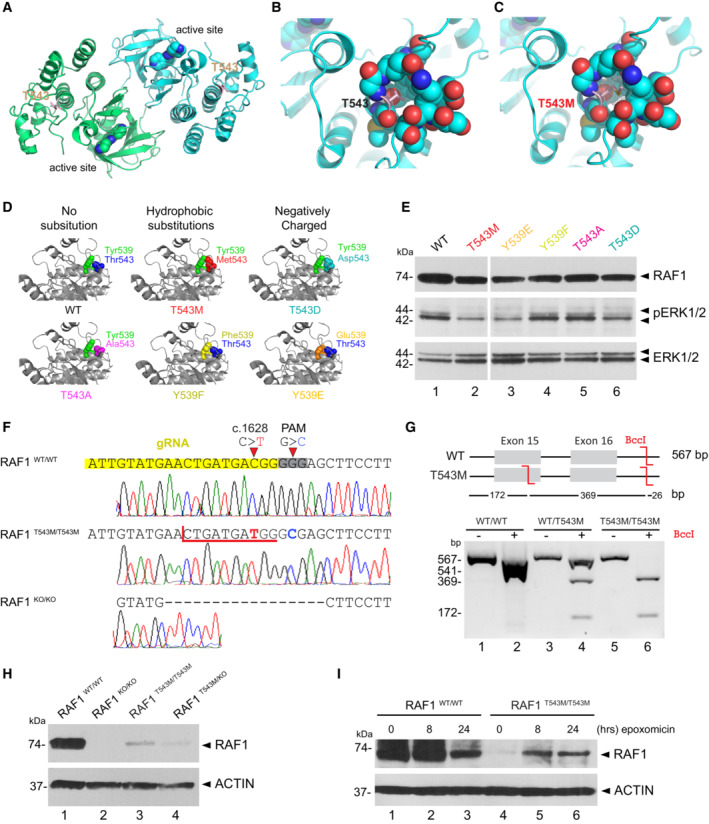
RAF1^T543M/T543M^ knock‐in cells reveal RAF1's compromised protein half‐life ARAF1 homodimer (PDB:3OMV) showing the location of Thr543 (drawn as ball‐and‐stick) on helical subdomain. Active site is occupied by an inhibitor drawn as spheres.BPositioning of Thr543 in the pocket.CMet543 in the pocket. The Met543 side chain clashes despite being modeled in the least sterically hindered rotamer.DPredicted side chain interaction of Thr543 and Tyr539 variants.EWestern blot downstream signaling potential of structural mutants that induce steric (p.T543M) or electrostatic (p.Y539E or p.T543D) interference.FRAF1^T543M^ and RAF1^KO/KO^ HEK293T cells were generated by CRISPR‐Cas9. Top—bottom: Sanger‐sequencing of RAF1 in WT HEK293T cells; a RAF1^T543M^ clone showing the desired c.1628C>T (p.T543M) mutation and a synonymous PAM‐disrupting c.1632G>C; a RAF1^KO/KO^ clone with a homozygous 17 bp deletion.GConfirmation of a clonal RAF1^T543M^ line by restriction‐digest of RAF1 exons 15–16 from gDNA of the genotypes shown with BccI. c.1628C>T introduces a BccI restriction site. The RAF1^WT/T543M^ heterozygote genotype was obtained from the patient's father.HWestern Blot for endogenous RAF1 in HEK293T cell lines of genotypes indicated, at steady state.IWestern‐blot showing that 8 and 24 h of epoxomicin treatment is sufficient to partially rescue RAF1 in RAF1^T543M/T543M^ cells. ACTIN serves as loading control. Source data are available online for this figure.

**Figure EV2:**
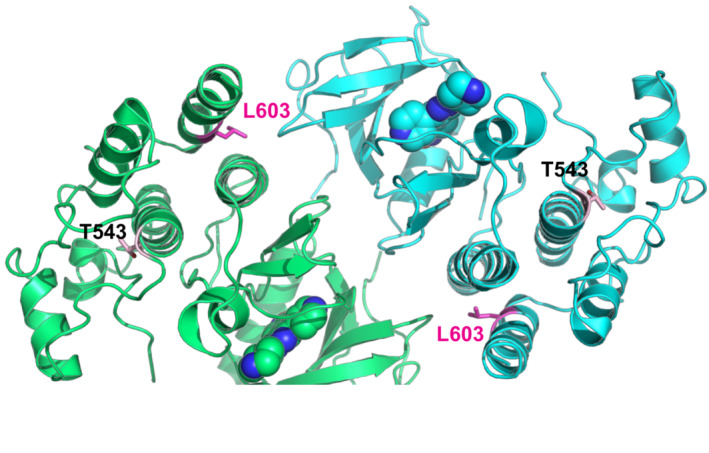
Loss‐of‐function mutation p.L603P impairs RAF1 kinase activity Position of Leu603 and Thr543 in the RAF1 homodimer (PDB:3OMV). The p.L603P mutation causes an impairment of kinase activity, likely through destabilizing the αH helix and the helical bundle made by the αC and αH helices, and may also affect the dimer stability. Thr543 is located in the αE helix.

*In silico* structural analysis showed that Thr543 is located within the helical subdomain of RAF1 ~ 22 Å away from the active site. Unlike Thr341 or Thr491, Thr543 is not known as a kinase‐activating phosphorylation site (Fig 3A). Instead, Thr543 is a polar residue mostly buried within the protein, with the beta‐oxygen of its side‐chain forming a hydrogen bond with main‐chain Glu545 (Fig 3B). A missense mutation to a methionine abolishes the hydrogen bond and causes steric clashes within the pocket (Fig 3C). This can have knock‐on effects toward either the active site or may deform a yet‐unknown interaction site (Fig 3C). Interestingly, the loss‐of‐function mutation p.L603P also affects a nearby helix located equidistant from the active site (Fig EV2B) (Dhandapany *et al*, 2014).

To investigate the predicted importance of Thr543 on local RAF1 structure, we substituted Thr543 and Tyr539, which closely packs onto Thr543 (Fig 3D; PDB: 3OMV; Hatzivassiliou *et al*, 2010), with a range of other amino acids *in silico*. Of these, p.T543A and p.Y539F (substitutions with shortened side chains) still fit within the pocket and retained local structure and RAF1 activity (Fig 3D). Conversely, we also modeled substitutions that likely disrupted RAF1 structure and kinase activity via steric (p.T543M) means, electrostatic means (p.Y539E), or both (p.T543D) (Fig 3D). These predictions were verified experimentally in HEK293T cells. Substitutions that shortened the side chains (p.Y539F and p.T543A) preserved RAF1 kinase activity (Fig 3E), as measured by downstream phosphorylation of ERK1/2, whereas substitutions that likely cause steric (p.T543M) or electrostatic (p.Y539E and p.T543D) disruptions indeed led to a loss of kinase activity (Fig 3E). These results suggest p.T543M abrogates RAF1 kinase activity potentially through disruption of a crucial local and possibly global structure within its kinase domain. As p.T543A and p.Y539F cannot be phosphorylated, these results also argue against the involvement of a possible phosphorylation at these sites to control RAF1 function.

### Knock‐in cells reveal a reduced half‐life for endogenous RAF1^T543M^

While we demonstrated that overexpressed RAF1^T543M^ is kinase‐deficient, several reports have found that the kinase activity is required for RAF1's inherent stability by blocking its proteasomal degradation (Noble *et al*, 2008; Wu *et al*, 2012). To investigate this phenomenon, we generated a biallelic *RAF1* p.T543M knock‐in HEK293T line using CRISPR/Cas9‐mediated homology‐directed repair (HDR) (Fig 3F). Due to the incomplete nature of HDR, we also obtained complete RAF1^KO/KO^ carrying a homozygous 17‐bp deletion (p.Tyr539AlafsTer26) and compound heterozygous RAF1^T543M/KO^ lines. Clonality of the homozygous RAF1^T543M/T543M^ line was confirmed at the genomic level through restriction digestion with endonuclease *BccI* (Fig 3G). Compared with the WT parental line, Western blotting for endogenous RAF1 showed drastically reduced protein levels that mirrored their respective genotypes. Homozygous RAF1^T543M/T543M^ cells displayed no more than 10–20% of wild‐type RAF1 levels (Fig 3H) while RAF1^T543M/KO^ cells had a further reduction consistent with having only one copy of mutant RAF1 being expressed. Endogenous RAF1^T543M^ protein half‐life could be partially rescued following an 8‐h treatment with the proteasome inhibitor epoxomicin (Fig 3I), arguing that RAF1^T543M^ is inherently unstable and targeted for proteasome‐mediated degradation. This is in agreement with past reports showing that the kinase activity of RAF1 is essential for its proteostasis (Noble *et al*, 2008). Our results indicate that the p.T543M mutation not only diminishes RAF1's kinase activity but also compromises its half‐life.

### RAF1^T543M^ ^/T543M^ knock‐in cells are more sensitive to stress‐induced apoptosis

**Figure 4:**
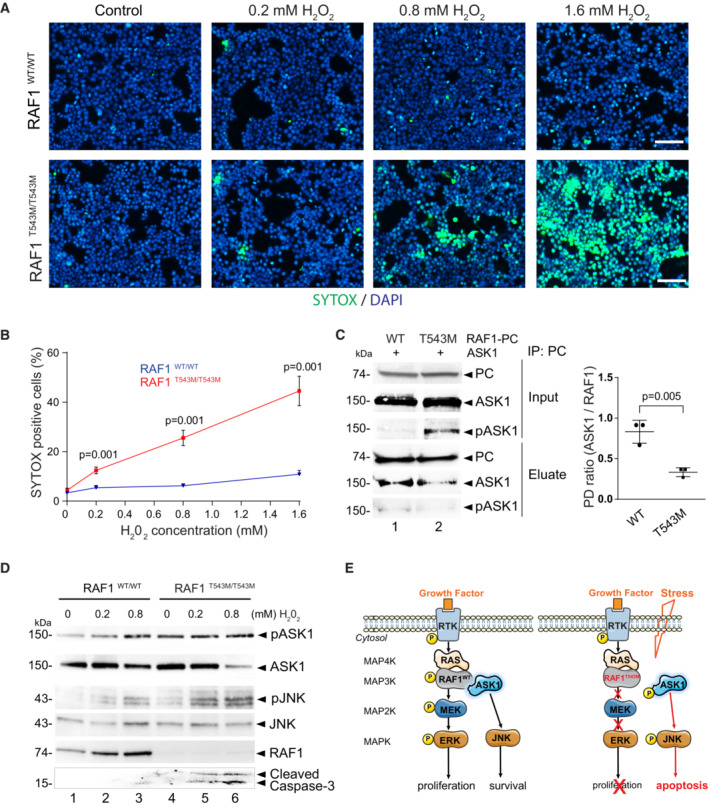
RAF1^T543M/T543M^ knock‐in cells are more sensitive to stress‐induced apoptosis ARepresentative immunofluorescence images of 0.2, 0.8, and 1.6 mM H_2_O_2_ treated RAF1^T543M/T543M^ and RAF1^WT/WT^ HEK293T cells. Green: SYTOX dead cell stain. Blue: DAPI nuclei stain. Scale bar, 100 μm.BDose‐dependent SYTOX signal of RAF1^T543M/T543M^ and RAF1^WT/WT^ HEK293T cells incubated with increasing H_2_O_2_ concentrations for 2 h (*n* = 8 from two independent experiments, error bars indicate mean ± SEM. Two‐tailed Student's *t*‐test).CPC tagged RAF1^WT^ or RAF1^T543M^ were transiently overexpressed along with ASK1 in HEK293T cells for 48 h, immunoprecipitated and blotted for ASK1. Ratio of ASK1 to RAF1 from the pulldown (PD) was quantified in three independent Western blots (Error bars indicate mean ± SEM. Ordinary one‐way ANOVA).DWestern blot documenting endogenous pASK1, pJNK levels and cleaved Caspase 3 following H_2_O_2_‐induced stress in isogenic HEK293T cells expressing endogenous RAF1^T543M/T543M^ or RAF1^WT^. Note that pASK1 is increased without H_2_O_2_ treatment in RAF1^T543M/T543M^ compared with RAF1^WT^.EProposed model of the negative impact of RAF1^T543M/T543M^ on proliferation owing to impaired MAPK/ERK transduction and increased apoptosis due to de‐repression of ASK1 signaling. Source data are available online for this figure.

RAF1 promotes cellular survival by antagonizing apoptosis through MEK/ERK‐independent pathways (Hindley & Kolch, 2002; Desideri *et al*, 2015). Hence, we next examined whether RAF1^T543M/T543M^ cells were more sensitive to apoptotic stimuli. Hydrogen peroxide (H_2_O_2_) was applied to mutant and isogenic control HEK293T cells and levels of apoptosis were measured using SYTOX™ green nucleic acid stain (Xiang *et al*, 2016). Fluorescent images showed that H_2_O_2_‐treated RAF1^T543M/T543M^ cells displayed a greater number of SYTOX™‐positive green nuclei compared with RAF1^WT/WT^ cells (Fig 4A). We observed a dose‐dependent and statistically significant difference in cell death (from 7 to 34%) in RAF1^T543M/T543M^ cells compared with parental wild‐type cells (Fig 4B). These findings suggest that, beyond impaired MEK/ERK signaling that blunts cellular proliferation, the homozygous RAF1^T543M/T543M^ variant heightens cellular sensitivity to stress‐induced apoptosis. To assess this possible mechanism, we next studied the interaction between RAF1, apoptosis signal‐regulating kinase 1 (ASK1), and mammalian Ste20‐like kinase (MST2). ASK1 and MST2 are established pro‐apoptotic molecules normally antagonized by RAF1 via direct binding (Fig 4E; Chen *et al*, 2001; O'Neill *et al*, 2004; Yamaguchi *et al*, 2004; Romano *et al*, 2010).

**Figure EV3:**
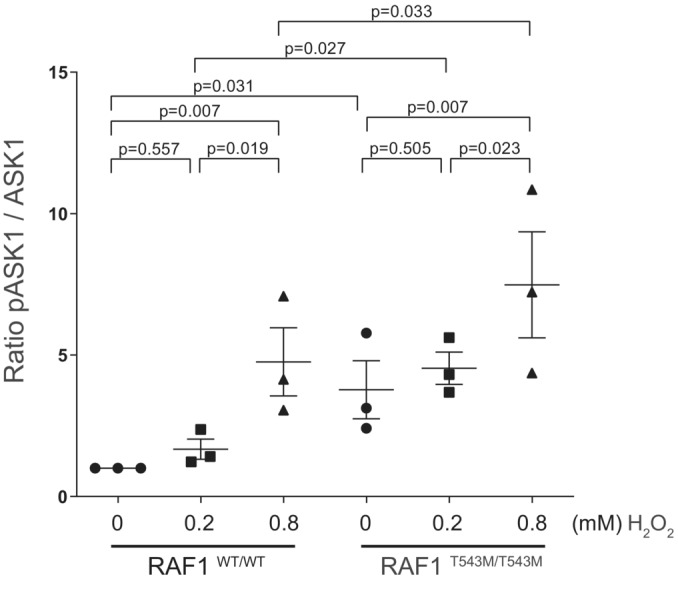
Quantification of pASK1/ASK1 ratio in RAF1^WT^ and RAF1^T543M/T543M^ knock‐in cells Quantification of phosphorylated ASK1, normalized to total ASK1 in three independent western blots (Error bars indicate mean ± SEM. Ordinary one‐way ANOVA).

To test this, RAF1^WT^ or RAF1^T543M^ were overexpressed with ASK1 in HEK293T cells and co‐immunoprecipitated. PC‐tagged RAF1^T543M^ pulled down ASK1 less efficiently than did PC‐tagged RAF1^WT^, suggesting that ASK1 may be disengaged in the presence of RAF1^T543M^. In support of this, we noted that the ratio of active pASK1 to total ASK1 was significantly increased when RAF1^T543M^ was overexpressed (Fig 4C). These results indicate that unbound ASK1 in RAF1^T543M^ cells may be more prone to activation, presumably leading to increased apoptosis upon exposure to stress. To directly examine this, we checked the activation of endogenous ASK1, and other downstream MAPK apoptotic signaling markers, in isogenic WT and mutant RAF1^T543M/T543M^ HEK293T cells. A dose‐dependent increase in activated pASK1 was observed that was significantly greater in RAF1^T543M/T543M^ cells (Fig EV3). This was paralleled by an increase in c‐Jun N‐terminal kinase (JNK) phosphorylation and cleaved Caspase‐3 (Fig 4D, lane 4 and 5). Taken together, this confirms that stressed RAF1 mutant cells are more susceptible to apoptosis which we propose may be in part driven by ASK1 de‐repression.

## Discussion

Collectively, our data unveil the genetic and molecular etiology of a novel neonatal lethal malformation syndrome with progeroid features and reveal the importance of the proto‐oncogene RAF1 during human development.

Our lines of investigations highlighted several ways by which this germline RAF1^T543M^ mutation may behave as a strong hypomorphic allele underlying the neonatal lethal syndrome described herein. Our functional tests suggest that this RAF1^T543M^ variant: (i) is not phosphorylated at important regulatory residues including Ser259 and Ser338, (ii) is unable to transduce RAS‐mediated MAPK signaling towards MEK/ERK substrates *in vitro* and *in vivo*, (iii) is inherently unstable and prone to proteasome‐mediated degradation and (iv) is unable to block stress‐induced apoptosis, possibly by disrupting the ASK1/JNK signaling pathway.

RAF1's kinase activity is intrinsically linked to its own phosphorylation status. Tyrosine kinases such as SRC6, serine/threonine kinases such as casein kinase 2 (CK2), and RAF1 itself have been shown to impact RAF1's phosphorylation (Matallanas *et al*, 2011). Here, we provide evidence that Thr543 (which is mutated to a methionine in this consanguineous family) and its neighboring Tyr539 likely do not regulate RAF1 activity in a phosphorylation‐dependent manner as substitutions of either residue with phosphorylation‐inert residues have no effect on RAF1 activity. In support of this, the homologous Tyr539 residue in zebrafish is a phenylalanine, while the homologous Thr543 residue in mice is an alanine. Taken together, our results suggest that Thr543 is not an additional human‐specific regulatory phosphorylation site that would be impacted in this family. Instead, we contend that the identified p.T543M missense mutation exerts its pathogenicity through an alternative mechanism, involving deficient kinase activity, reduced protein stability, and impaired interactions with ASK1.

Apart from the established role of RAF1 in MAPK signaling—which is reduced in RAF1^T543M^—it has also been found to perform several kinase‐independent functions. These include the inhibition of ROCK2 (Ehrenreiter *et al*, 2005) or promotion of cell survival by antagonizing the proapoptotic kinases ASK1 (Yamaguchi *et al*, 2004) and MST2 (O'Neill *et al*, 2004). Here, we observed that the RAF1^T543M^ protein is markedly unstable compared with its wild‐type counterpart. This instability significantly heightened cellular sensitivity to redox stress‐induced apoptosis, accompanied by increased ASK1 activity, which may be in part driven by the loss of direct RAF1‐mediated repression.

Given the central role played by RAF1 in transducing the cell‐growth‐promoting activities of numerous mitogens, it is fitting that a loss‐of‐function RAF1 mutant allele would have severe consequences, especially during embryogenesis when the organism experiences exponential growth. The developmental syndrome described herein is reminiscent of Acro‐cardio‐facial syndrome (ACFS, MIM600460). Acro‐cardio‐facial syndrome is a very rare, genetically orphan disorder characterized by split‐hand/split‐foot malformations (SHFM), congenital heart defects (CHD), facial anomalies, cleft lip/palate, and genital anomalies. First described by Richieri Costa & Orquizas (1987), a total of 11 patients from nine unrelated families have been reported (Richieri‐Costa, 1987; Giannotti *et al*, 1995; Guion‐Almeida *et al*, 2000; Mingarelli *et al*, 2005; Sivasli *et al*, 2007; Kariminejad *et al*, 2008; Tanpaiboon *et al*, 2009; Toschi *et al*, 2012; Hudson *et al*, 2014; Table 1). This syndrome is associated with very poor life expectancy, most patients surviving only a few hours or months. To date, no genetic cause has been established, although an autosomal‐recessive inheritance pattern is supported by both consanguinity and recurrence in sibs born to unaffected parents (Richieri Costa & Orquizas, 1987; Giannotti *et al*, 1995; Kariminejad *et al*, 2008).

Notably, the cutaneous, cardiac, and limb anomalies of the two affected siblings we described above are similar to those documented in *Raf1* mutant animal models. Wojnowski *et al* (1998) noted thinner and less differentiated dermal and epidermal layers in *Raf1* ^−/−^ mice, while Yamaguchi *et al* (2004) found that heart‐specific *Raf1* ^−/−^ mice exhibited cardiac dysfunction and apoptosis. More recently, the recessive‐acting *wingless2* mutation found in *Gallus gallus*—which results in a phenotype strikingly similar to this syndrome with truncated limbs, craniofacial, cardiac, and skin/feather defects—was linked to a homozygous premature stop codon in *Raf1* (Youngworth & Delany, 2019).

Additionally, these phenotypes have been observed in animal models containing mutations of other FGF signaling members. Mutations in mice of RAF1's downstream effector ERK2 (Frémin *et al*, 2015) and its upstream regulator TP63 (Mills *et al*, 1999; Yang *et al*, 1999; Rouleau *et al*, 2011; Ferone *et al*, 2012) result in lethality with similar craniofacial, cardiovascular, and limb formation defects. These phenotypes varied in severity depending on global ERK activity (Frémin *et al*, 2015). Likewise, increasing severities of limb formation defects corresponded to decreasing levels of FGF signaling (ranging from loss of a single digit to entirely truncated limbs) in mice with combinatorial knockouts of *Fgfr* and *Fgf* gene family members in the apical ectodermal ridge (AER) (Mariani *et al*, 2008; Yu & Ornitz, 2008). The SHFM characteristic of ACFS and our patient could similarly reflect on a dampened FGF10/FGF8 positive feedback loop between the embryonic limb bud's mesoderm and its overlying AER, leading to reduced outgrowth of the limbs (Mariani *et al*, 2017). We note too that patients with mutations in components of the FGF pathway also present with highly overlapping combinations of craniofacial, cardiovascular, and limb phenotypes, collectively termed neuro‐cardio‐facial‐cutaneous syndromes (Bentires‐Alj *et al*, 2006), suggesting ACFS phenotypes could similarly be interpreted as resulting from defective FGF signaling.

Given the clinical similarity of RAF1 deficiency to ACFS, it may be worth checking for RAF1 variants as well as upstream regulators and downstream effectors in previously reported ACFS cases. While we propose RAF1 insufficiency as a plausible genetic etiology for ACFS, it may not necessarily be classified as a RASopathy, which typically involve gain‐of‐function rather than loss‐of‐function mutations. Instead, this case should be considered as a progeroid variant of ACFS that illustrates the physiological importance of the proto‐oncogene RAF1, and perhaps the wider FGF signaling pathway, during human development.

In particular, unlike other documented ACFS instances (Table 1), we noticed distinct progeroid features such as the hypoplastic viscerocranium, prominent neurocranium, dry, lax, translucent skin, and loss of subcutaneous fat tissue in our patient. The dermal features and subcutaneous fat loss, commonly observed with chronological aging, suggest that RAF1 might play an important role in physiological aging, given its observed role in regulating cellular proliferation and apoptosis (Fig 4). While beyond the scope of this study, this observation warrants further study of a hitherto unexplored role of RAF1 in chronological aging. Like for other progeroid conditions such as *PYCR1* inactivation in De Barsy syndrome, this RAF1 loss‐of‐function phenotype provides another illustration of how genes that are hijacked in the context of cancer may inversely cause premature aging when inactivated (Reversade *et al*, 2009; Ding *et al*, 2020).

## Materials and Methods

### Human subjects

All procedures were performed following informed consent and approval from patients and relatives and obtained in accordance with the Declaration of Helsinki and the Department of Health and Human Services Belmont Report. Peripheral blood samples were collected from six family members (II:1, II:3, III:2, III:4, III:5, III:6). Blood DNA was extracted using standard methods. All human studies were reviewed and approved by the Turkish and Singaporean institutional review board (A*STAR IRB #2019‐087 and Koç University 2015.120.IRB2.047). Parents of the patient gave informed consent to publish their deceased child photographs.

### Genetic analysis

SNP and linkage analysis were carried out on five family members (II:2, II:3, III:2, III:4 and III:5) using Affymetrix GeneChip Human Mapping 250K SNP microarray. Total of five family members were genotyped using Illumina HumanCore‐12v1 BeadChips following the manufacturer's instructions. Call rates were above 99%; gender and relationship were verified using Illumina GenomeStudio software. Linkage analysis was performed by searching for shared regions in the affected individual using custom programs written in Mathematica (Wolfram Research, Inc.). Whole‐exome sequencing was performed on DNA from proband III:2. Briefly, the exome library was prepared on an Ion OneTouch System and sequenced on an Ion Proton instrument (Life Technologies) with one Ion PI chip. Sequence reads were aligned to the human GRCh37/hg19 assembly (UCSC Genome Browser). Variants were filtered for common SNPs against the NCBI's “common and no known medical impacts” database (ClinVar), the Exome Aggregation Consortium (ExAC) Browser, the NHLBI Exome Sequencing Project, and an in‐house database of 197 sequenced samples (Dataset EV1). Sanger sequencing was performed using primers flanking the mutations (Forward 5′‐TAATGAAAGGGACAGCCTGG and Reverse 5′‐CTCCCACCTTATATTGCCATC).

### Cell culture

HEK293T (ATCC CRL‐3216, RRID: CVCL_0063) wild‐type and mutant were grown in Dulbecco's modified Eagle's medium (DMEM) with 10% Fetal Bovine Serum (FBS, Hyclone), 1% L‐glutamine (Invitrogen) at 37°C in a humidified atmosphere of 5% CO_2_ plates. They were regularly tested for mycoplasma using the MycoAlert™ Mycoplasma Detection Kit (Lonza). At various time points, cells were either fixed in 4% paraformaldehyde for further immunofluorescence analysis or collected for protein extraction.

### Overexpression assay *in vitro*

Full‐length RAF1 cDNA (gift from Dhandapany's group) and ASK1 cDNA were cloned into pCS2^+^ plasmid backbone and site‐directed mutagenesis (QuikChange Site‐Directed Mutagenesis kit, Stratagene) performed. Prior to transfection, HEK293T cells were plated onto poly‐L‐lysine (Sigma) precoated six‐well plates. At ~ 90% confluence, cells were transfected with constructs using Lipofectamine 2000 (Invitrogen). Amounts of each construct transfected were adjusted to approximate equal RAF1 levels at collection. 48 h after transfection, cells were serum‐starved for 16 h by replacing with FBS‐free media. Cells were then exposed for 15 min to FBS‐containing media to initiate MAPK signaling, and harvested immediately on ice, for 30 min, using cold RIPA buffer supplemented with protease inhibitor cocktail (Roche) and phosphatase inhibitor (Sigma). Lysates were centrifuged at 4°C, and supernatant collected for analysis.

### Antibodies

All specific antibodies were used between 1 and 10 μg/ml for Western blotting and are commercially available: α‐ACTIN (1:2,000 dilution, Merck Millipore, Mab1501R, RRID:AB_2223041), c‐RAF (1:2,000 dilution, BD Biosciences, 610152, RRID:AB_397553), phospho‐c‐RAF(Ser259) (1:1,000 dilution, Cell Signaling, 9421, RRID:AB_330759), phospho‐c‐RAF (Ser338) (1:1,000 dilution, Cell Signaling, 9427, RRID:AB_2067317), pMEK1/2 (1:1,000 dilution, Cell Signaling, 9154, RRID:AB_2138017), total MEK1/2 (Cell Signaling, 9122, RRID:AB_823567), pERK1/2 (1:1,000 dilution, Cell Signaling, 4376, RRID:AB_331772), total ERK1/2 (1:1,000 dilution, Cell Signaling, 4695, RRID:AB_390779), ASK1 (1:500 dilution, Cell Signaling, 8662, RRID:AB_11220434) and pASK1 (1:1,000 dilution, Cell Signaling, 3765, RRID:AB_2139929), JNK (1:1,000 dilution, Cell Signaling, 9252, RRID:AB_2250373); pJNK (1:1,000 dilution, Cell Signaling, 9251, RRID:AB_331659).

### RAF1 mutant cells generation

An sgRNA against exon 15 of RAF1 (20 bp sgRNA sequence: 5′‐ATTGTATGAACTGATGACGG‐3′) was cloned into a plasmid for coexpression with SpCas9‐T2A‐Puro (pSpCas9(BB)‐2A‐Puro (PX459) V2.0, Addgene plasmid # 62988) according to Ran *et al* (2013). This sgRNA was chosen as it contains the target c.1628C nucleotide 1 bp from the Cas9 cut site, for maximal substitution efficiency. (Paquet *et al*, 2016) For HDR, a single‐stranded oligodeoxynucleotide (ssODN) with 40 bp homology arms flanking the desired c.1628C>T (p.T543M) and c.1632G>C (PAM disruption, synonymous) mutations was designed. Successful c.1628C>T HDR would create a BccI restriction site. ssODN sequence:

5’‐GATGTCTACTCCTATGGCATCGTATTGTATGAACTGATGATGGGCGAGCTTCCTTATTCTCACATCAACAACCGAGATCAG‐3′.

A 70% confluent 12‐well plates of HEK293T cells was transfected with 625 ng RAF1 sgRNA+PX459 and 125 μl of 10 μM ssODN using Lipofectamine 2000 (Life technologies), following the manufacturer's protocol. 24 h post‐transfection, media were replaced with puromycin to a final concentration of 2 μg/ml for 4 days of selection. Surviving cells were then serially diluted and plated into 96‐well plates at 0.5 cells/well for clonal expansion. Wells with a single colony were Sanger‐sequenced during splitting and successful HDR confirmed by PCR and BccI restriction digest. Due to the low frequency of HDR, the majority of clones contained indels (39/43 clones analyzed), with a single compound heterozygous 2 bp indel/c.1628C>T and a single homozygous c.1628C>T clone obtained. A clone with homozygous 17 bp indels was retained as RAF1^KO/KO^ cells. The compound heterozygote was verified by TA‐cloning RAF1 exons 15–16, and subsequently Sanger‐sequencing individual transformed colonies, yielding a ~ 1:1 mix of two alleles. pSpCas9(BB)‐2A‐Puro (PX459) V2.0 was a gift from Feng Zhang (Addgene plasmid #62988; http://n2t.net/addgene:62988; RRID:Addgene_62988).

### Cell viability assay

Wild‐type or knock‐in HEK293T cells were plated in 12‐well plates precoated with poly‐L‐lysine (Sigma). Upon confluence, cells were stimulated with a range of H_2_O_2_ (0.2, 0.8 or 1.6 mM) and further incubated at 37°C for 2 h. H_2_O_2_ concentrations were carefully chosen to induce minimum apoptosis in HEK293T wild‐type cells. Cell death was assessed by adding SYTOX® Green Nucleic Acid Stain (1/1000, Invitrogen, S7020) in the culture for 15 min. Subsequently, cells were incubated with DAPI. Images were taken with a Thermo Fisher Scientific EVOS® FL Cell Imaging System. SYTOX green and DAPI blue‐positive nuclei were counted automatically using ImageJ and data were expressed in percentage per number total of nuclei. Two independent assays were performed, and four representative images were analyzed for each condition.

### Biochemistry

For Western blotting, cells or *Xenopus* embryos were lysed in RIPA buffer supplemented with protease inhibitor cocktails (Roche) and phosphatase inhibitors (Sigma). Extracted proteins were separated by electrophoresis on SDS polyacrylamide gels with DTT (Bio‐Rad), followed by a TransBlot Turbo transfer (Bio‐Rad) onto polyvinylidene difluoride (PVDF) membranes. Membranes were incubated with primary antibodies in 5% BSA or 5% milk in Tris‐Buffered Saline‐Tween (TBST) according to the manufacturer's instructions for 2 h at room temperature or 4°C overnight on an orbital shaker, followed by three washes of 15 min in TBST. Following a 2‐h incubation with the respective secondary antibodies, membranes were washed in TBST and developed using West Pico, Dura, or Femto chemiluminescence reagents (Thermo Fisher). Global phosphorylation was measured using a 7.5% SuperSep™ Phos‐tag™ gel. Protein samples were run in Tris‐Glycine SDS buffer according to the manufacturer's instructions followed by a wet transfer method to a PVDF membrane in 20% methanol Tris‐Glycine SDS buffer. The membrane was immunoblotted in the same manner as described for Western blots above. To block protein ubiquitin‐dependent degradation 100 nM epoxomicin (Sigma) was added to cultured cells for 8 or 24 h. Quantification of Western blot was performed by ImageJ. Immunoprecipitation was performed by incubating samples from HEK293T cells co‐expressing ASK1 and Protein C‐tagged RAF1^WT^ or RAF1^T543M^ with Anti‐Protein C affinity matrix (Roche, 11815024001) for 4 h at 4°C on a rotator. After a series of washes, bound proteins were eluted with Laemmli buffer at 95°C.

### *Xenopus* methods

Protocols for *Xenopus laevis* fertilization, micro‐injections, mRNA synthesis, and whole‐mount in situ hybridization (WISH) are on our protocol's website page (see http://www.reversade.com‐a.googlepages.com/protocols/). 200 pg of either RAF1^WT^, RAF1^T543M^, RAF1^L603P^, or RAF1^S257L^ mRNA were injected into 4‐cell stage *Xenopus laevis* embryos, and harvested at various developmental stages for subsequent protein extraction or WISH analyses. Images of embryos were captured with a stereomicroscope equipped with an integrated digital camera (Leica; M205 FA). All experiments were conducted according to the protocols approved by the local authority for animal welfare (IACUC Protocol #181395).

### Quantification and statistical analysis

All statistical tests were carried out using Prism 7 or Excel unless otherwise stated. Information on statistical tests used for each assay and number of samples are detailed in the Figure legends and in Materials and Methods sections. The values are presented as mean ± SEM. *P*‐value < 0.05 was considered statistically significant. All of the experiments were carried out in at least three technical replicates. Simple randomization was applied for treated samples or injected animals. All the samples were included and analyses were performed without blinding.

## Author contributions

**Samantha Wong:** Data curation; formal analysis; validation; investigation; methodology; writing – original draft; writing – review and editing. **Yu Xuan Tan:** Data curation; formal analysis; validation; methodology; writing – original draft; writing – review and editing. **Abigail Yi Ting Loh:** Validation; investigation; writing – review and editing. **Kiat Yi Tan:** Investigation. **Zainab Aziz:** Investigation. **Engin Özkan:** Investigation. **Hülya Kayserili:** Resources; investigation; writing – review and editing. **Nathalie Escande‐Beillard:** Conceptualization; formal analysis; supervision; validation; investigation; visualization; methodology; writing – original draft; project administration; writing – review and editing. **Bruno Reversade:** Conceptualization; resources; supervision; funding acquisition; validation; methodology; writing – original draft; writing – review and editing. **Stanley F Nelson:** Data curation. **Hane Lee:** Data curation.

## Disclosure and competing interests statement

The authors declare that they have no conflict of interest.

## Supporting information



Expanded View Figures PDF

Dataset EV1

PDF+

Source Data for Figure 1

Source Data for Figure 2

Source Data for Figure 3

Source Data for Figure 4

## Data Availability

Exome sequencing and analysis were performed in 2012 at the Department of Human Genetic (UCLA, USA). The Raw data in form of BAM files could not be retrieved while the summary findings are listed in Dataset EV1. Limited genomic DNA from the proband and parents can be made available upon request for further analyses. The homozygosity mapping and Sanger sequencing validation generated in this study are presented in Dataset EV1 and Fig EV1.
