# Supplementary material for: RAF1 deficiency causes a lethal syndrome that underscores RTK signaling during embryogenesis
Source: EMBO Mol Med. 2023 Apr 17;15(5):e17078. doi: 10.15252/emmm.202217078 (PMC10165362; doi:10.15252/emmm.202217078)
Supplement: Supplementary file 1 — Expanded View Figures PDF [file EMMM-15-e17078-s007.pdf]

Expanded View Figures

A

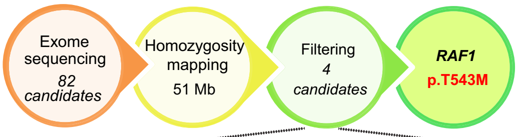

Gene	Location	HGVSc	HGVSp	gnomAD	dbSNP	SIFT	PolyPhen	Condel	M-CAP	Conservation
RAF1	3:12626661	c.1628C>T	p.Thr543Met	het:0 , Hom:0	none	deleterious	possibly damaging	deleterious	0.818	3.91
PLIN2	9:19116484	c.1076A>G	p.Asn359Ser	het: 0.001035, Hom:1	rs144179472	tolerated	benign	neutral	0.002	0.412
ABL1	9:133759712	c.2092T>G	p.Ser698Ala	het: 0.0003719 , Hom:0	rs201278085	tolerated	benign	neutral	0.005	-2.08
C16orf93	16:30772988	c.82G>A	p.Ala28Thr	het:0.01061, Hom:11	rs201918052	tolerated	possibly damaging	neutral	N.A	-1.72

B

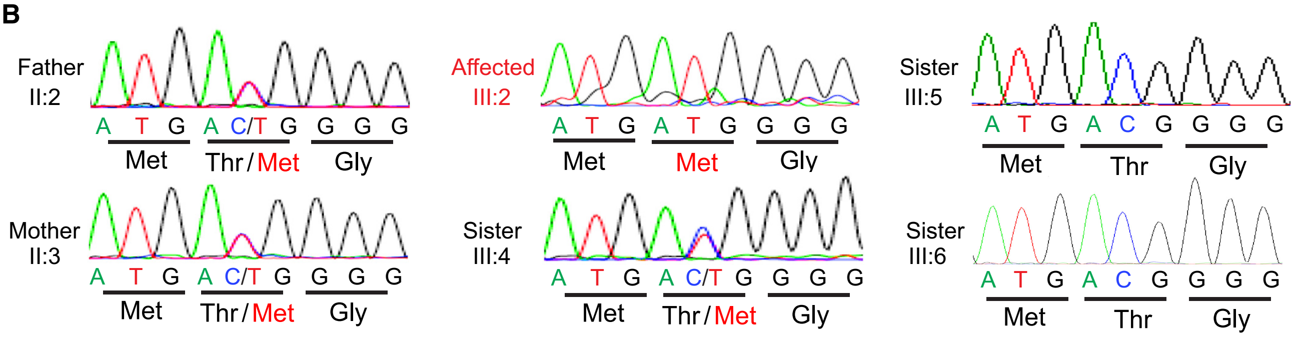

C

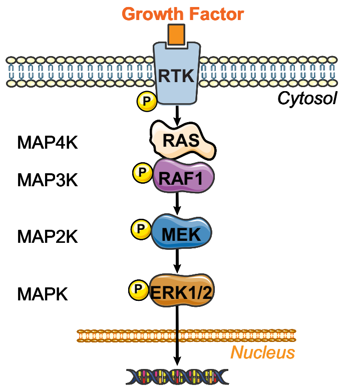

D

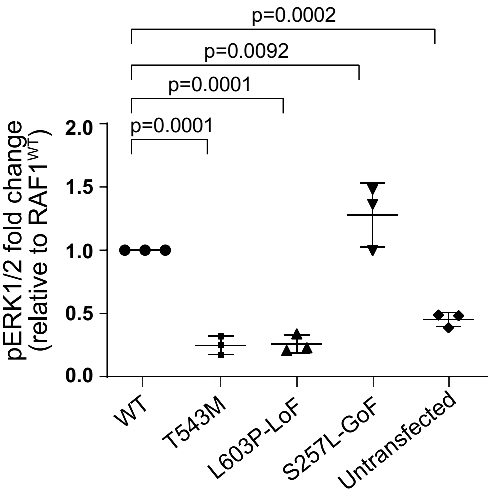

Figure EV1. RAF1^{T543M} allele behaves as a loss-of-function mutation *in vitro*.

A Genetic strategy. Combination of exome sequencing and homozygosity mapping. resulted in four candidate genes. Germline homozygous variants in *PLIN2*, *ABL1*, and *C16orf93* were filtered out due to their low phylogenetic conservation and low predicted deleterious potential.

B Sanger sequencing chromatogram showing variant c.1628C>T (p.T543M) segregating with disease: homozygous in the proband (III:2), heterozygous in the parents (II:2 and II:3) and one sister (III:4) and absent in two other siblings (III:3 and III:6).

C Schematic representation of ERK1/2 MAP Kinase pathway.

D Quantification of phosphorylated ERK1/2, normalized to WT RAF1 in three independent Western blots (Error bars indicate mean ± SEM. Ordinary one-way ANOVA).

© 2023 The Authors

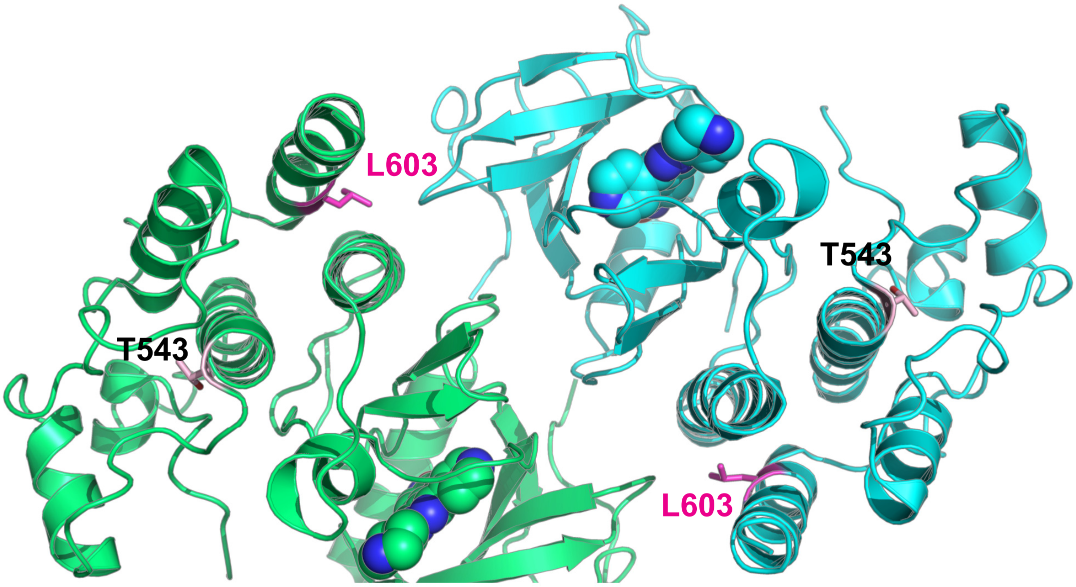

Figure EV2. Loss-of-function mutation p.L603P impairs RAF1 kinase activity.

Position of Leu603 and Thr543 in the RAF1 homodimer (PDB:3OMV). The p.L603P mutation causes an impairment of kinase activity, likely through destabilizing the αH helix and the helical bundle made by the αC and αH helices, and may also affect the dimer stability. Thr543 is located in the αE helix.

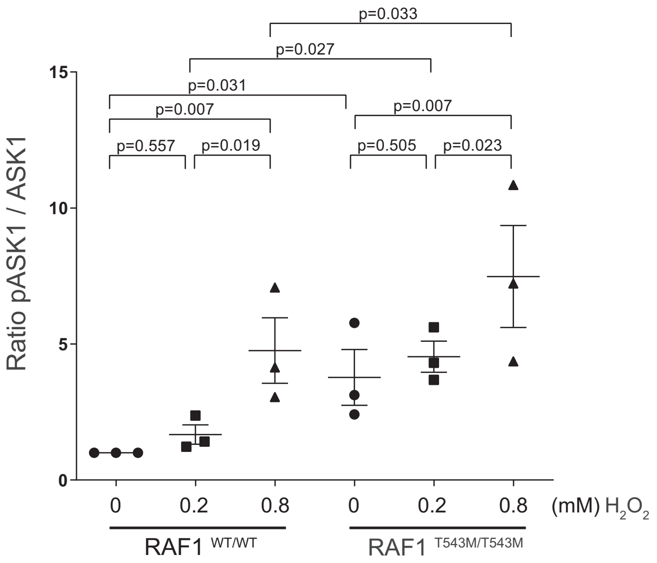

Figure EV3. Quantification of pASK1/ASK1 ratio in $RAF1^{WT}$ and $RAF1^{T543M/T543M}$ knock-in cells.

Quantification of phosphorylated ASK1, normalized to total ASK1 in three independent western blots (Error bars indicate mean ± SEM. Ordinary one-way ANOVA).
