# Supplementary figures and images for: RAF1 deficiency causes a lethal syndrome that underscores RTK signaling during embryogenesis
Source: EMBO Mol Med. 2023 Apr 17;15(5):e17078. doi: 10.15252/emmm.202217078 (PMC10165362; doi:10.15252/emmm.202217078)

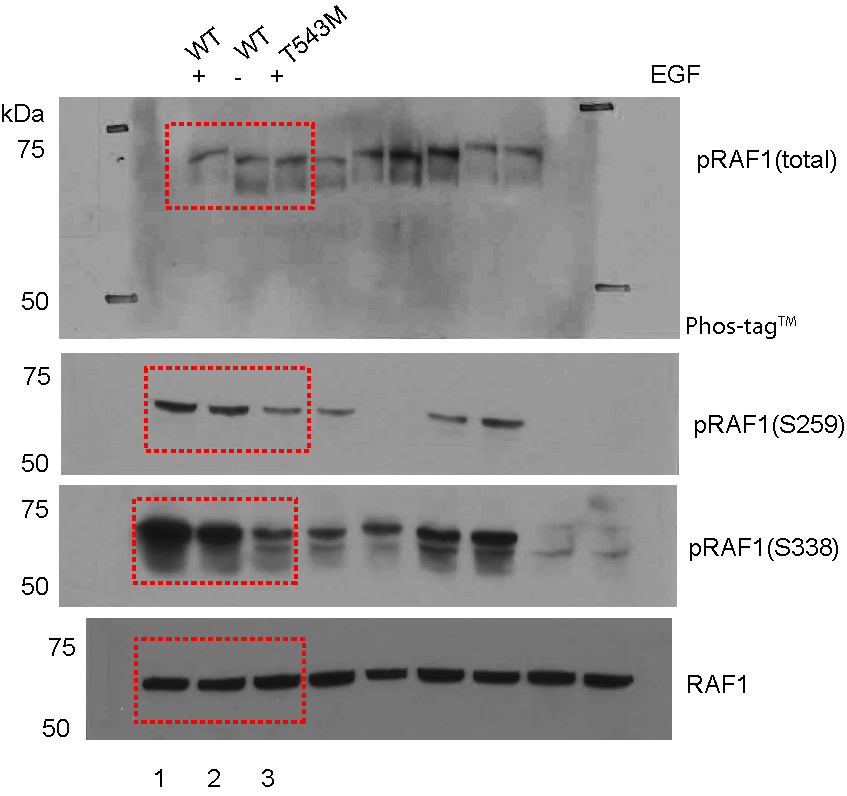

Supplement: Supplementary file 4 — Source Data for Figure 1 [file EMMM-15-e17078-s001.zip › Figure 1/1E/Fig 1E_western blot.tif]

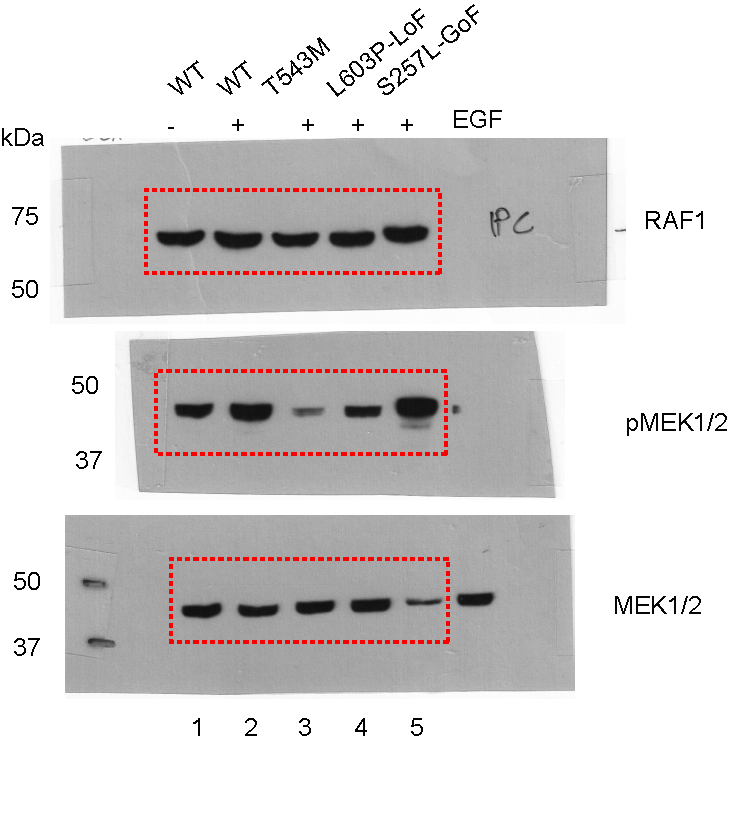

Supplement: Supplementary file 4 — Source Data for Figure 1 [file EMMM-15-e17078-s001.zip › Figure 1/1F/Fig 1F_Western blot.tif]

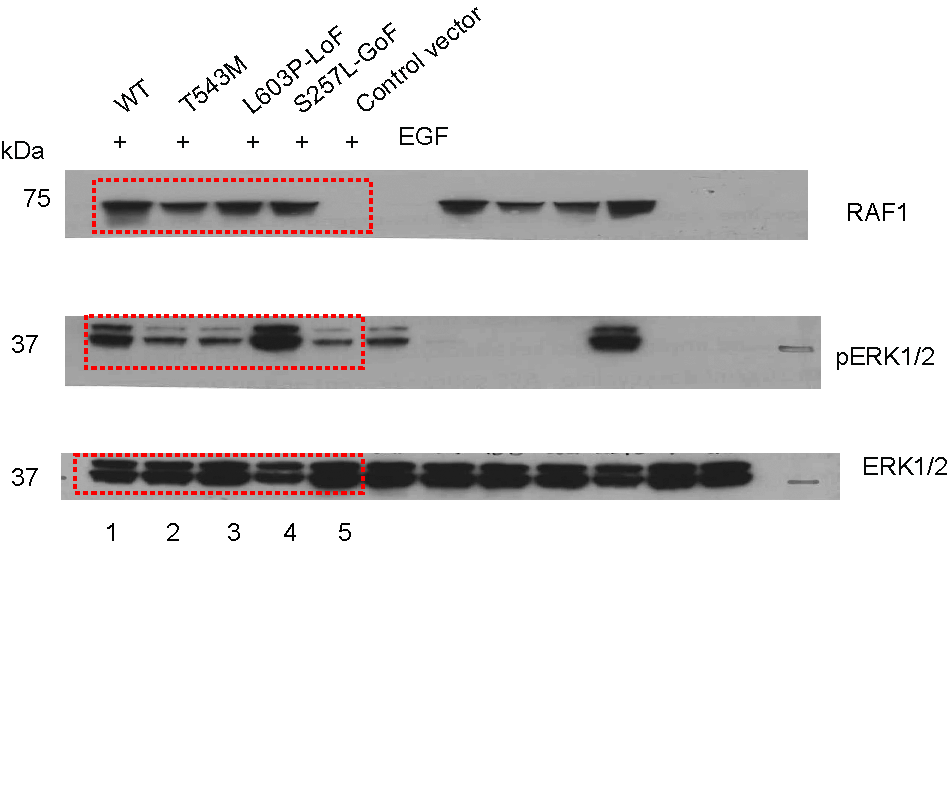

Supplement: Supplementary file 4 — Source Data for Figure 1 [file EMMM-15-e17078-s001.zip › Figure 1/1G/Fig 1G_Western blot.tif]

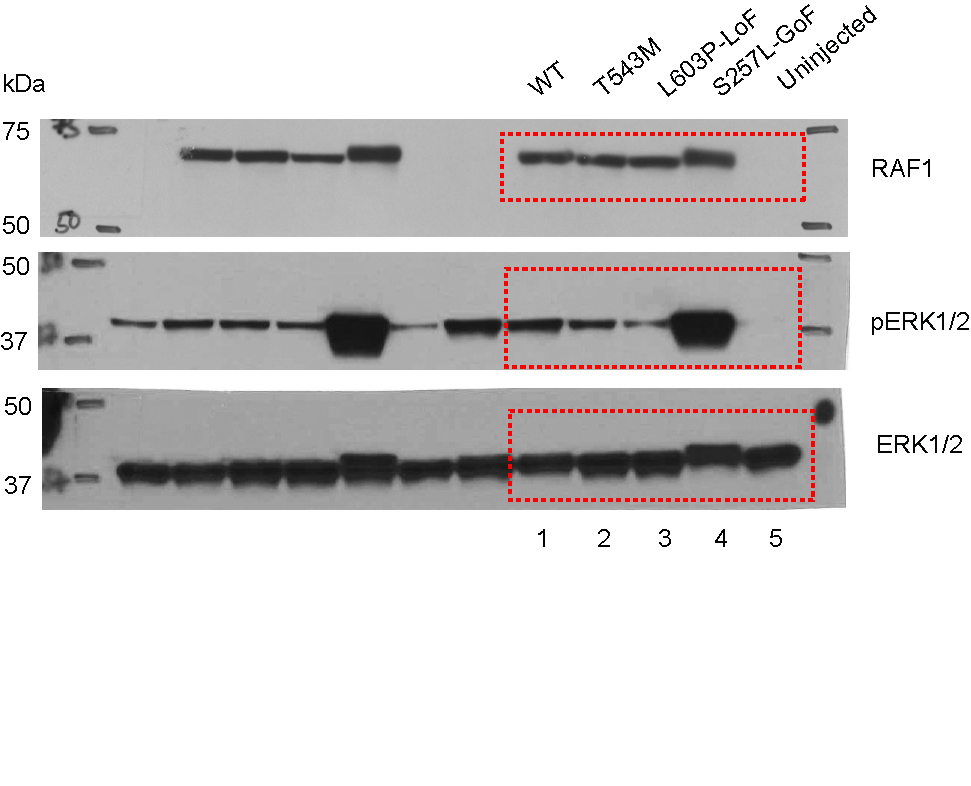

Supplement: Supplementary file 5 — Source Data for Figure 2 [file EMMM-15-e17078-s002.zip › Figure 2/2A_western blot/Fig 2A_western blot.tif]

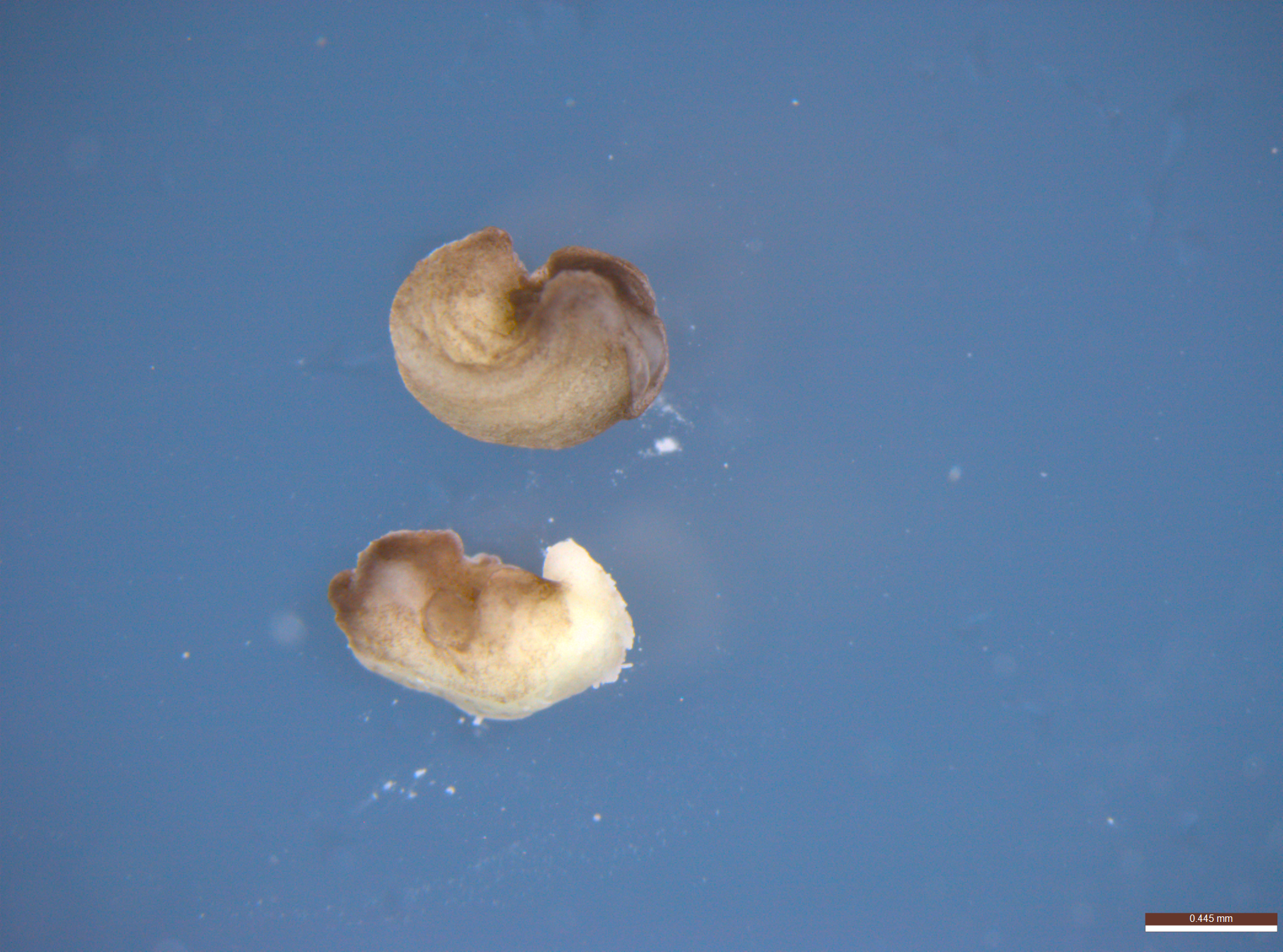

Supplement: Supplementary file 5 — Source Data for Figure 2 [file EMMM-15-e17078-s002.zip › Figure 2/2C_Images embryos/2C GoF image.tif]

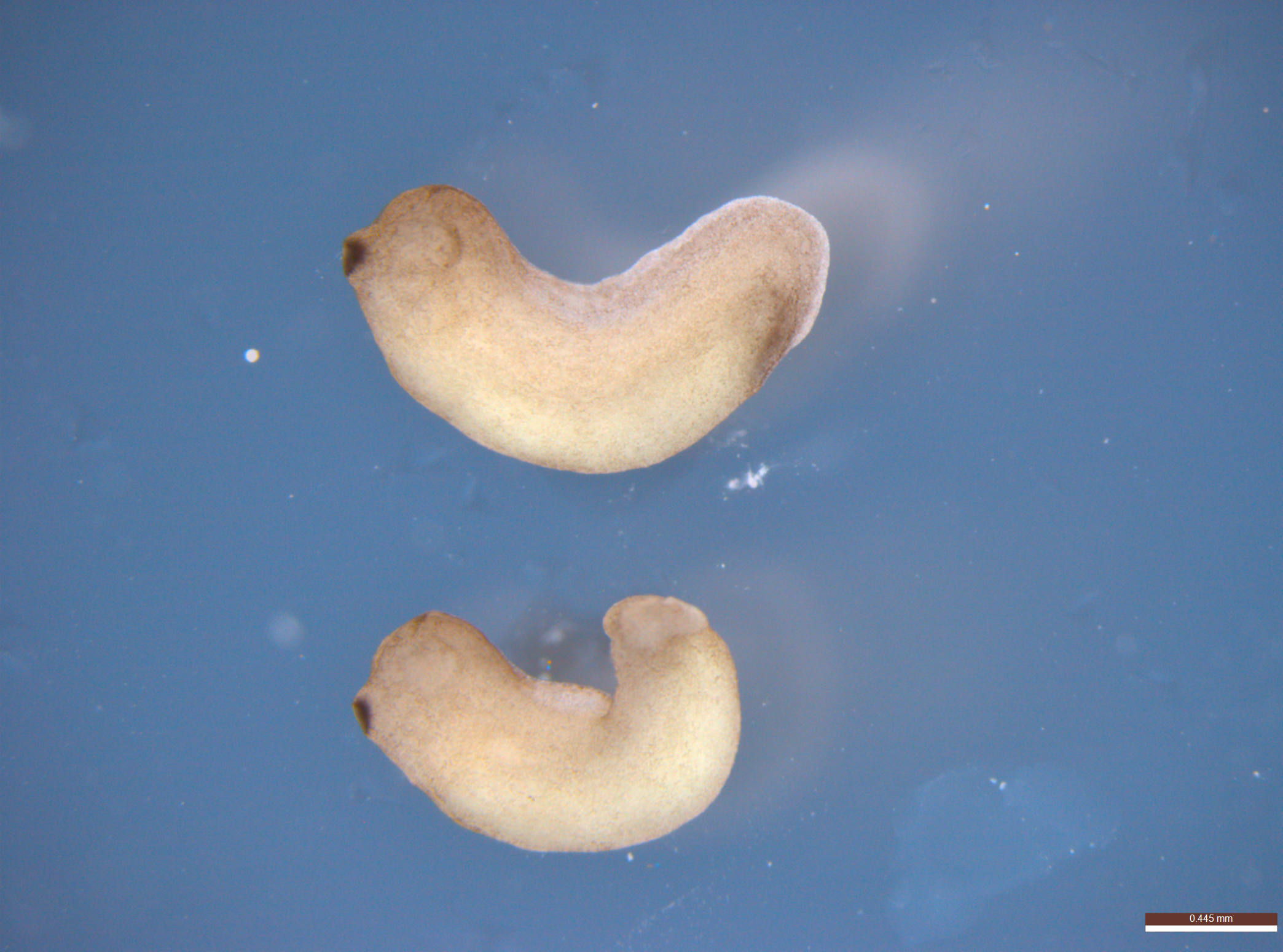

Supplement: Supplementary file 5 — Source Data for Figure 2 [file EMMM-15-e17078-s002.zip › Figure 2/2C_Images embryos/2C LoF image.tif]

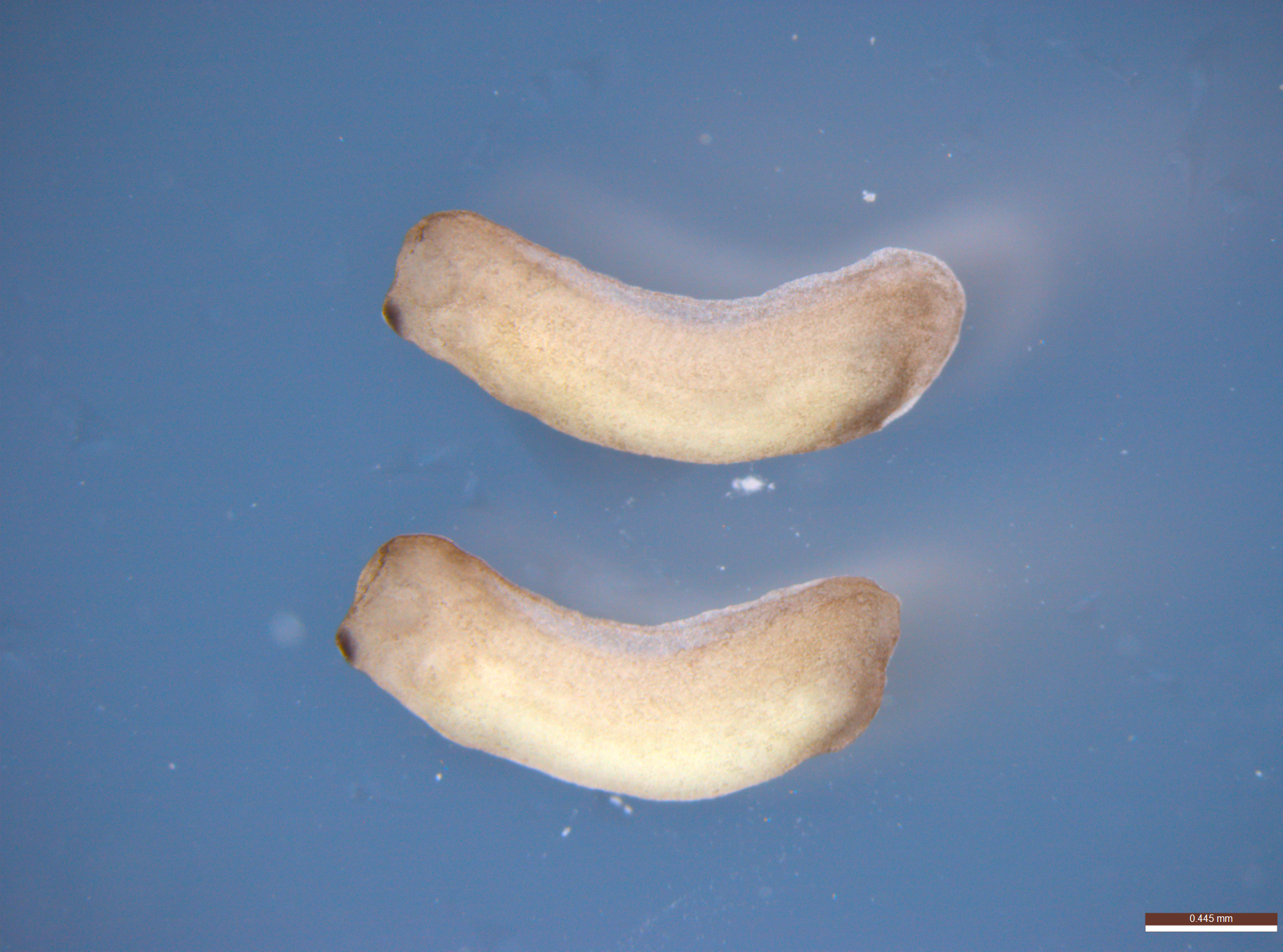

Supplement: Supplementary file 5 — Source Data for Figure 2 [file EMMM-15-e17078-s002.zip › Figure 2/2C_Images embryos/2C T543M image.tif]

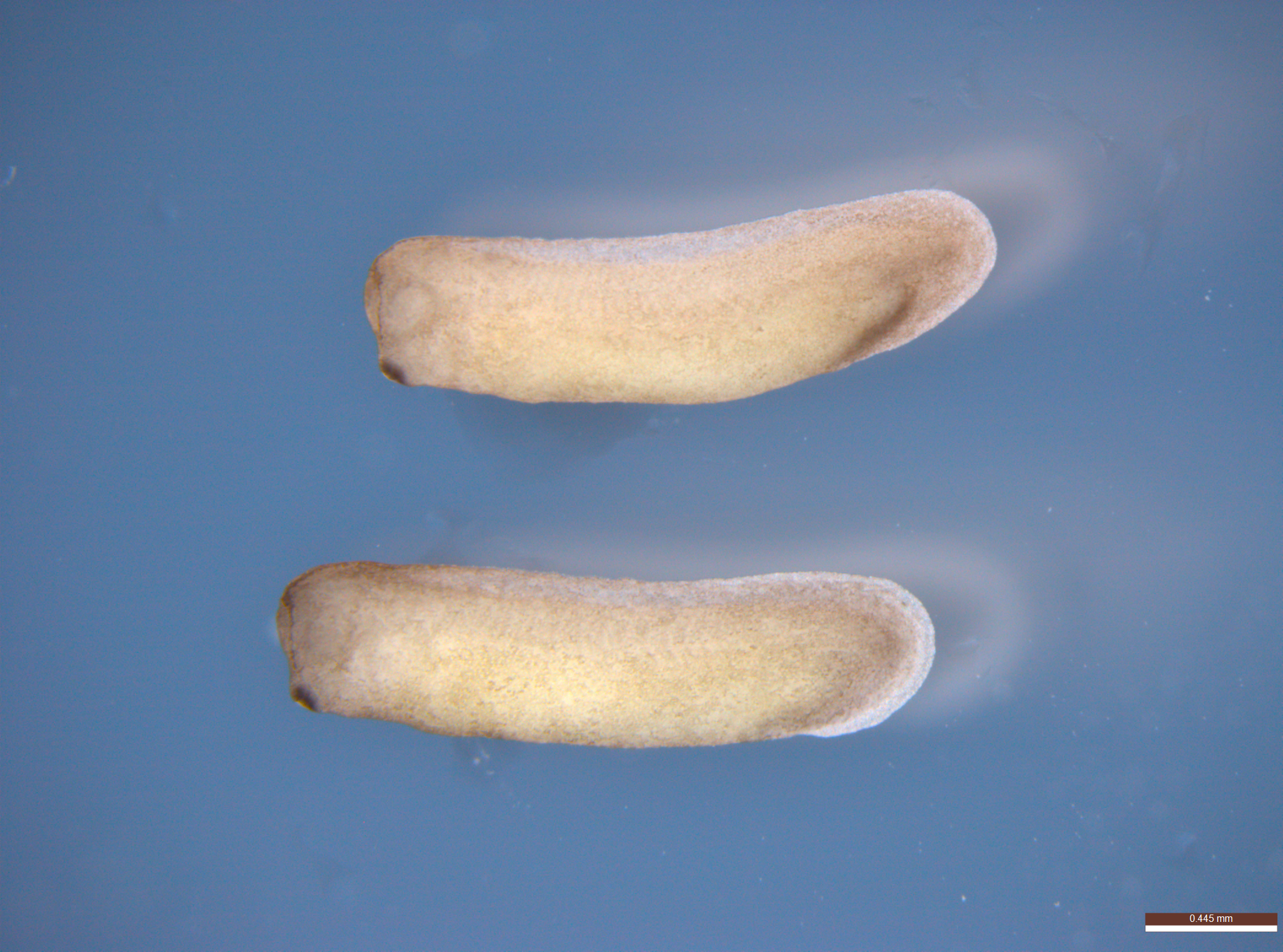

Supplement: Supplementary file 5 — Source Data for Figure 2 [file EMMM-15-e17078-s002.zip › Figure 2/2C_Images embryos/2C uninjected image.tif]

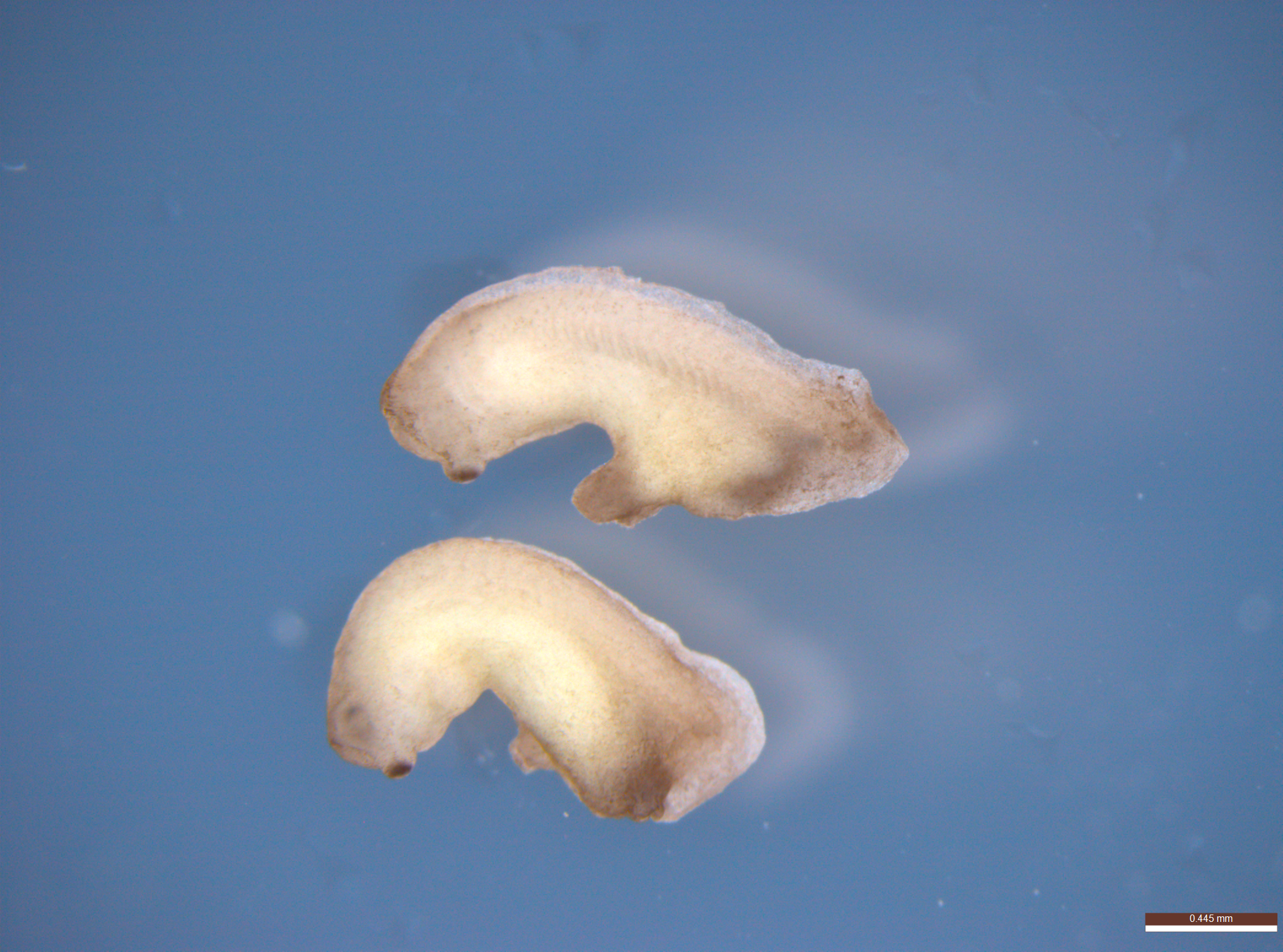

Supplement: Supplementary file 5 — Source Data for Figure 2 [file EMMM-15-e17078-s002.zip › Figure 2/2C_Images embryos/2C WT image.tif]

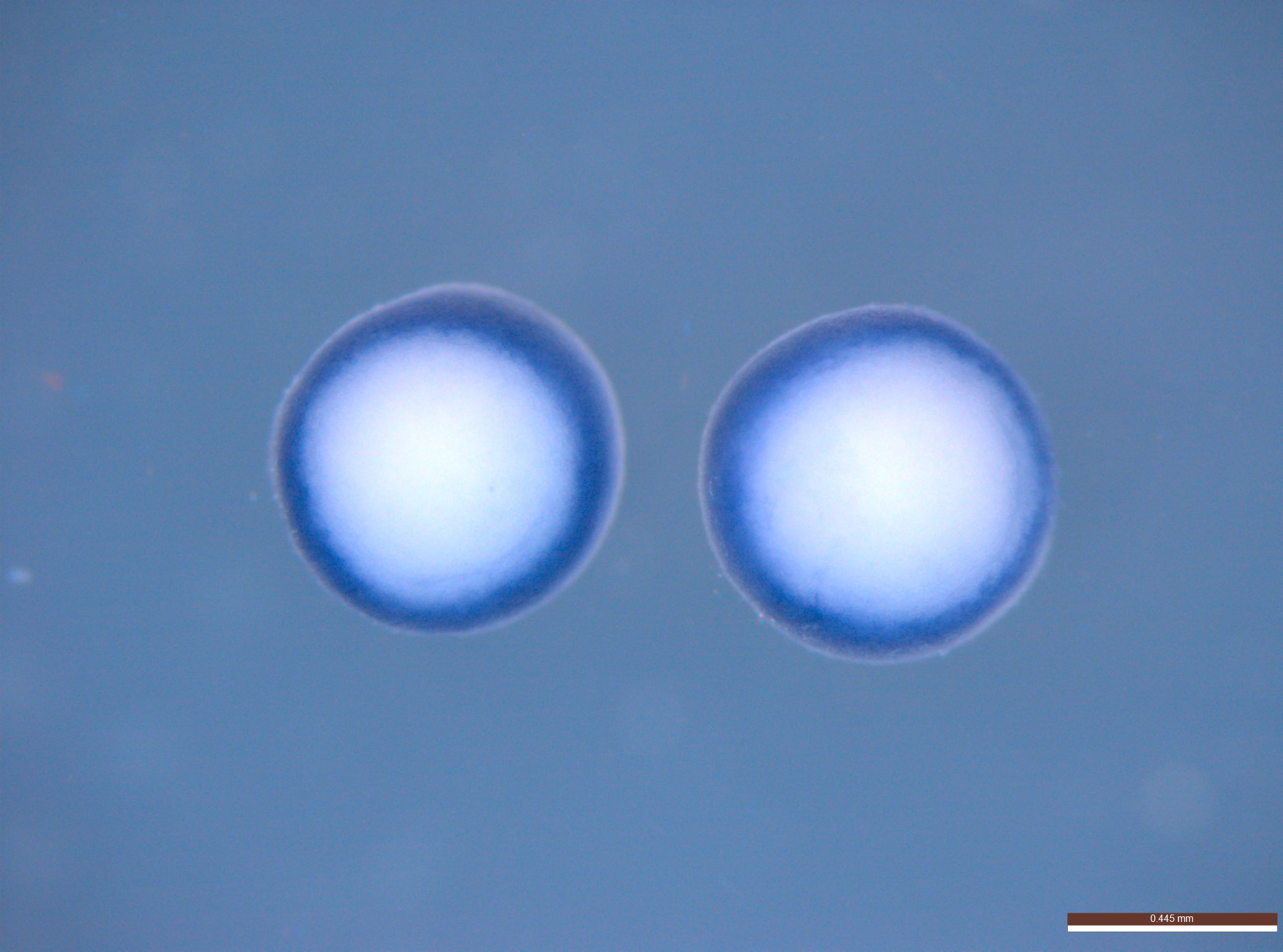

Supplement: Supplementary file 5 — Source Data for Figure 2 [file EMMM-15-e17078-s002.zip › Figure 2/2D_Images embryos/2D GoF images/24.tif]

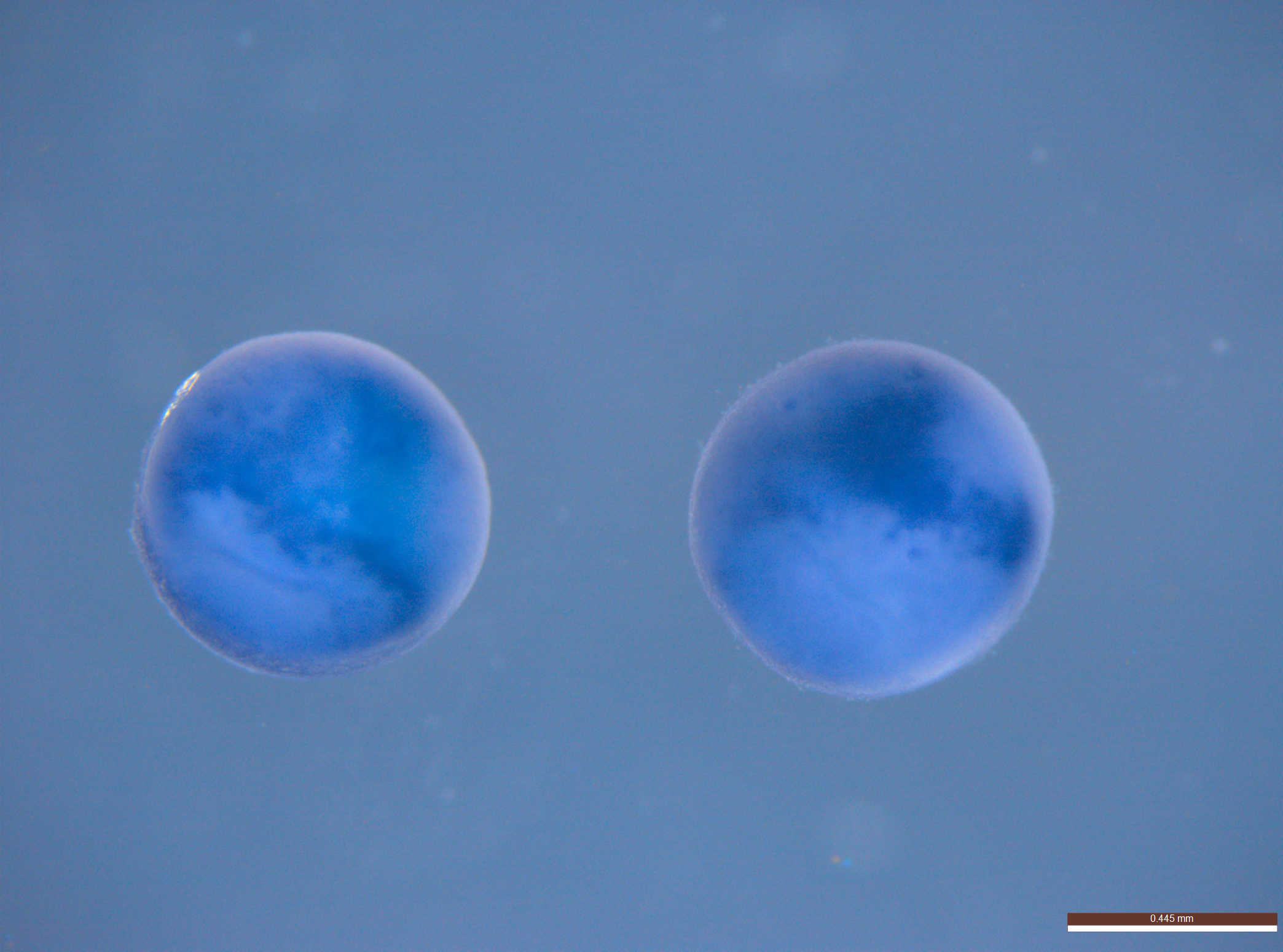

Supplement: Supplementary file 5 — Source Data for Figure 2 [file EMMM-15-e17078-s002.zip › Figure 2/2D_Images embryos/2D GoF images/25.tif]

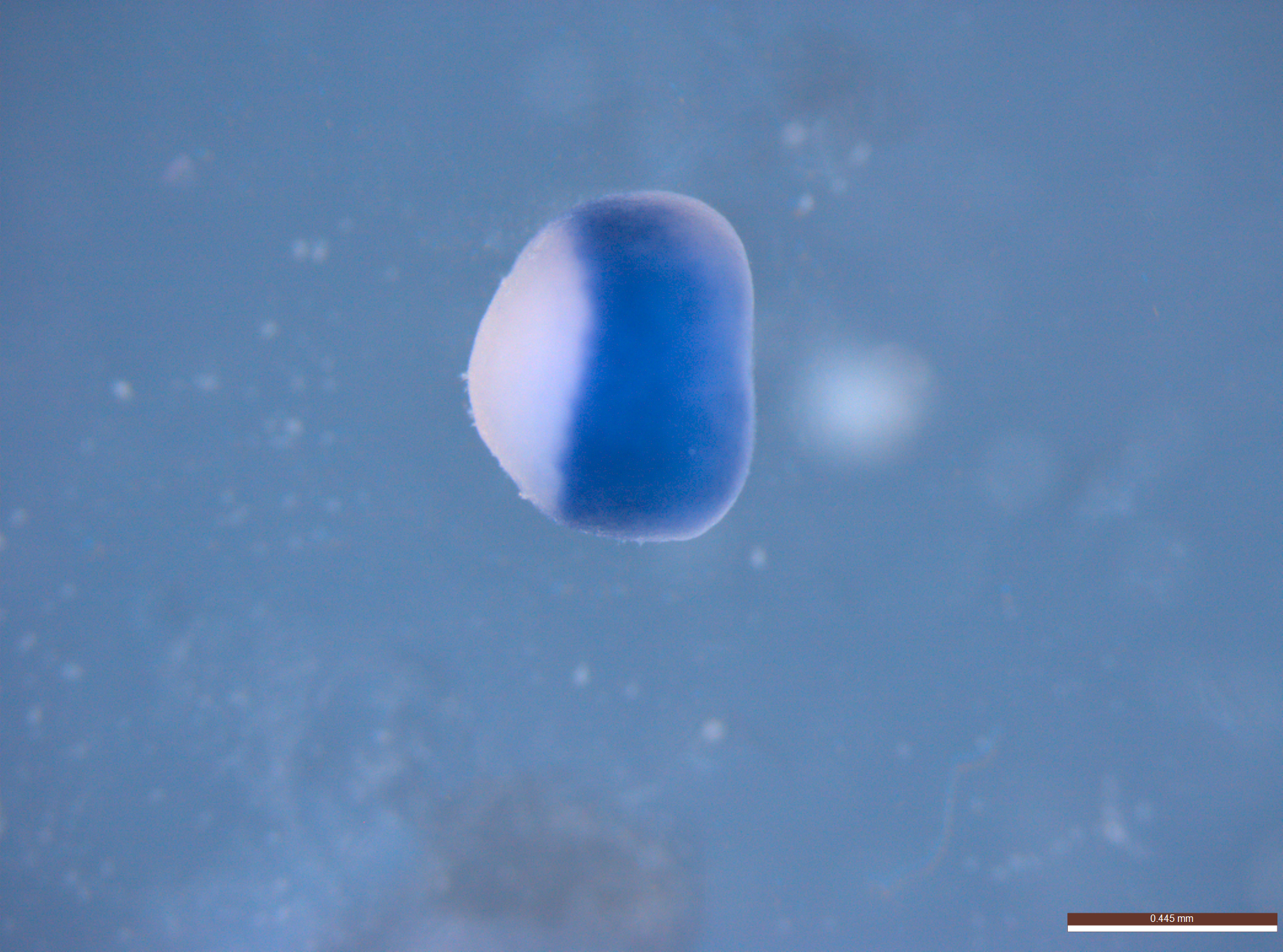

Supplement: Supplementary file 5 — Source Data for Figure 2 [file EMMM-15-e17078-s002.zip › Figure 2/2D_Images embryos/2D GoF images/26.tif]

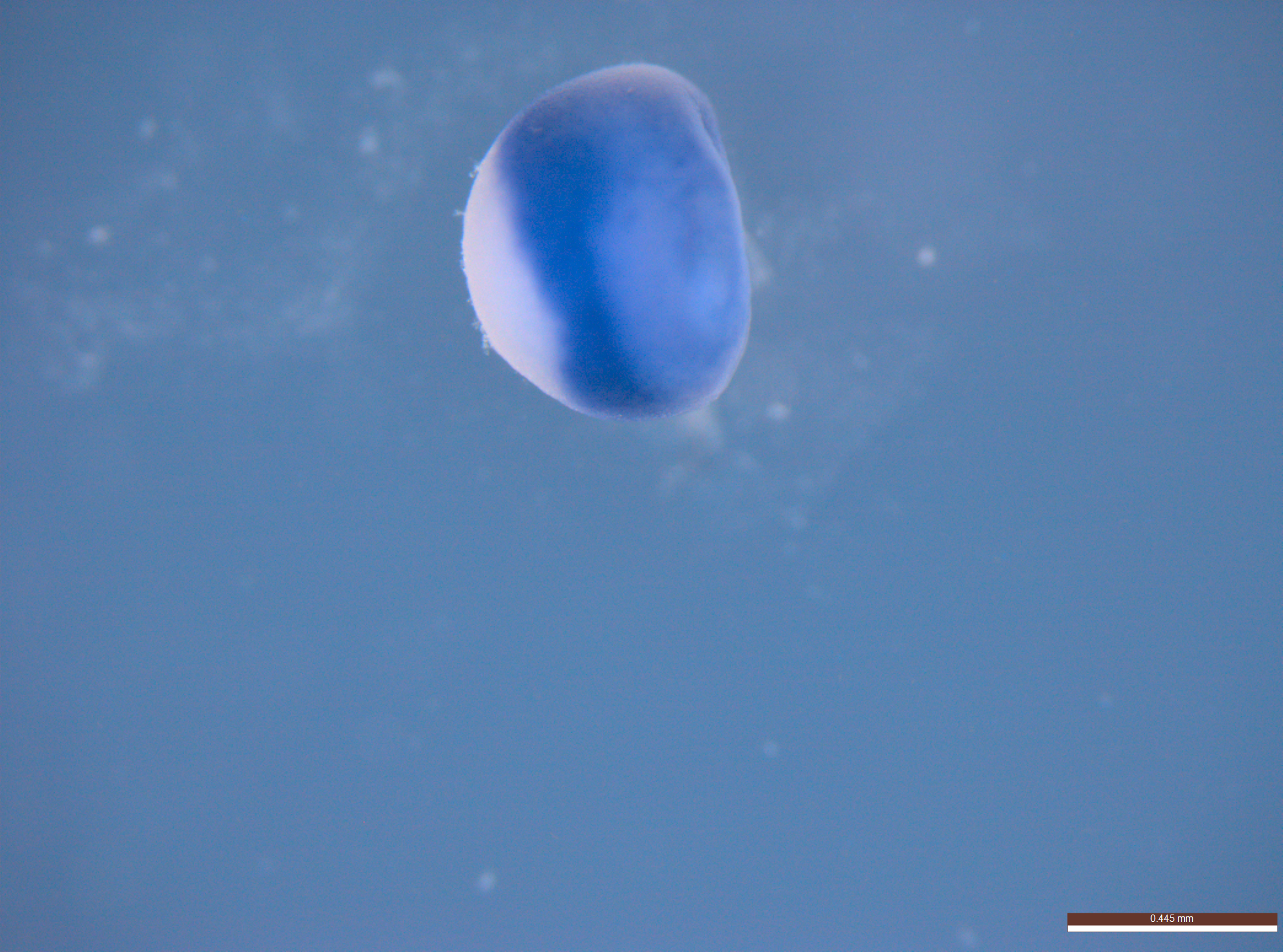

Supplement: Supplementary file 5 — Source Data for Figure 2 [file EMMM-15-e17078-s002.zip › Figure 2/2D_Images embryos/2D GoF images/28.tif]

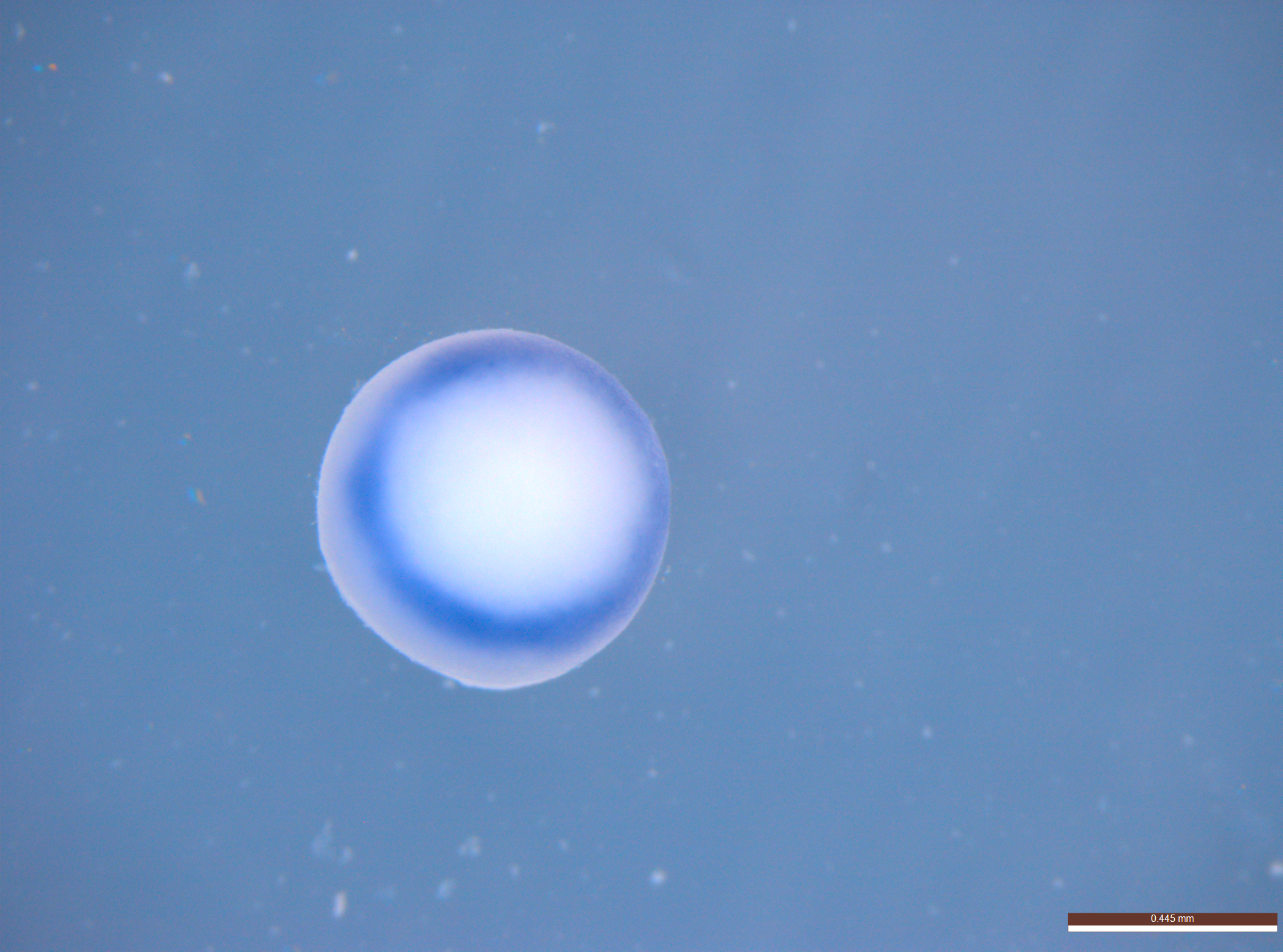

Supplement: Supplementary file 5 — Source Data for Figure 2 [file EMMM-15-e17078-s002.zip › Figure 2/2D_Images embryos/2D LoF images/39.tif]

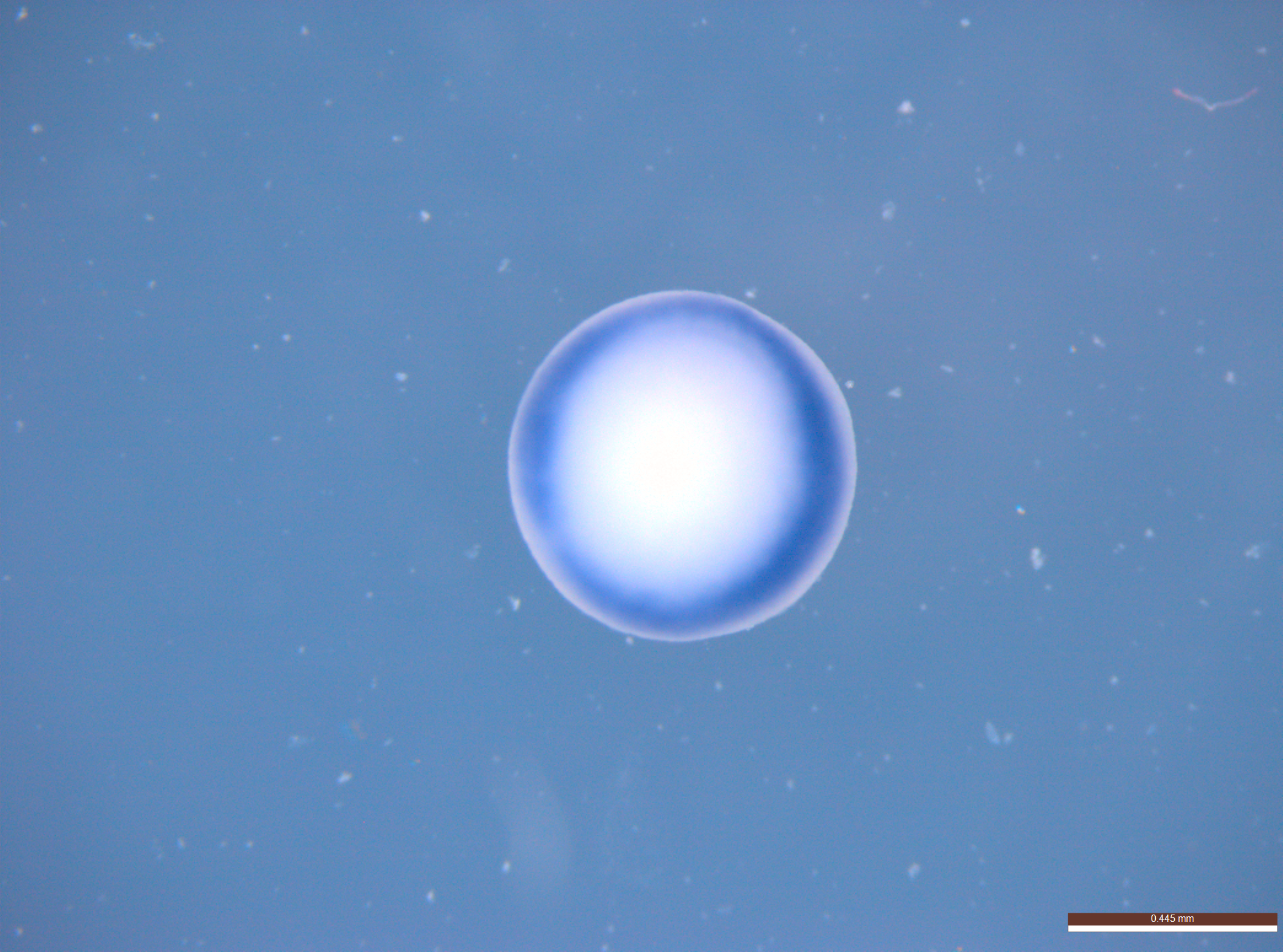

Supplement: Supplementary file 5 — Source Data for Figure 2 [file EMMM-15-e17078-s002.zip › Figure 2/2D_Images embryos/2D LoF images/40.tif]

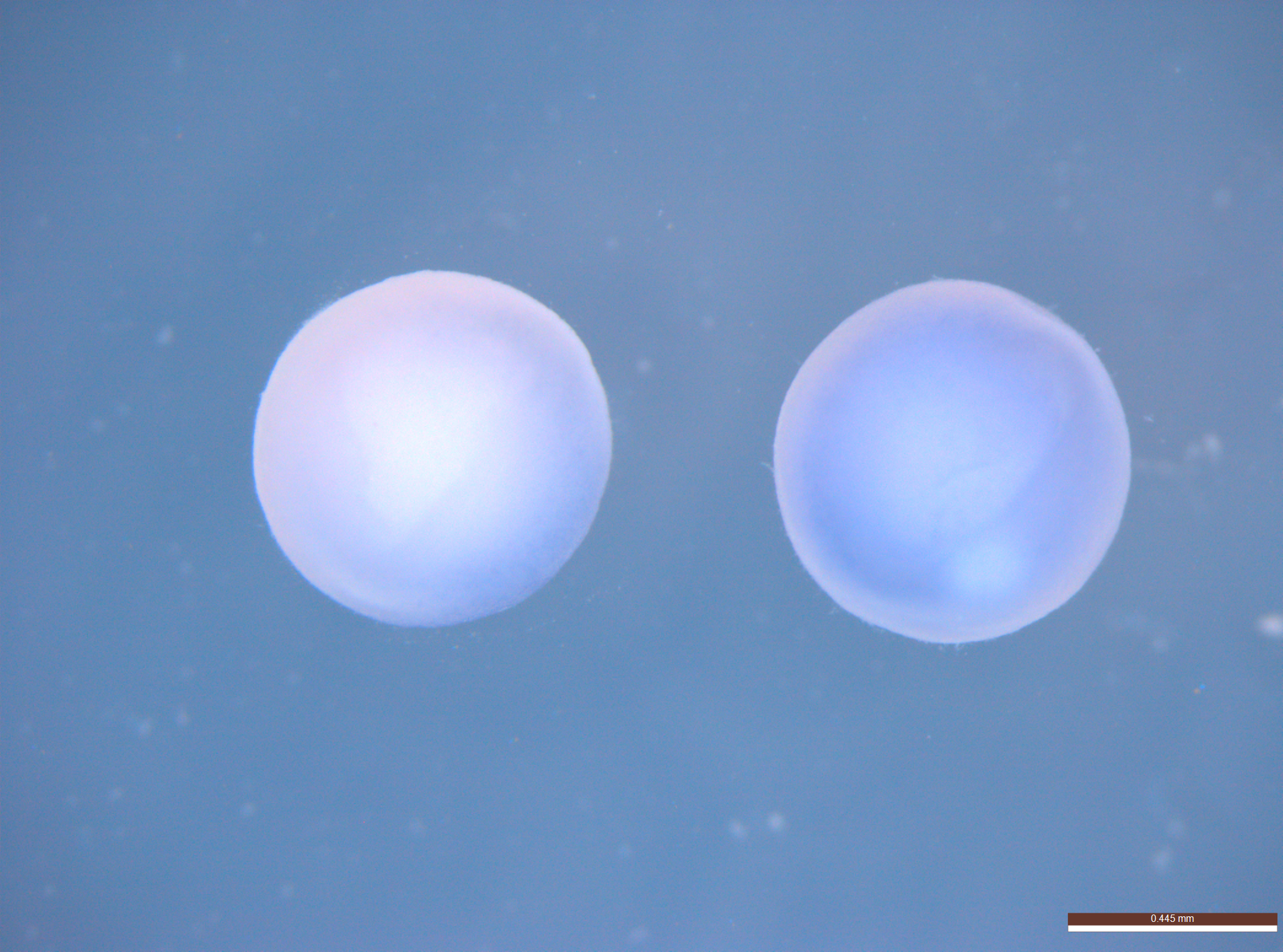

Supplement: Supplementary file 5 — Source Data for Figure 2 [file EMMM-15-e17078-s002.zip › Figure 2/2D_Images embryos/2D LoF images/42.tif]

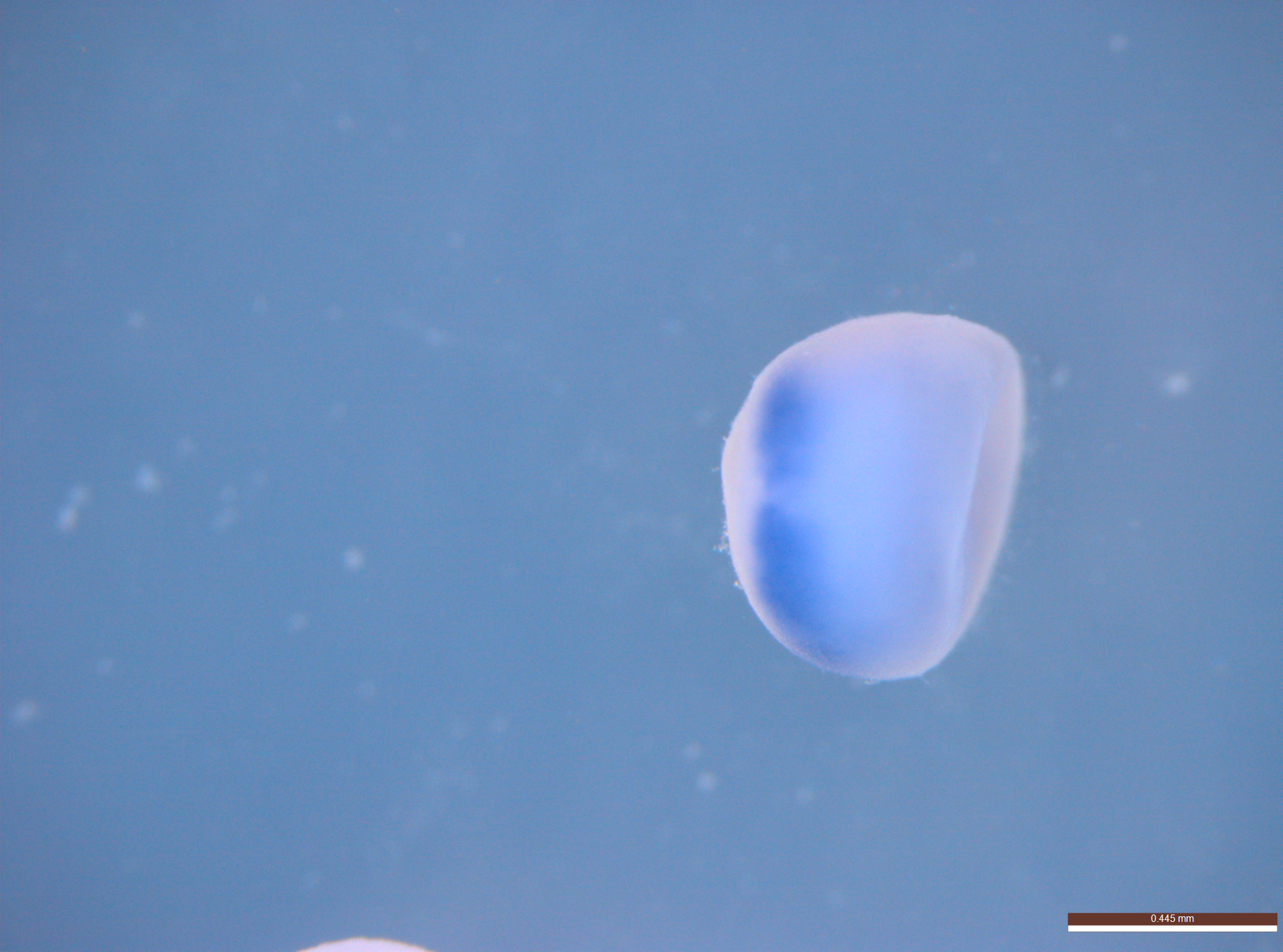

Supplement: Supplementary file 5 — Source Data for Figure 2 [file EMMM-15-e17078-s002.zip › Figure 2/2D_Images embryos/2D LoF images/43.tif]

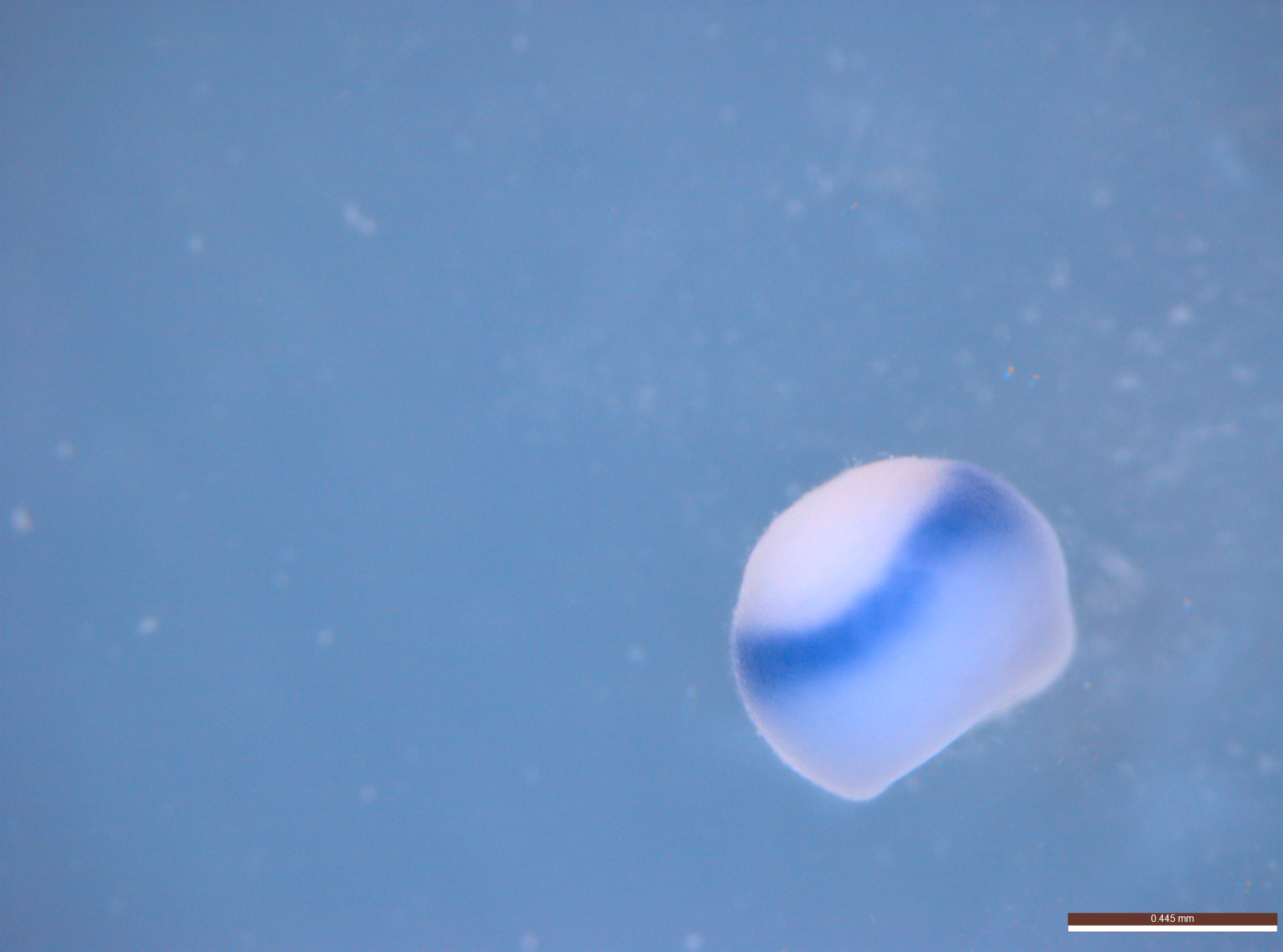

Supplement: Supplementary file 5 — Source Data for Figure 2 [file EMMM-15-e17078-s002.zip › Figure 2/2D_Images embryos/2D LoF images/45.tif]

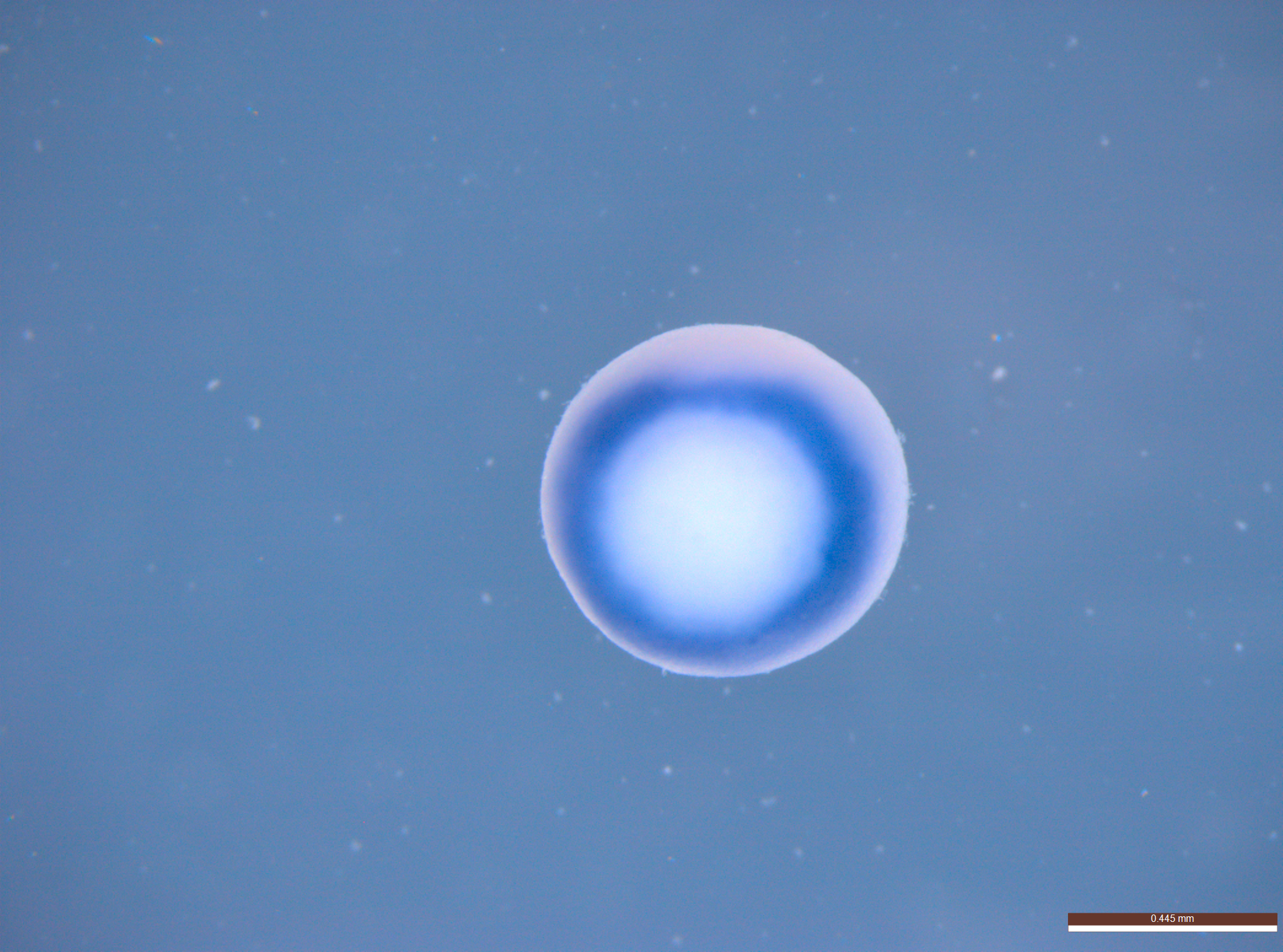

Supplement: Supplementary file 5 — Source Data for Figure 2 [file EMMM-15-e17078-s002.zip › Figure 2/2D_Images embryos/2D T543M images/34.tif]

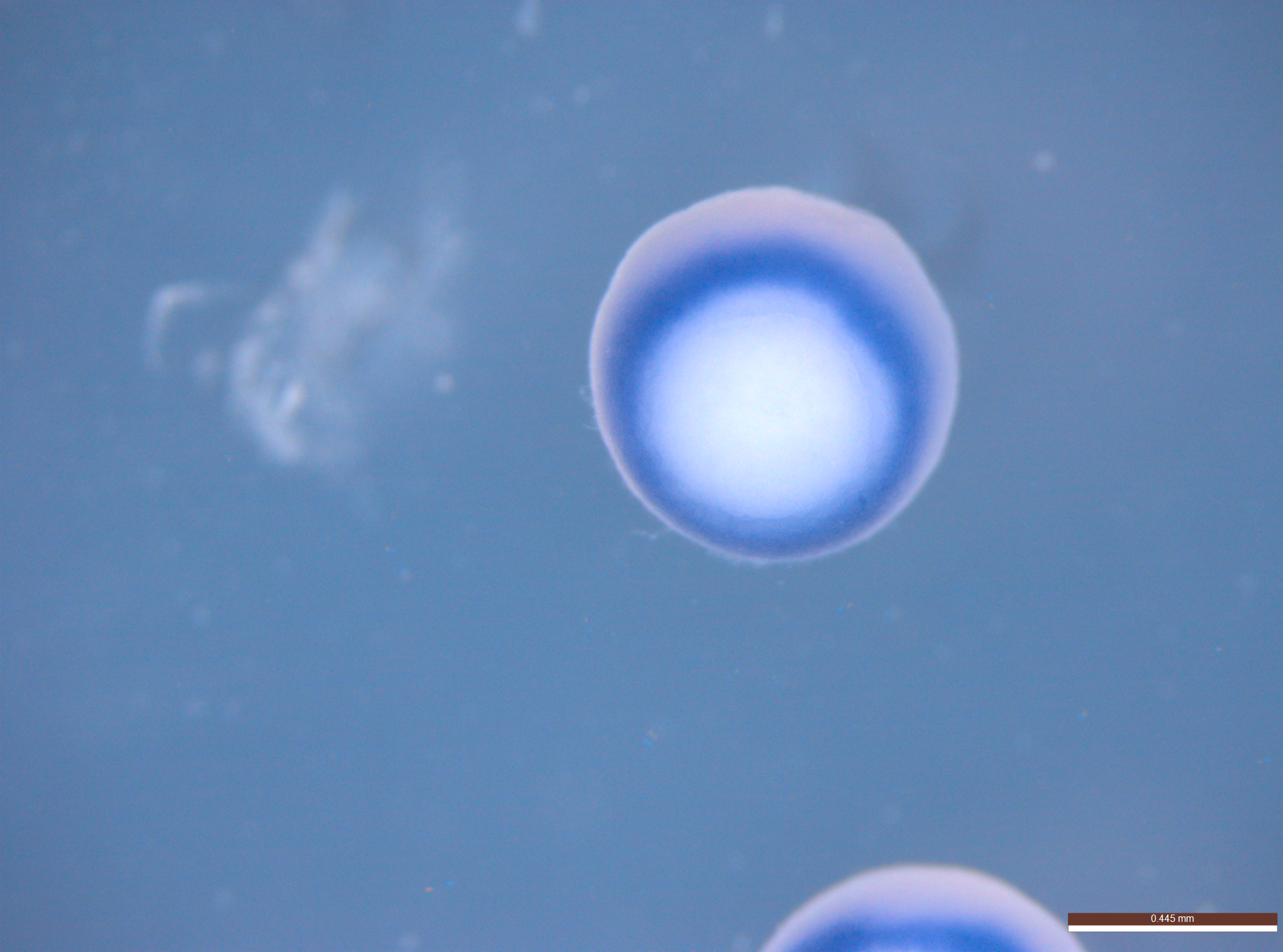

Supplement: Supplementary file 5 — Source Data for Figure 2 [file EMMM-15-e17078-s002.zip › Figure 2/2D_Images embryos/2D T543M images/35.tif]

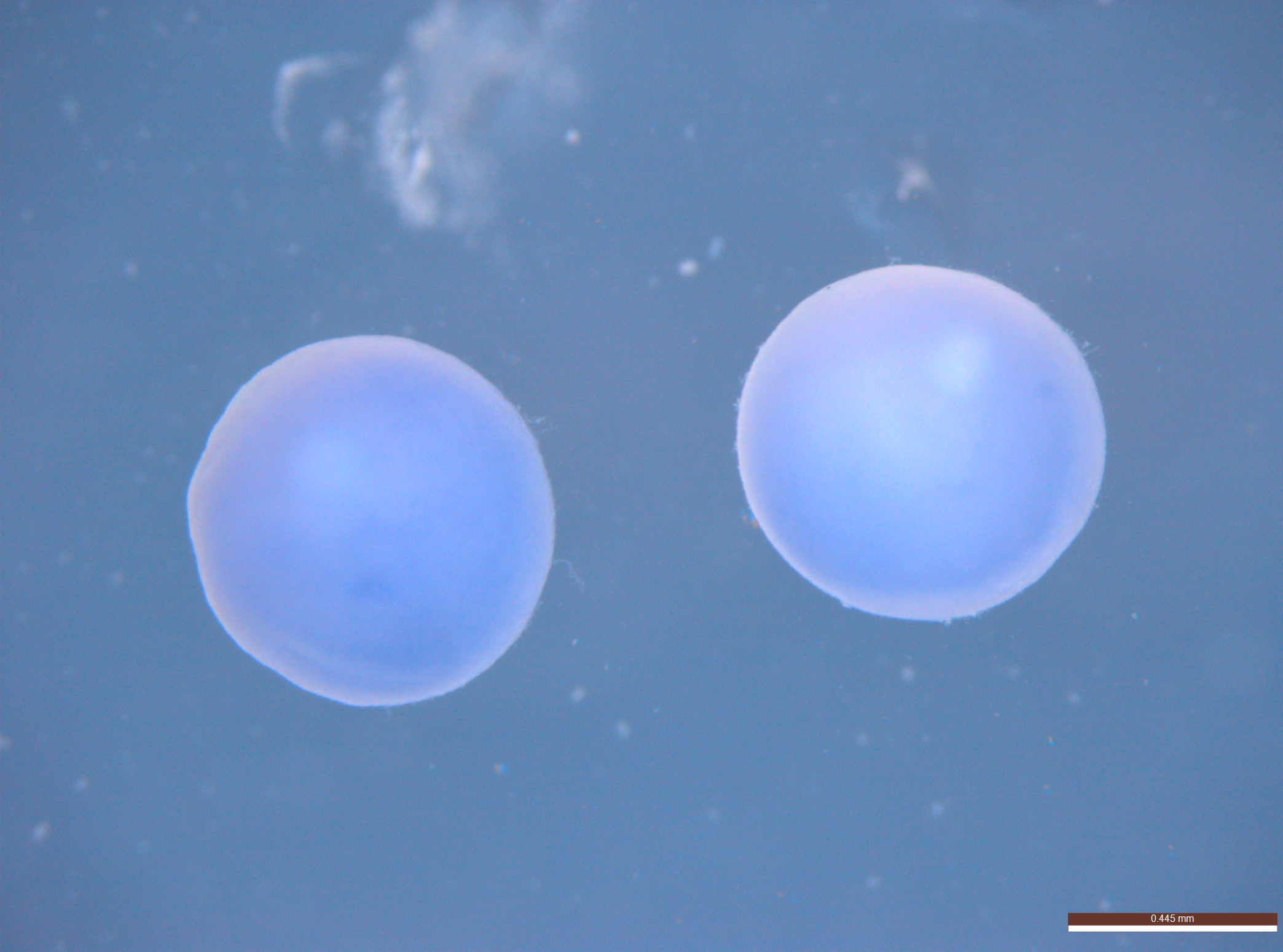

Supplement: Supplementary file 5 — Source Data for Figure 2 [file EMMM-15-e17078-s002.zip › Figure 2/2D_Images embryos/2D T543M images/36.tif]

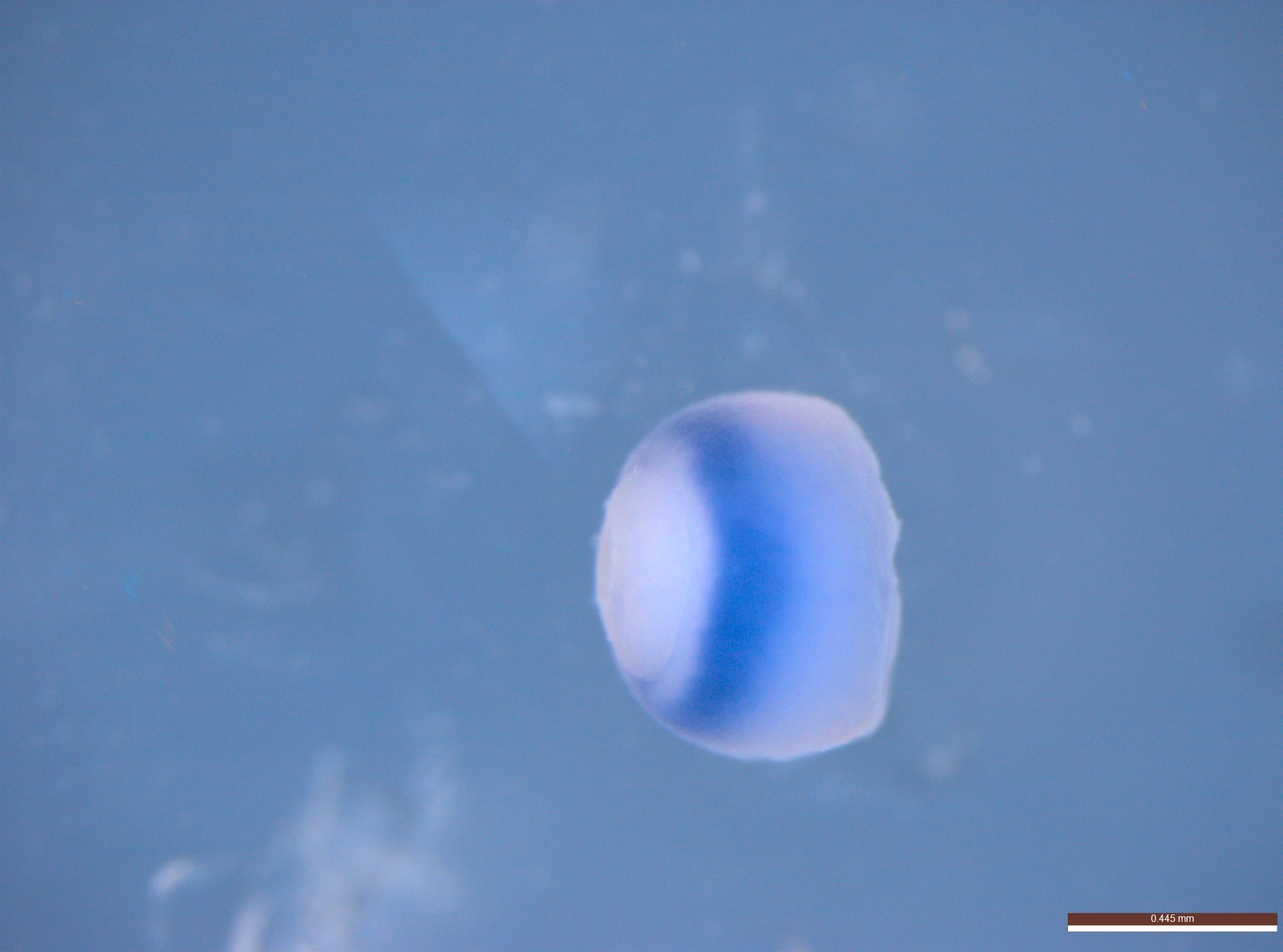

Supplement: Supplementary file 5 — Source Data for Figure 2 [file EMMM-15-e17078-s002.zip › Figure 2/2D_Images embryos/2D T543M images/37.tif]

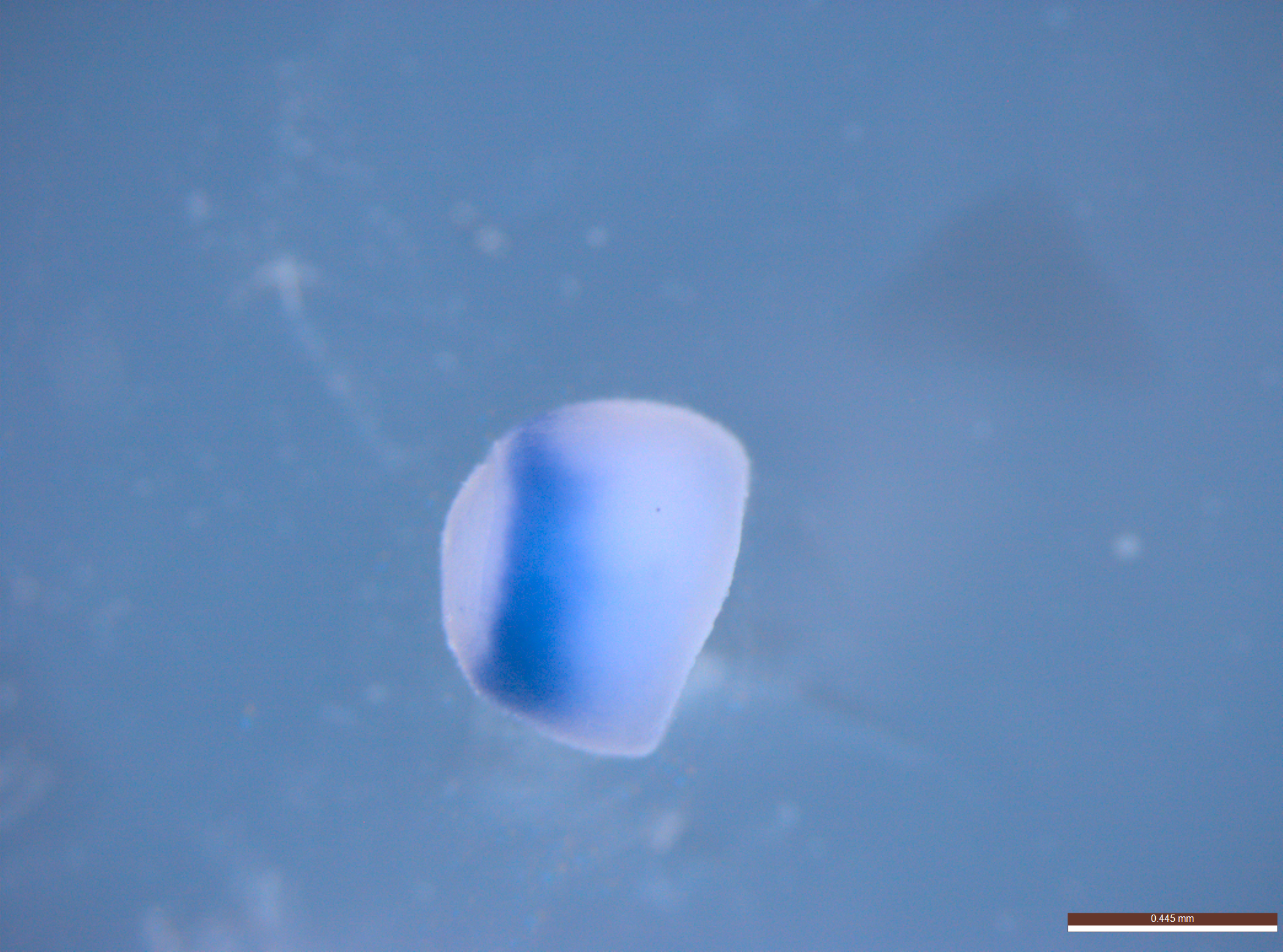

Supplement: Supplementary file 5 — Source Data for Figure 2 [file EMMM-15-e17078-s002.zip › Figure 2/2D_Images embryos/2D T543M images/38.tif]

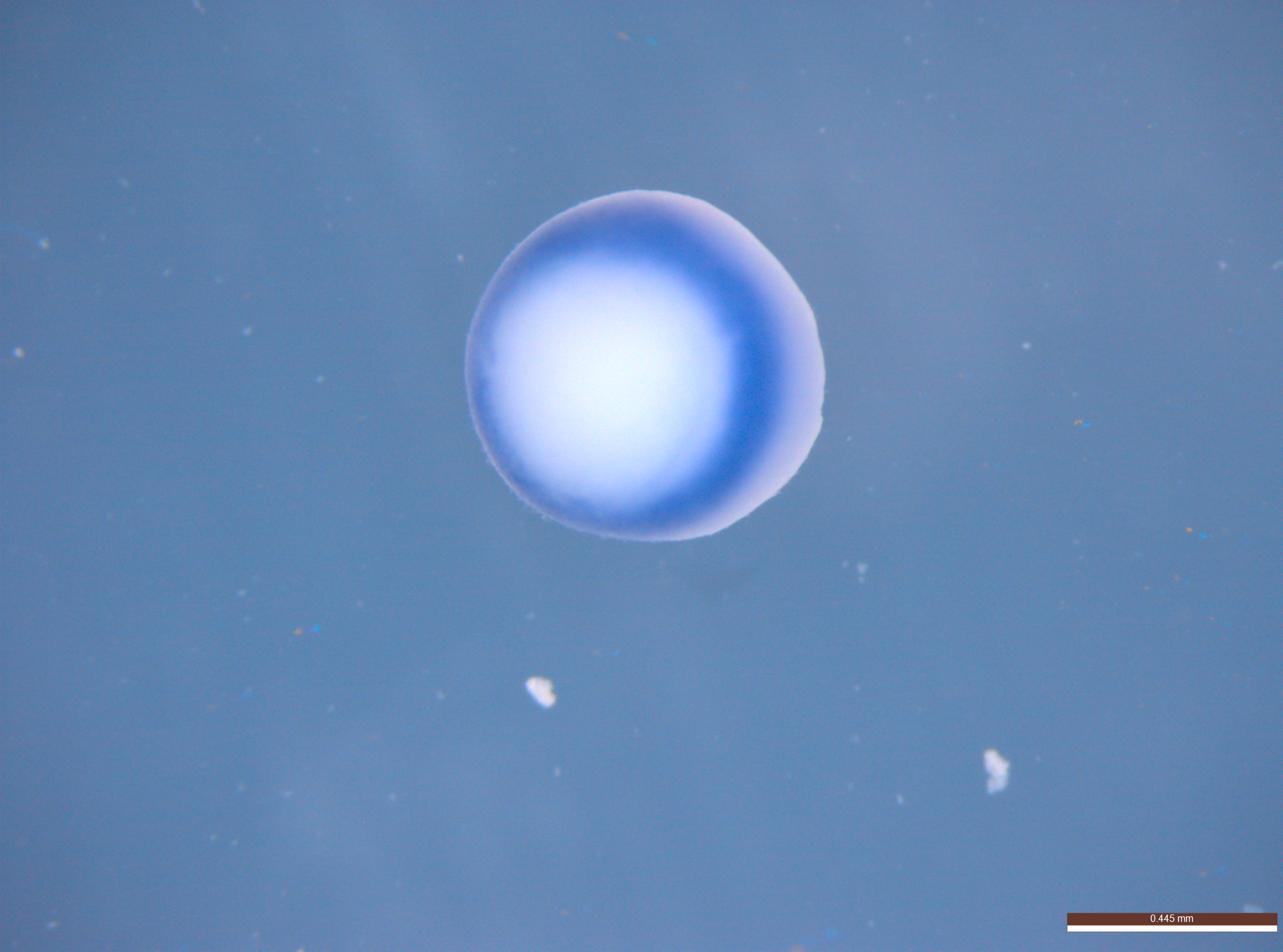

Supplement: Supplementary file 5 — Source Data for Figure 2 [file EMMM-15-e17078-s002.zip › Figure 2/2D_Images embryos/2D Uninjected images/29.tif]

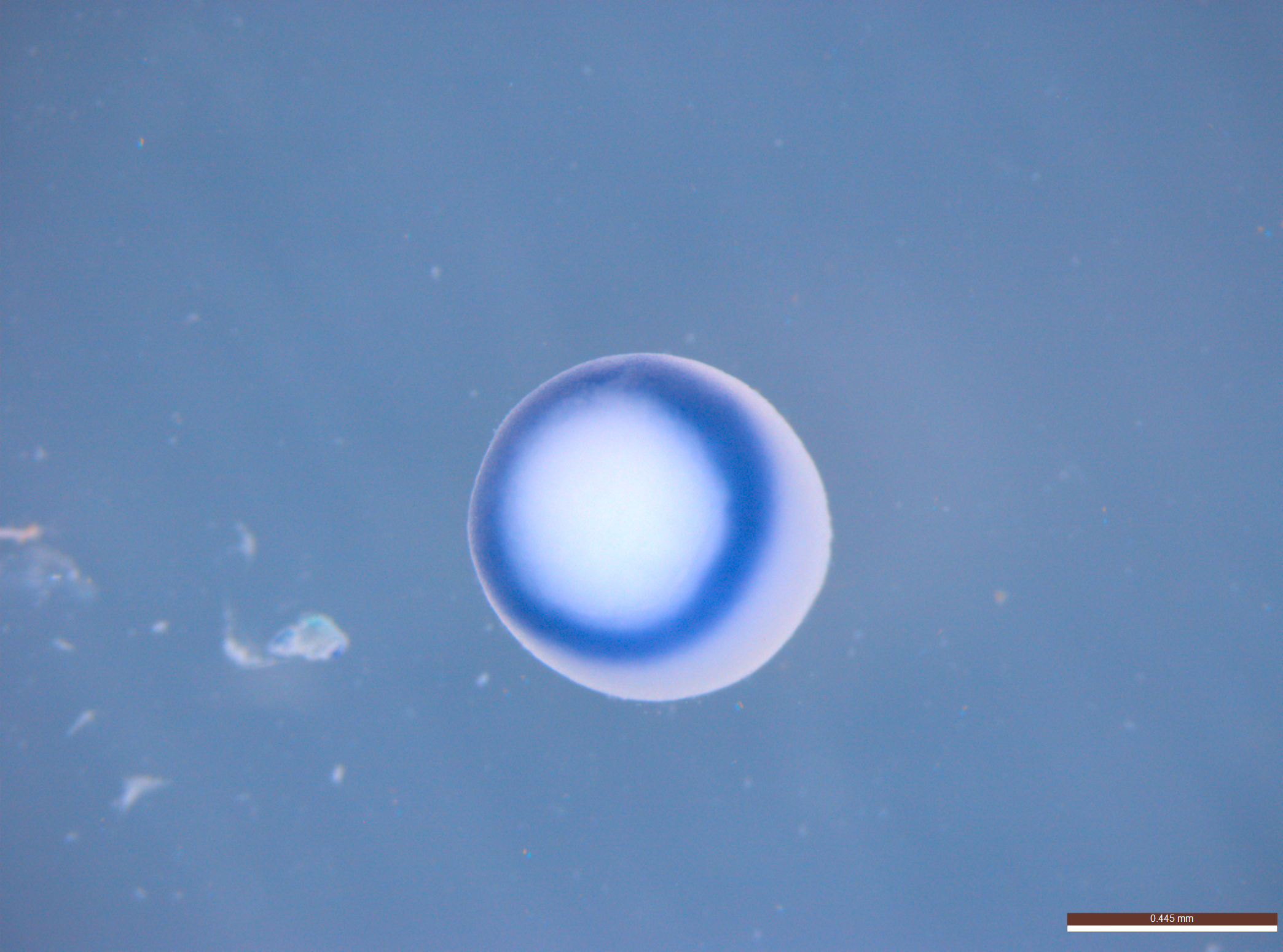

Supplement: Supplementary file 5 — Source Data for Figure 2 [file EMMM-15-e17078-s002.zip › Figure 2/2D_Images embryos/2D Uninjected images/30.tif]

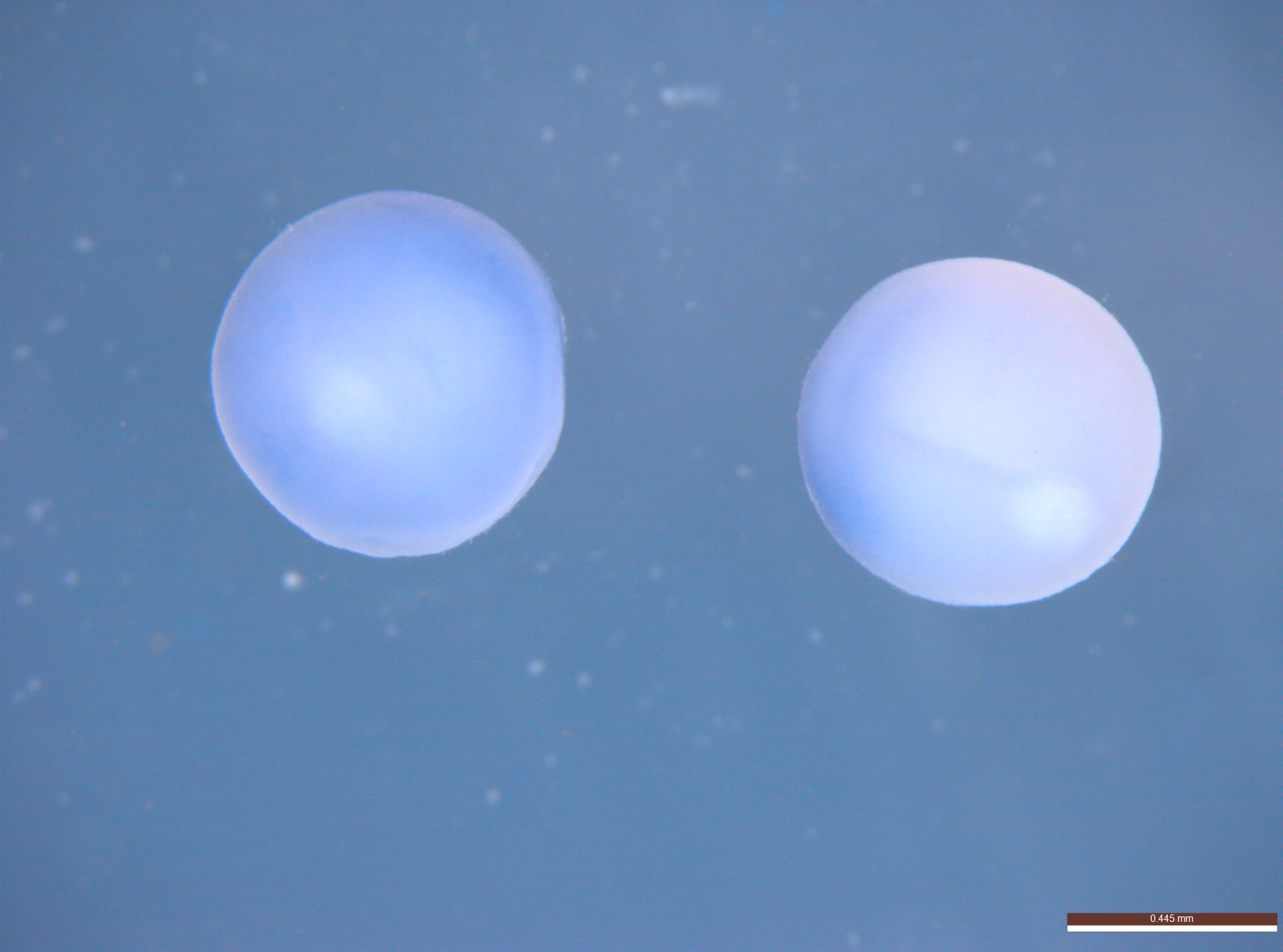

Supplement: Supplementary file 5 — Source Data for Figure 2 [file EMMM-15-e17078-s002.zip › Figure 2/2D_Images embryos/2D Uninjected images/31.tif]

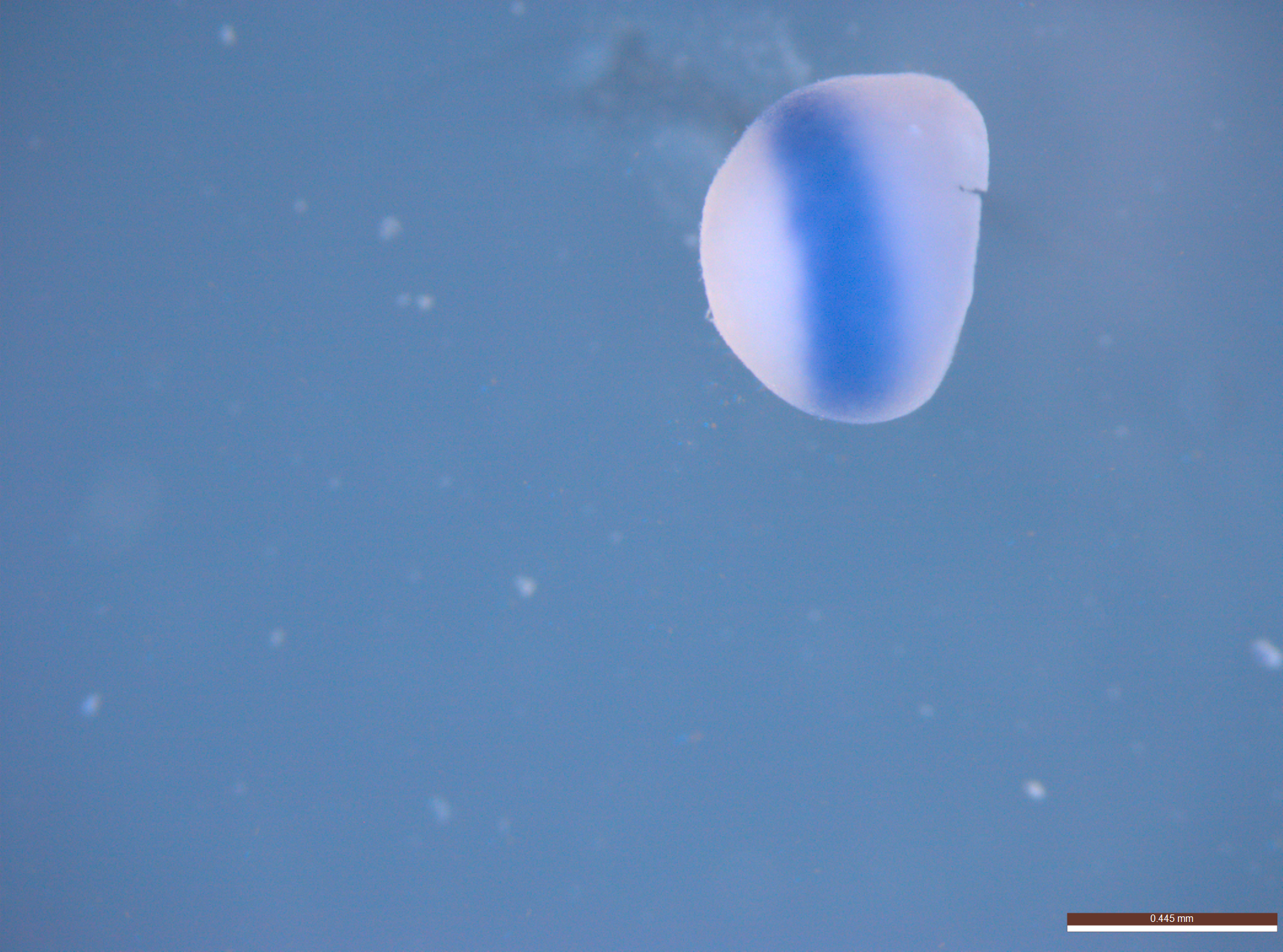

Supplement: Supplementary file 5 — Source Data for Figure 2 [file EMMM-15-e17078-s002.zip › Figure 2/2D_Images embryos/2D Uninjected images/32.tif]

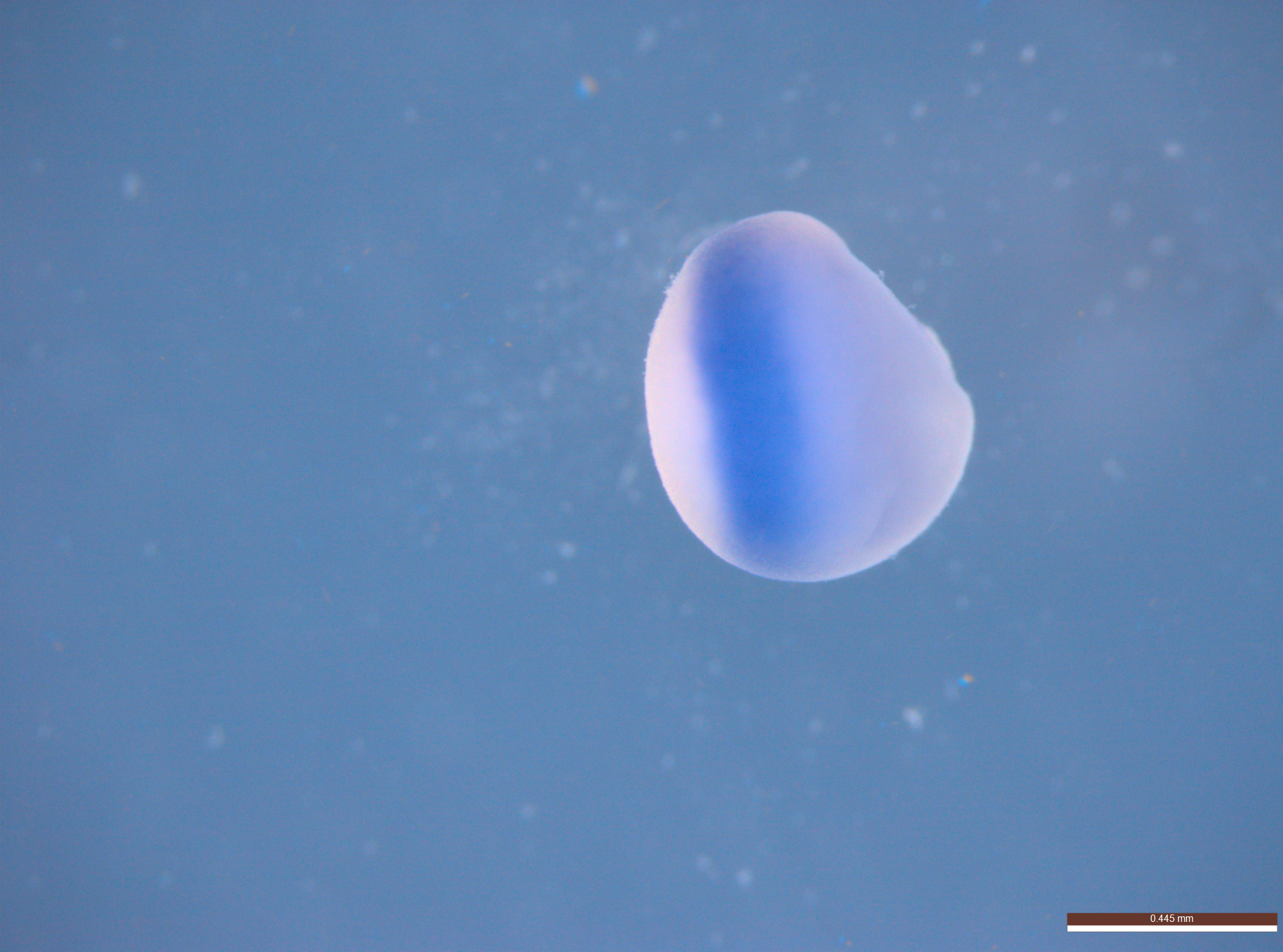

Supplement: Supplementary file 5 — Source Data for Figure 2 [file EMMM-15-e17078-s002.zip › Figure 2/2D_Images embryos/2D Uninjected images/33.tif]

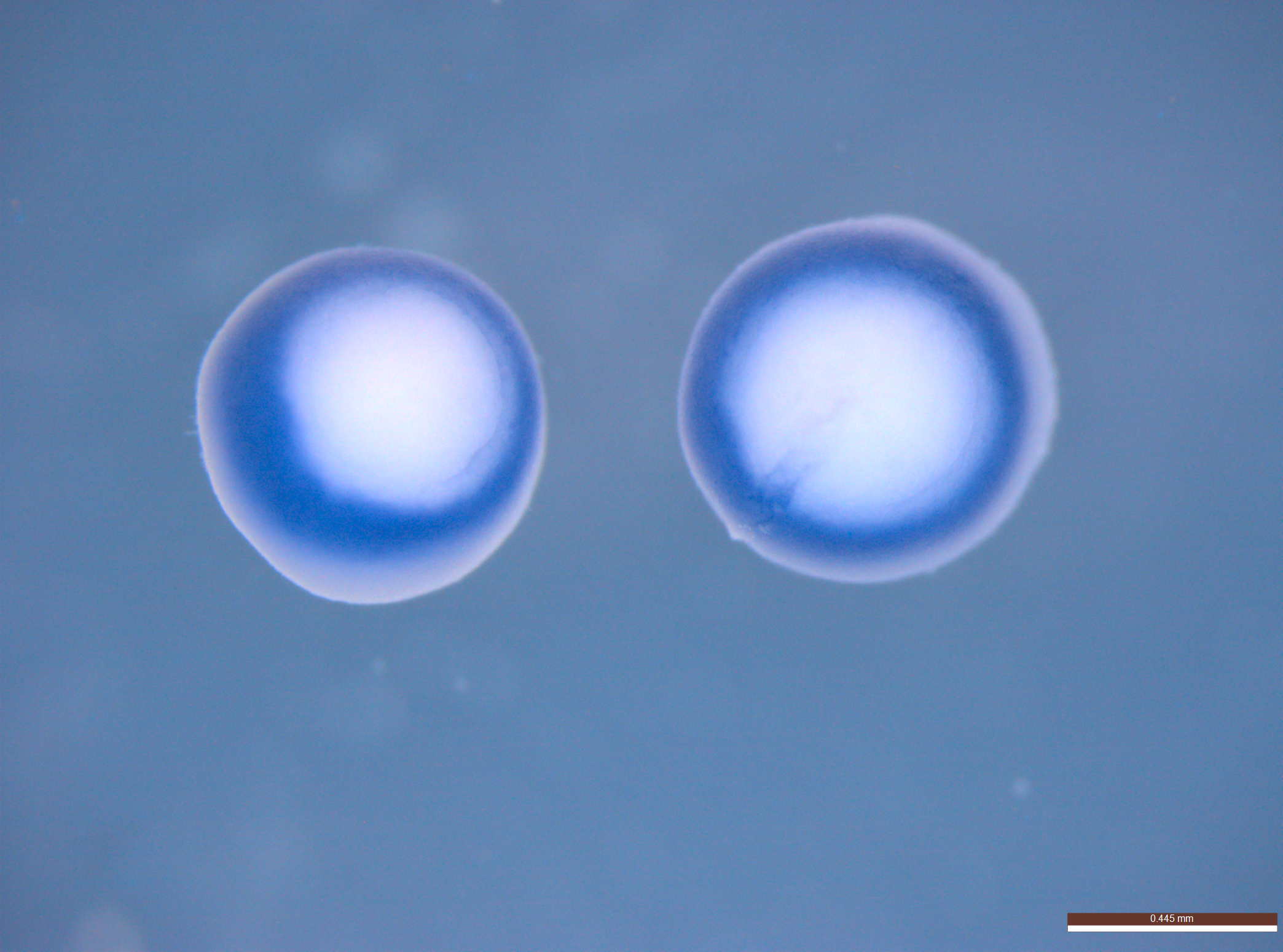

Supplement: Supplementary file 5 — Source Data for Figure 2 [file EMMM-15-e17078-s002.zip › Figure 2/2D_Images embryos/2D WT images/19.tif]

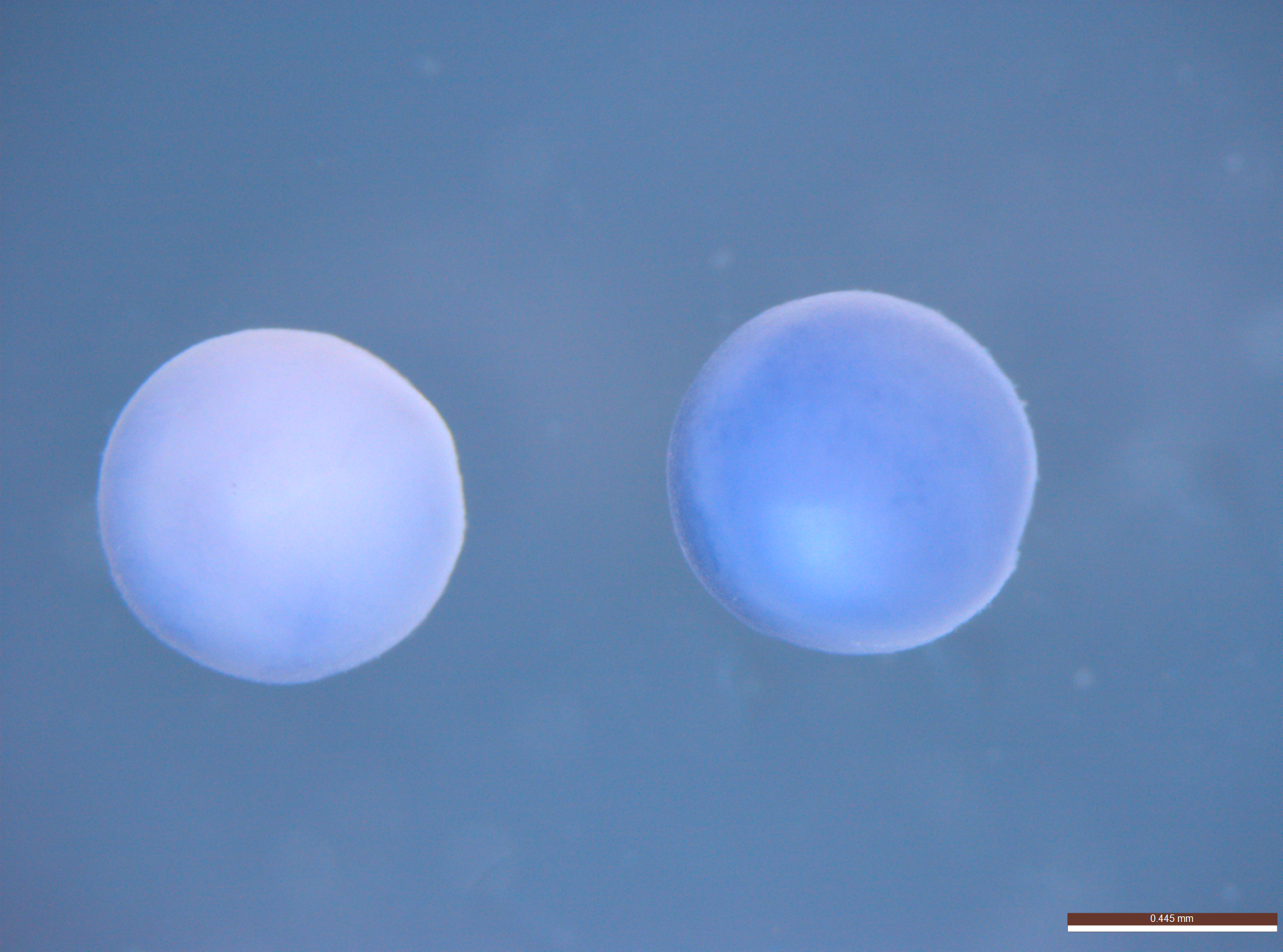

Supplement: Supplementary file 5 — Source Data for Figure 2 [file EMMM-15-e17078-s002.zip › Figure 2/2D_Images embryos/2D WT images/20.tif]

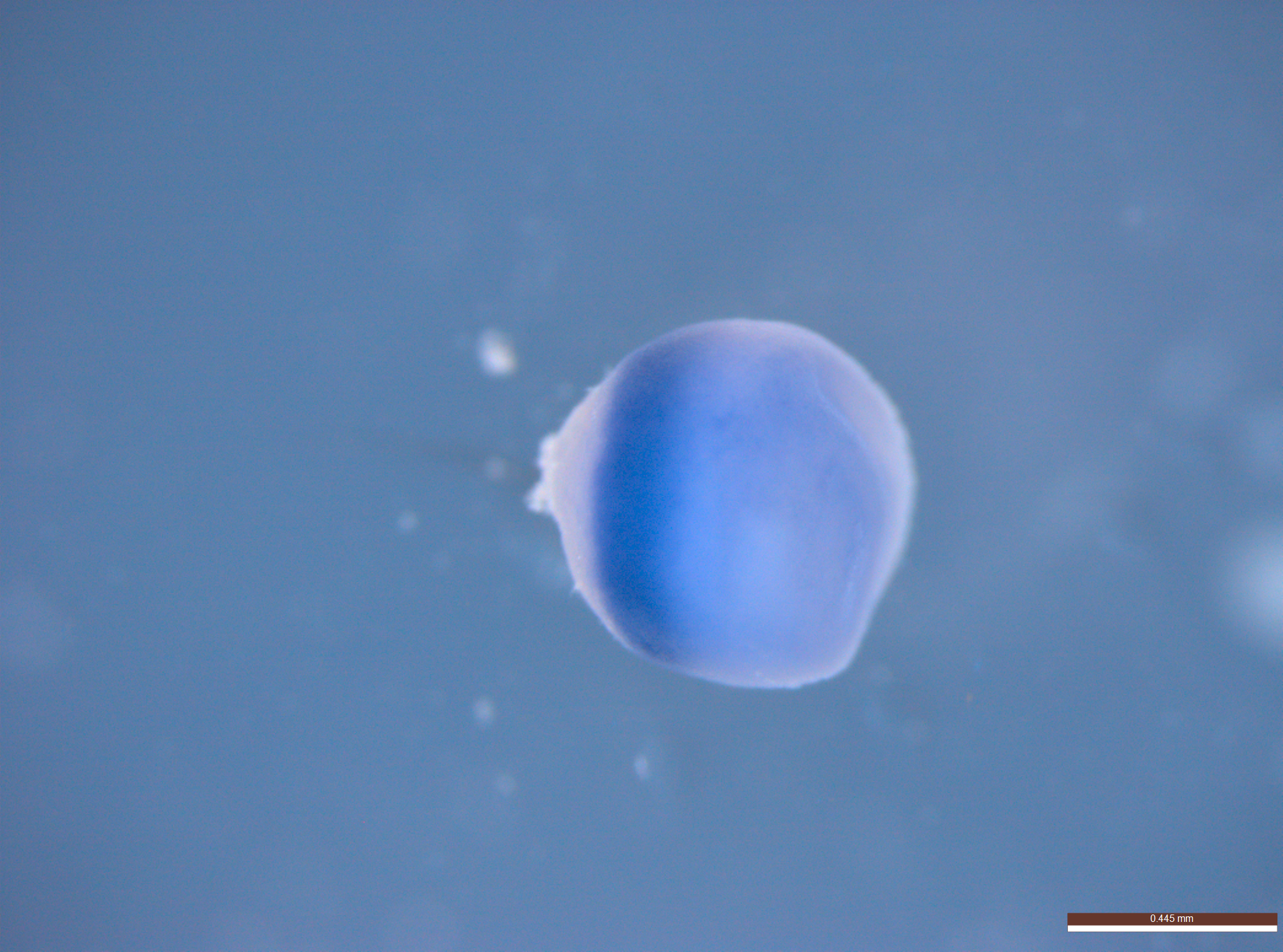

Supplement: Supplementary file 5 — Source Data for Figure 2 [file EMMM-15-e17078-s002.zip › Figure 2/2D_Images embryos/2D WT images/21.tif]

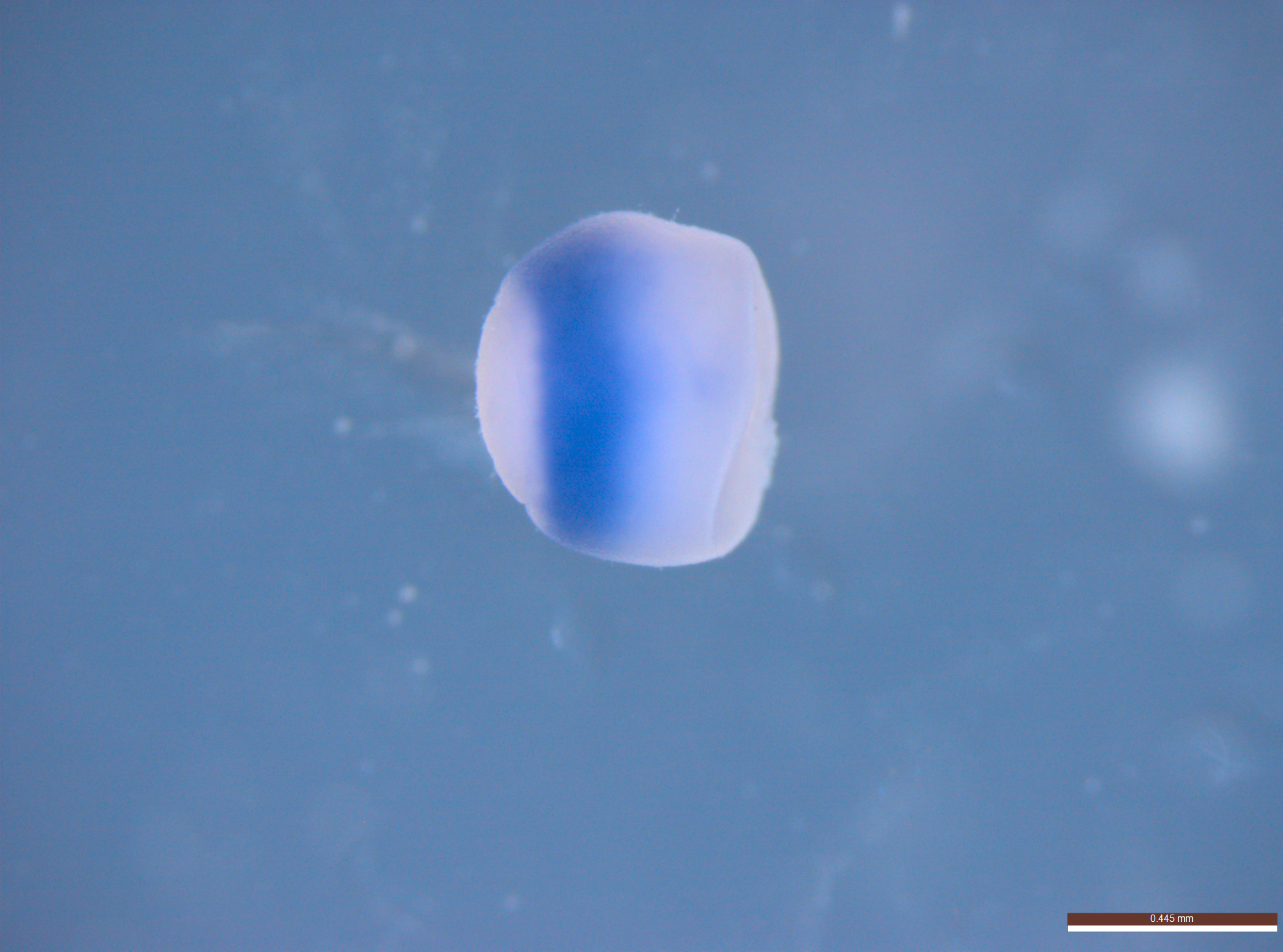

Supplement: Supplementary file 5 — Source Data for Figure 2 [file EMMM-15-e17078-s002.zip › Figure 2/2D_Images embryos/2D WT images/22.tif]

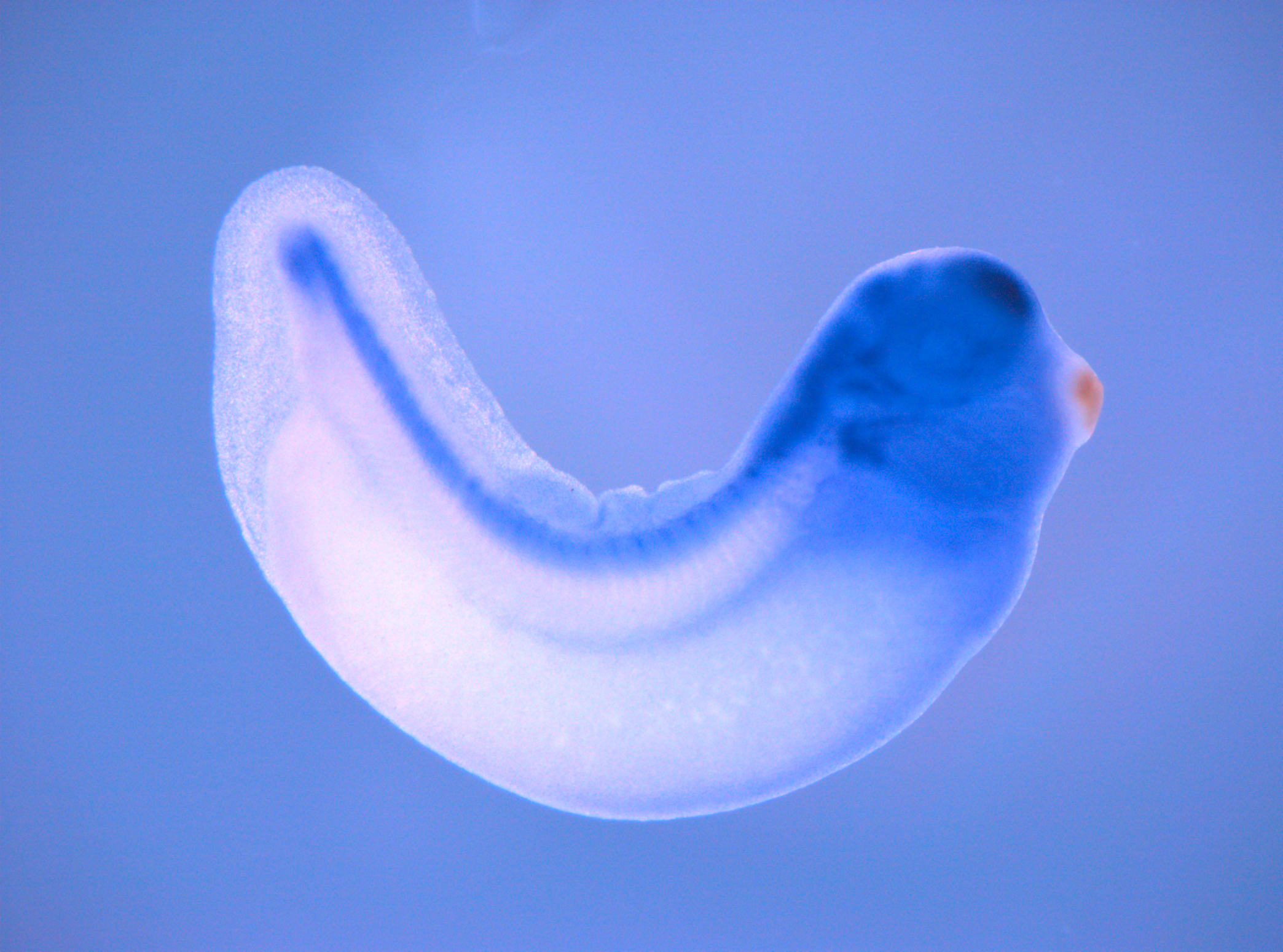

Supplement: Supplementary file 5 — Source Data for Figure 2 [file EMMM-15-e17078-s002.zip › Figure 2/2E_images embryos/N-Tubulin/image0027.tif]

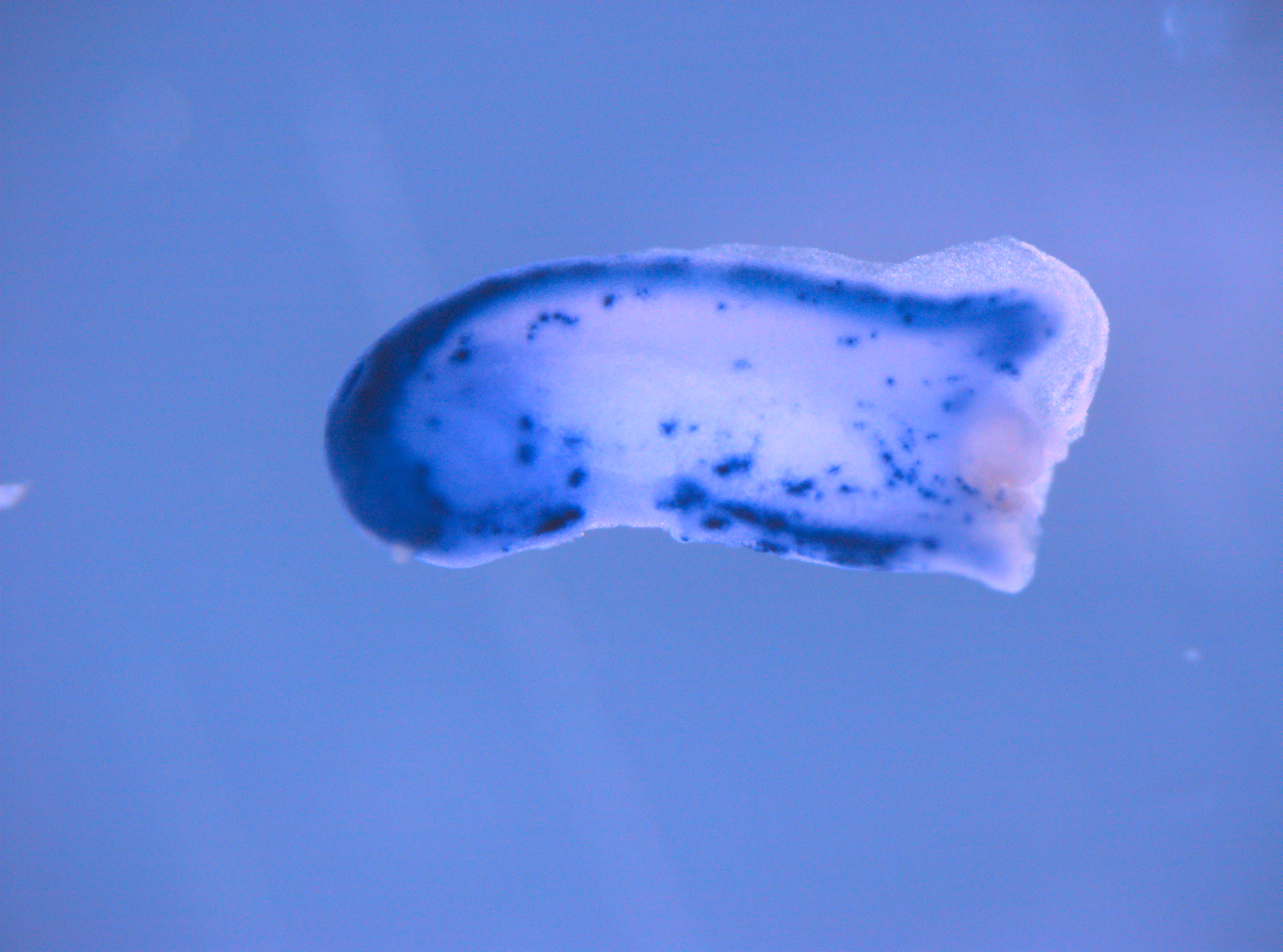

Supplement: Supplementary file 5 — Source Data for Figure 2 [file EMMM-15-e17078-s002.zip › Figure 2/2E_images embryos/N-Tubulin/image0030.tif]

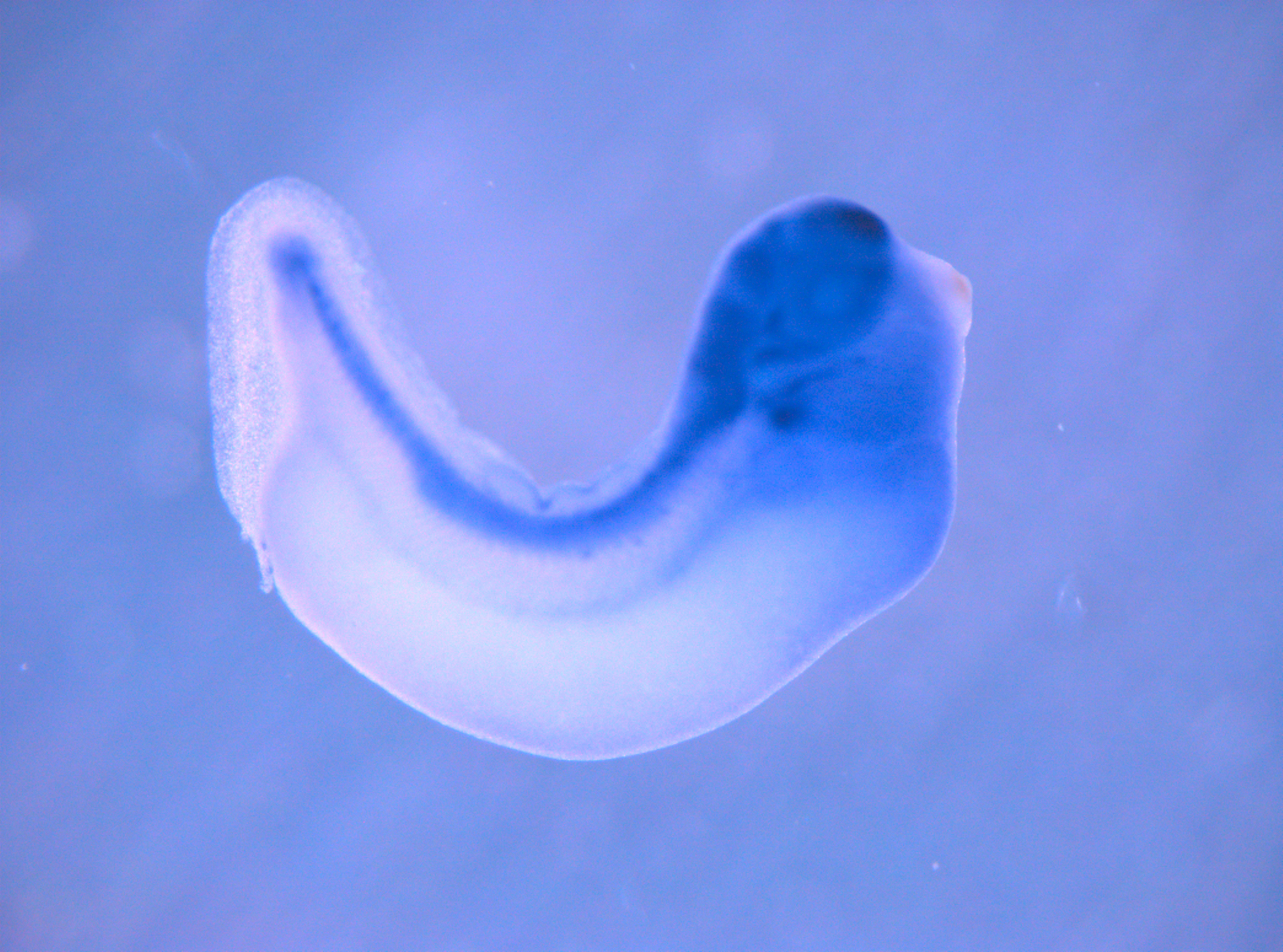

Supplement: Supplementary file 5 — Source Data for Figure 2 [file EMMM-15-e17078-s002.zip › Figure 2/2E_images embryos/N-Tubulin/image0035.tif]

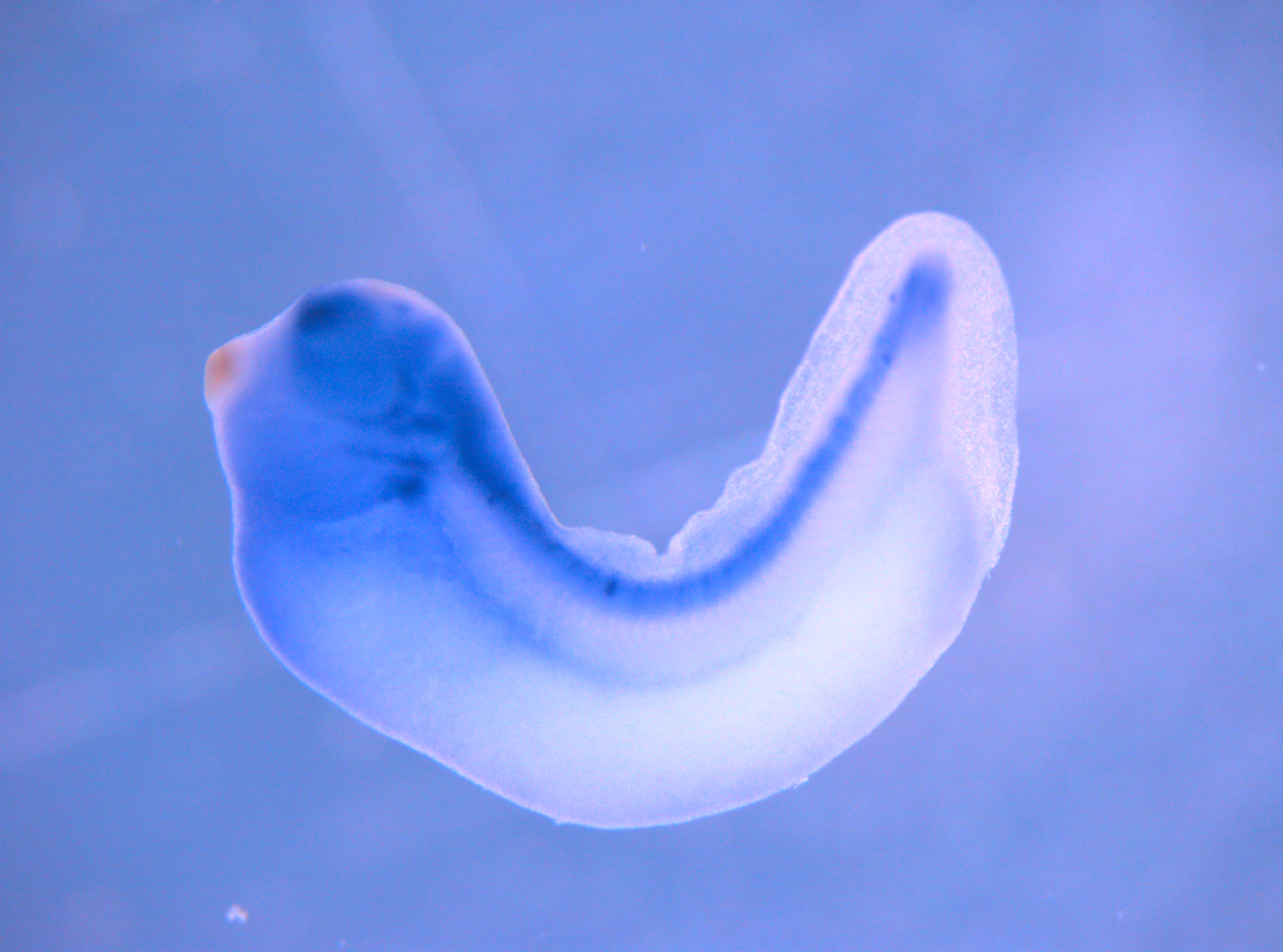

Supplement: Supplementary file 5 — Source Data for Figure 2 [file EMMM-15-e17078-s002.zip › Figure 2/2E_images embryos/N-Tubulin/image0037.tif]

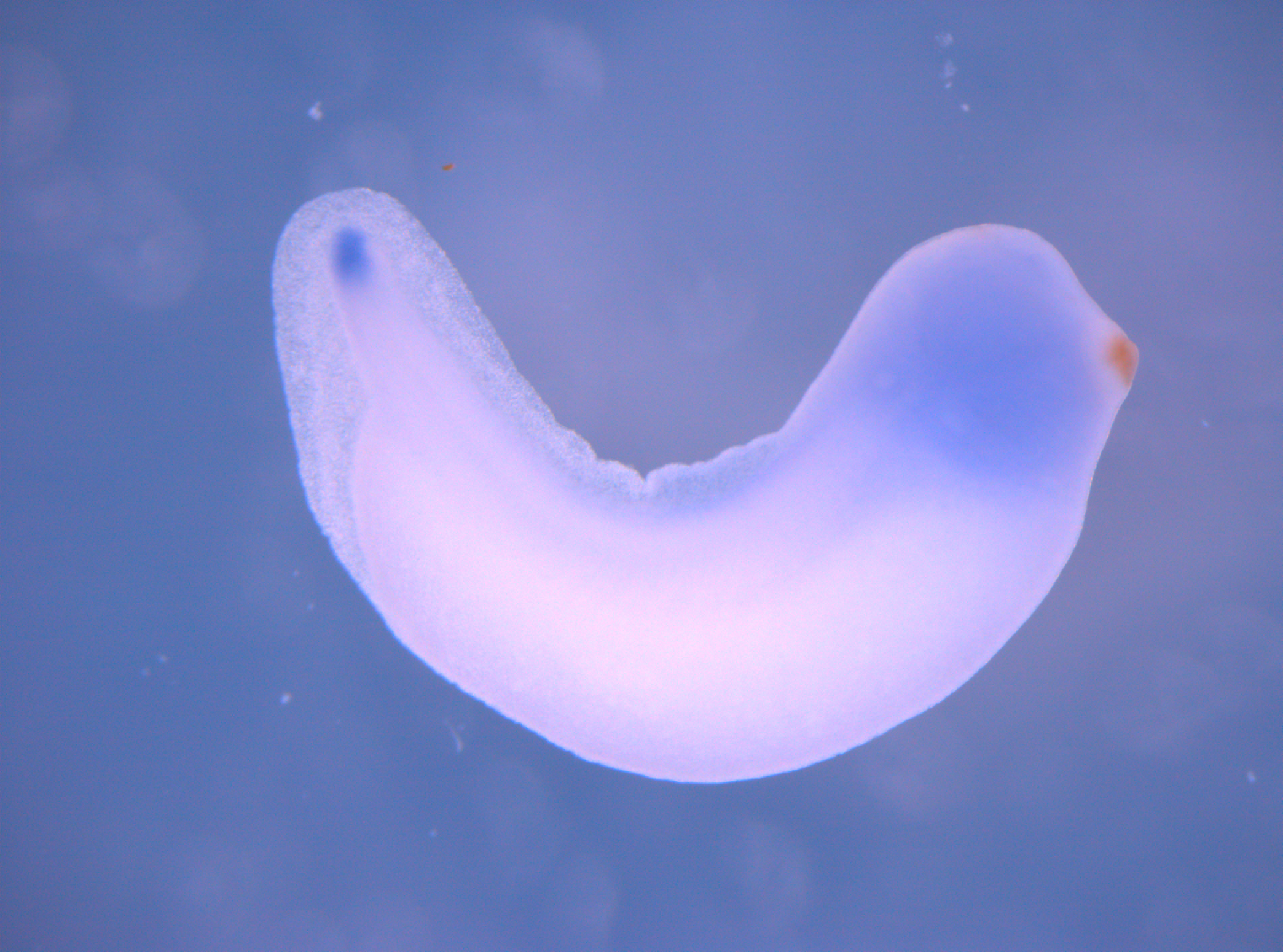

Supplement: Supplementary file 5 — Source Data for Figure 2 [file EMMM-15-e17078-s002.zip › Figure 2/2E_images embryos/Xbra/image0001.tif]

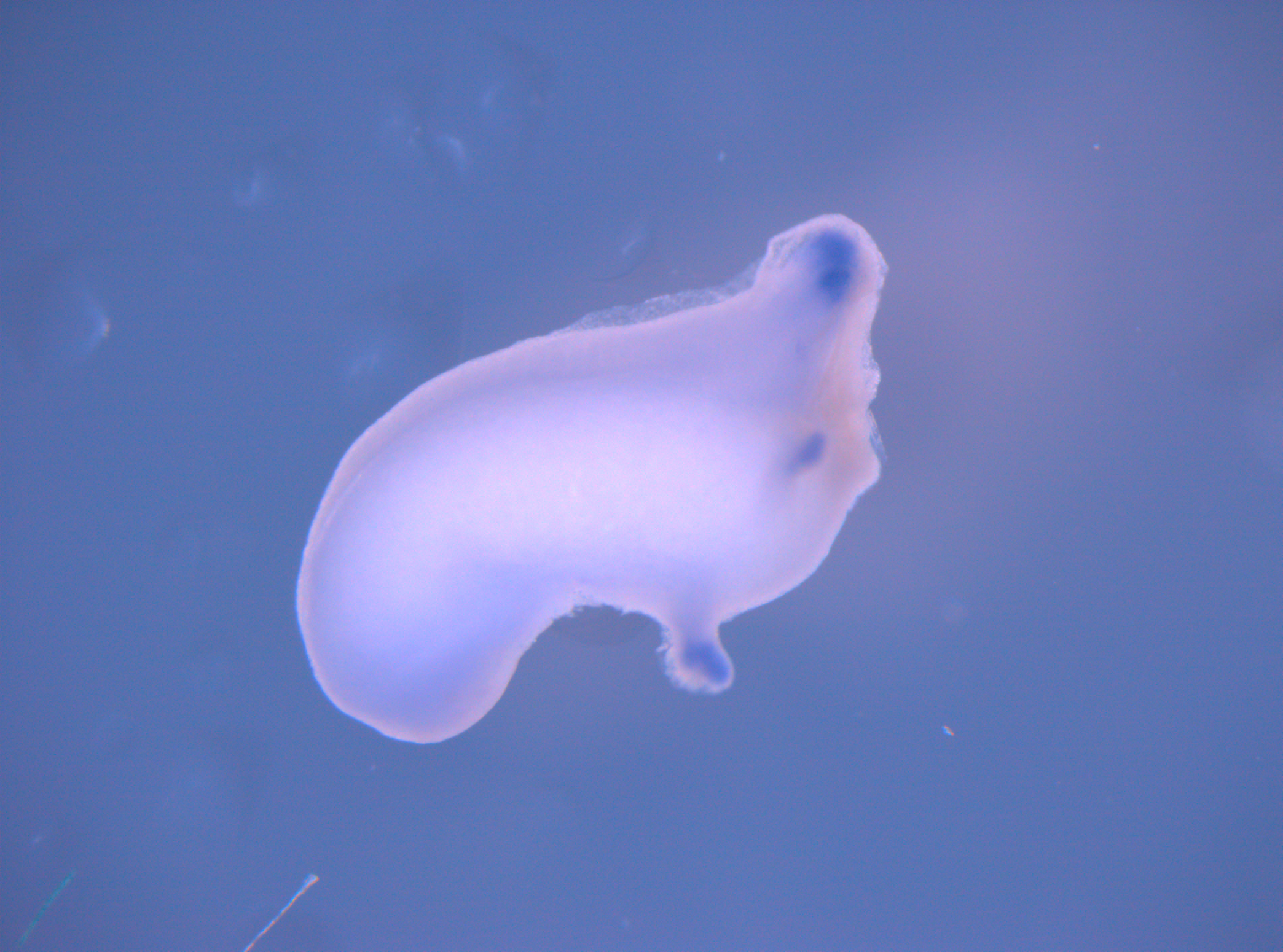

Supplement: Supplementary file 5 — Source Data for Figure 2 [file EMMM-15-e17078-s002.zip › Figure 2/2E_images embryos/Xbra/image0007.tif]

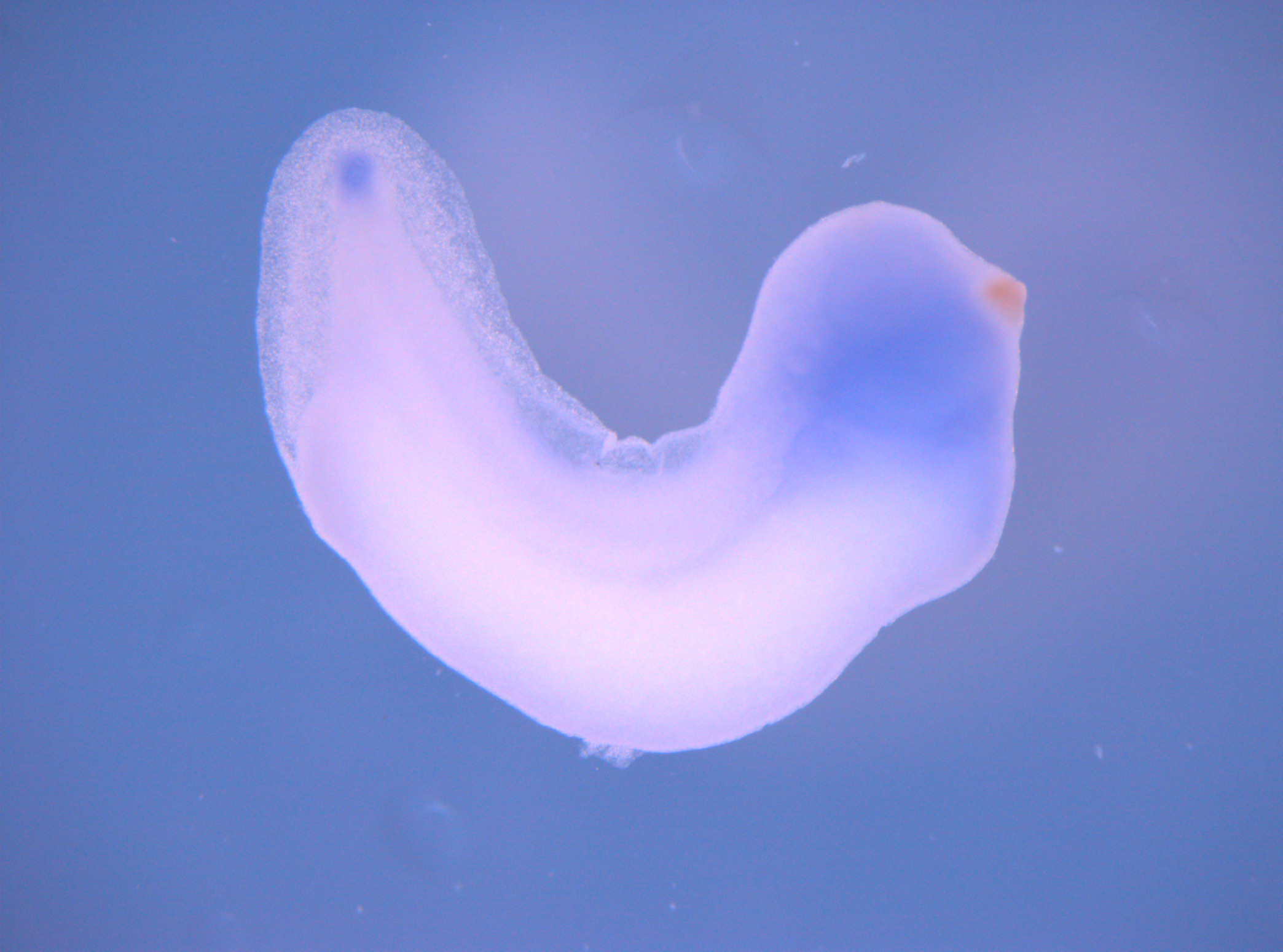

Supplement: Supplementary file 5 — Source Data for Figure 2 [file EMMM-15-e17078-s002.zip › Figure 2/2E_images embryos/Xbra/image0011.tif]

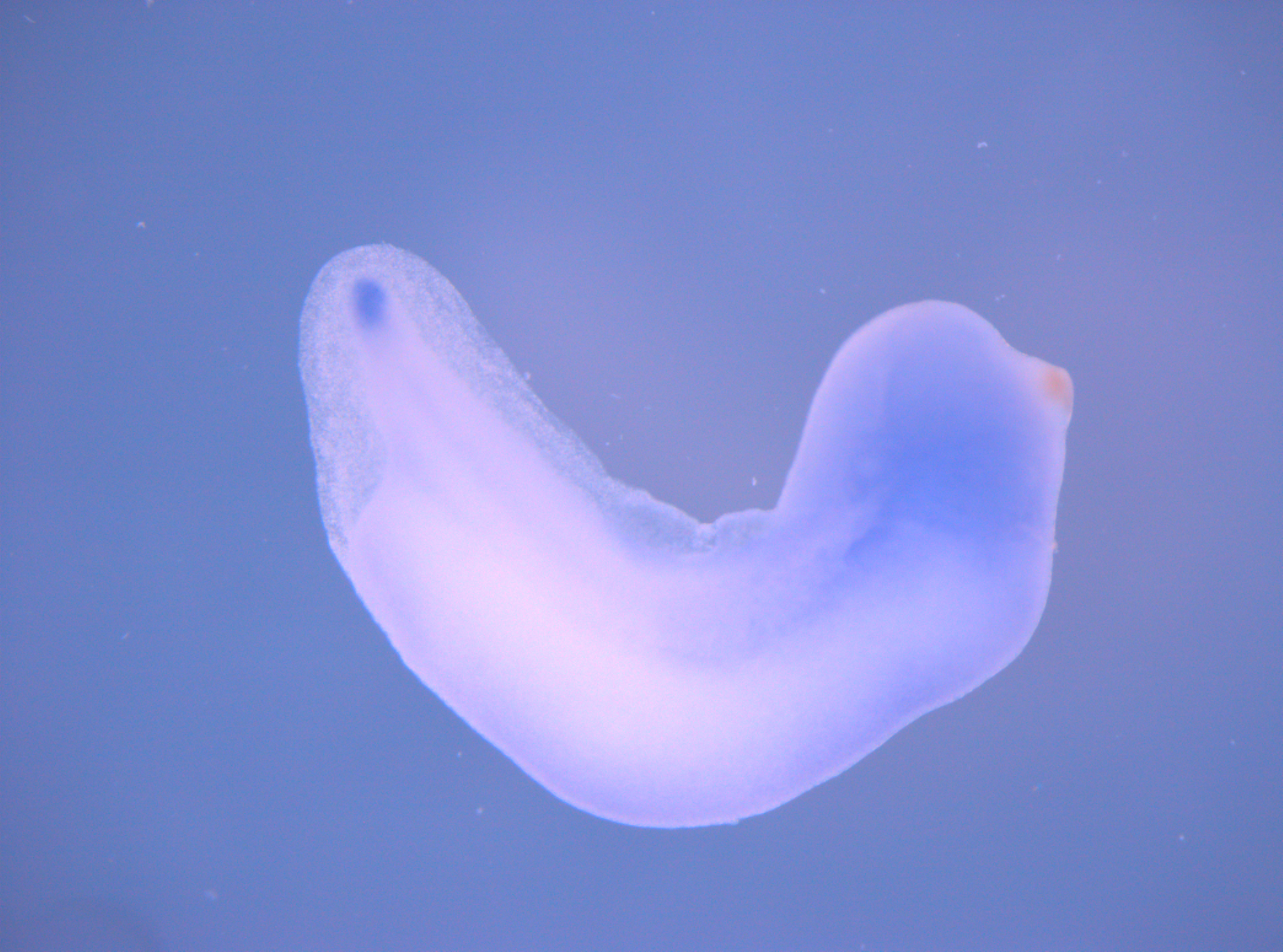

Supplement: Supplementary file 5 — Source Data for Figure 2 [file EMMM-15-e17078-s002.zip › Figure 2/2E_images embryos/Xbra/image0012.tif]

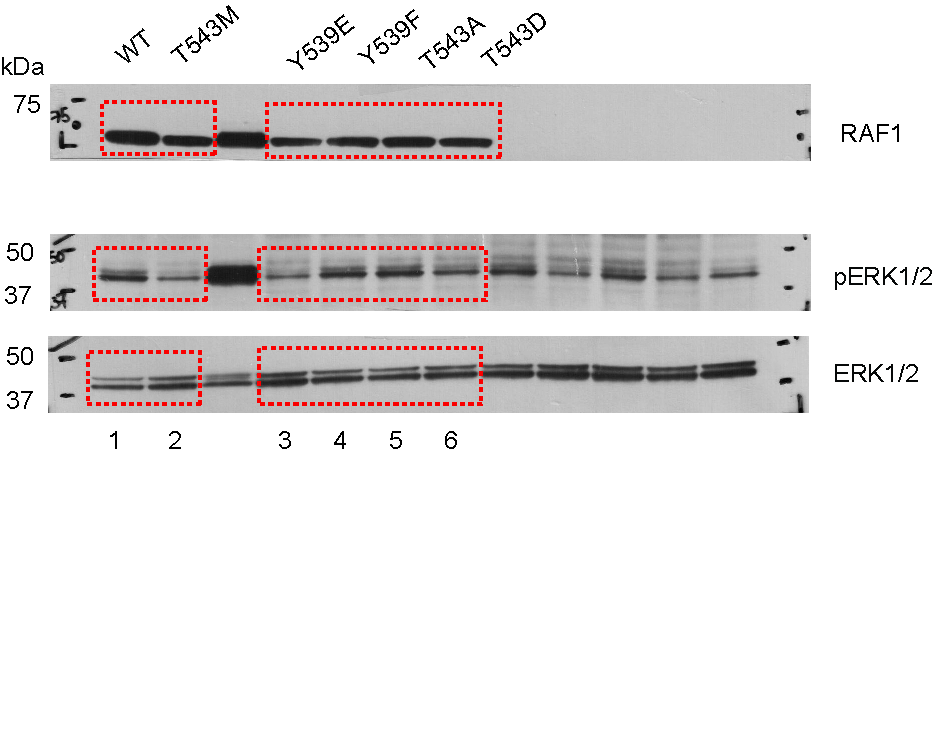

Supplement: Supplementary file 6 — Source Data for Figure 3 [file EMMM-15-e17078-s006.zip › Figure 3/3E_western blot/Fig 3E.tif]

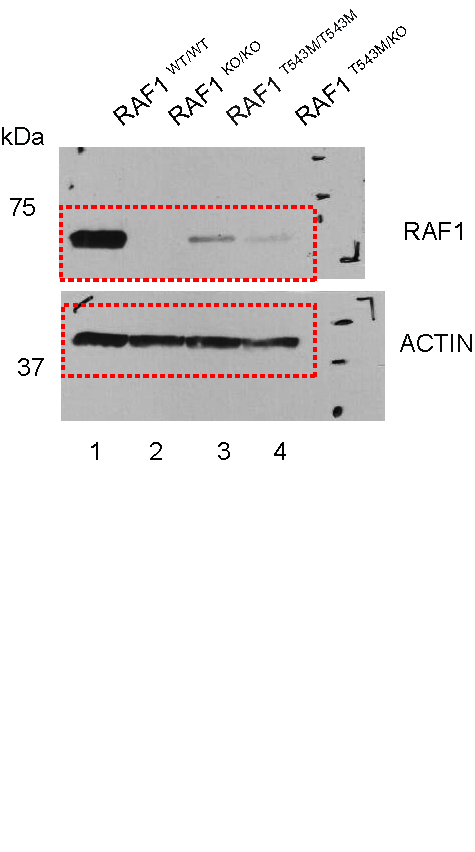

Supplement: Supplementary file 6 — Source Data for Figure 3 [file EMMM-15-e17078-s006.zip › Figure 3/3H_western blot/Fig 3H.tif]

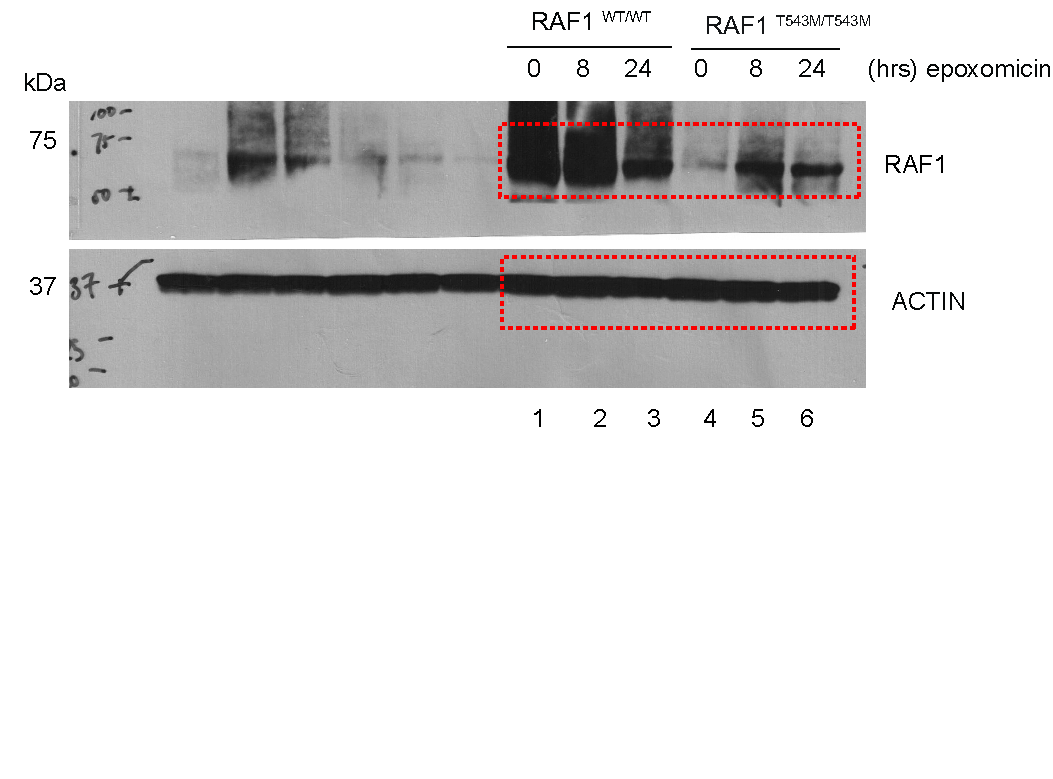

Supplement: Supplementary file 6 — Source Data for Figure 3 [file EMMM-15-e17078-s006.zip › Figure 3/3I_western blot/Fig 3I.tif]

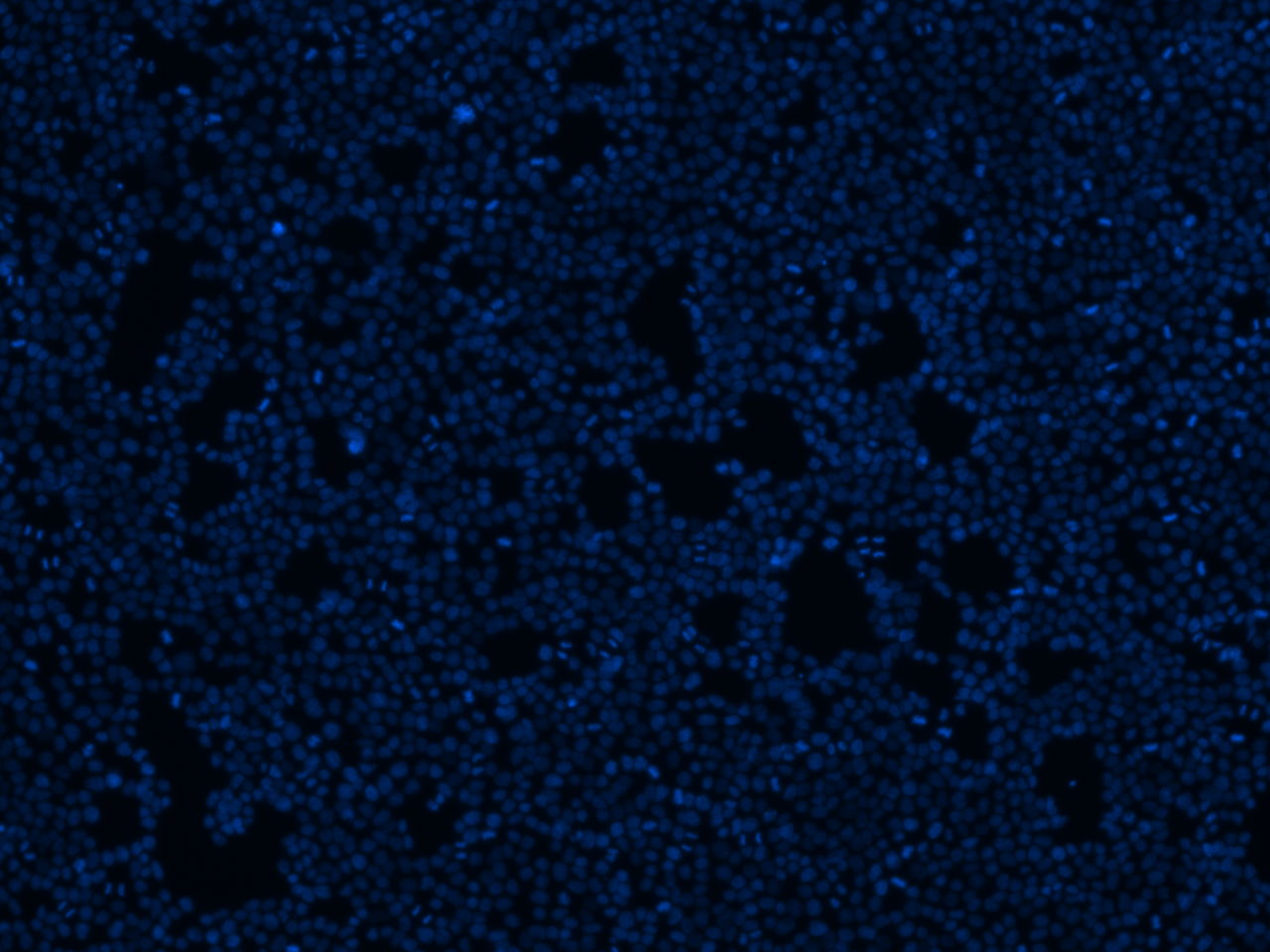

Supplement: Supplementary file 7 — Source Data for Figure 4 [file EMMM-15-e17078-s004.zip › Figure 4/4A_IF images/1_WT_0_d.tif]

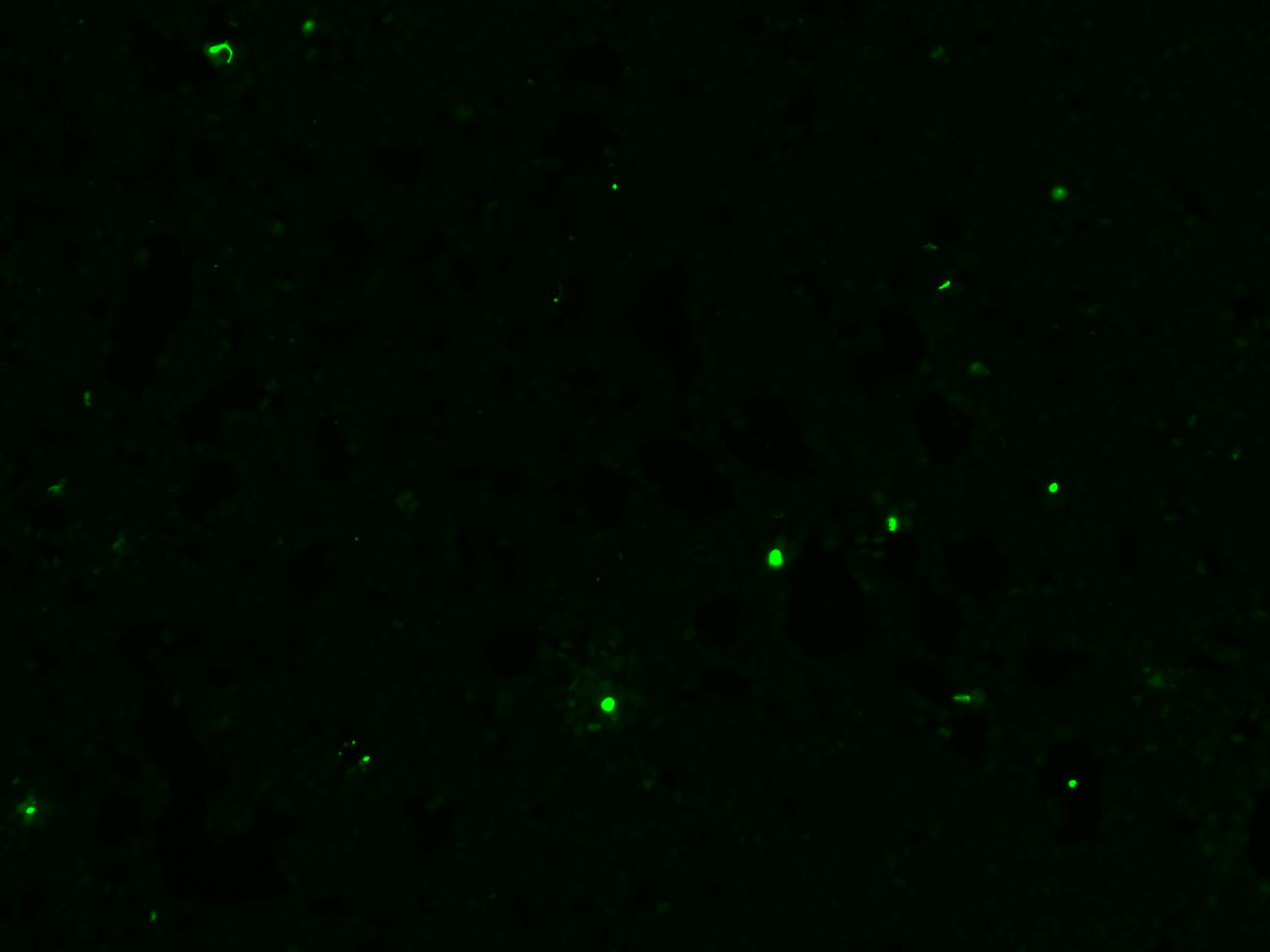

Supplement: Supplementary file 7 — Source Data for Figure 4 [file EMMM-15-e17078-s004.zip › Figure 4/4A_IF images/1_WT_0_s.tif]

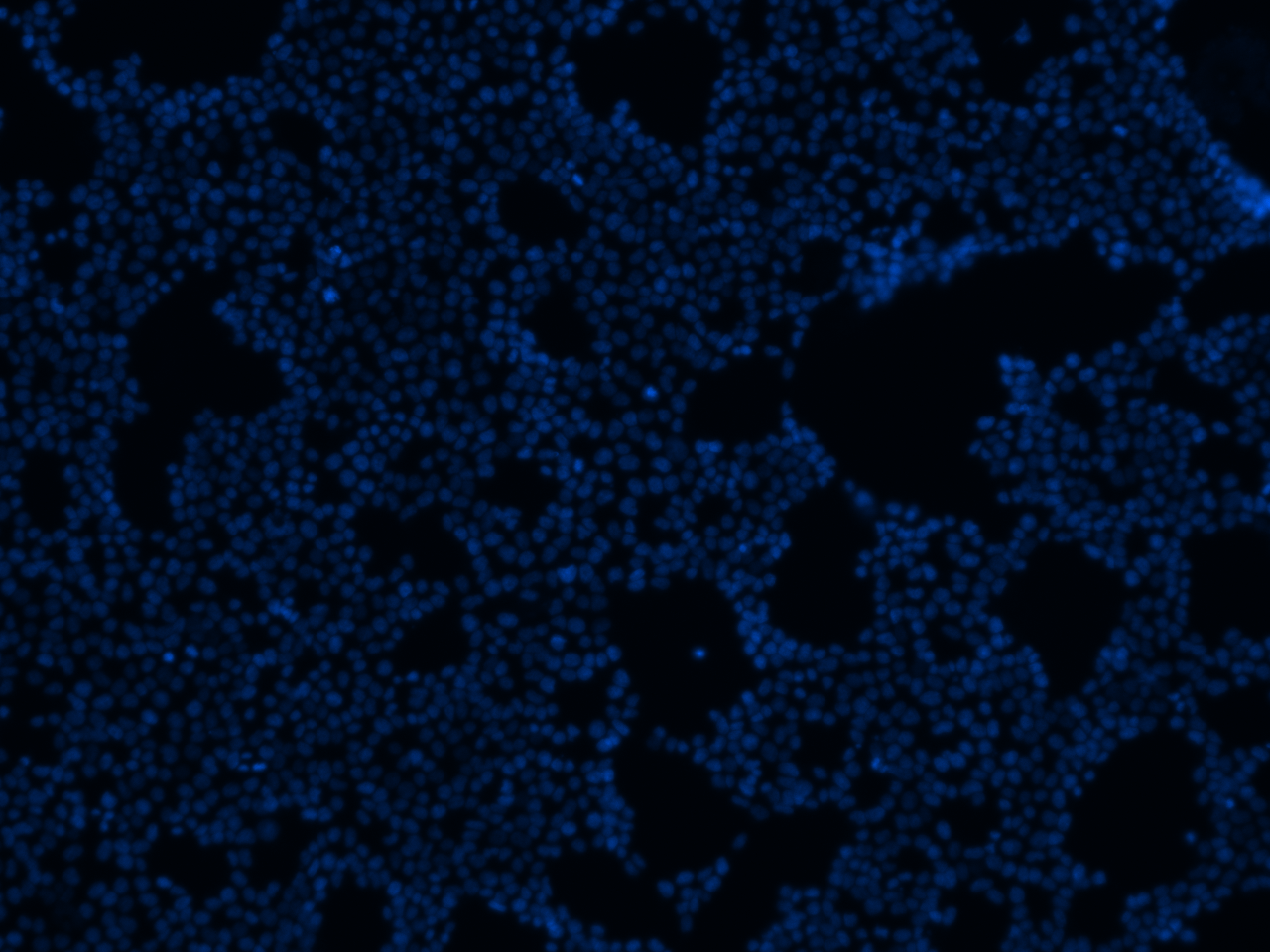

Supplement: Supplementary file 7 — Source Data for Figure 4 [file EMMM-15-e17078-s004.zip › Figure 4/4A_IF images/2_WT_0.2_d.tif]

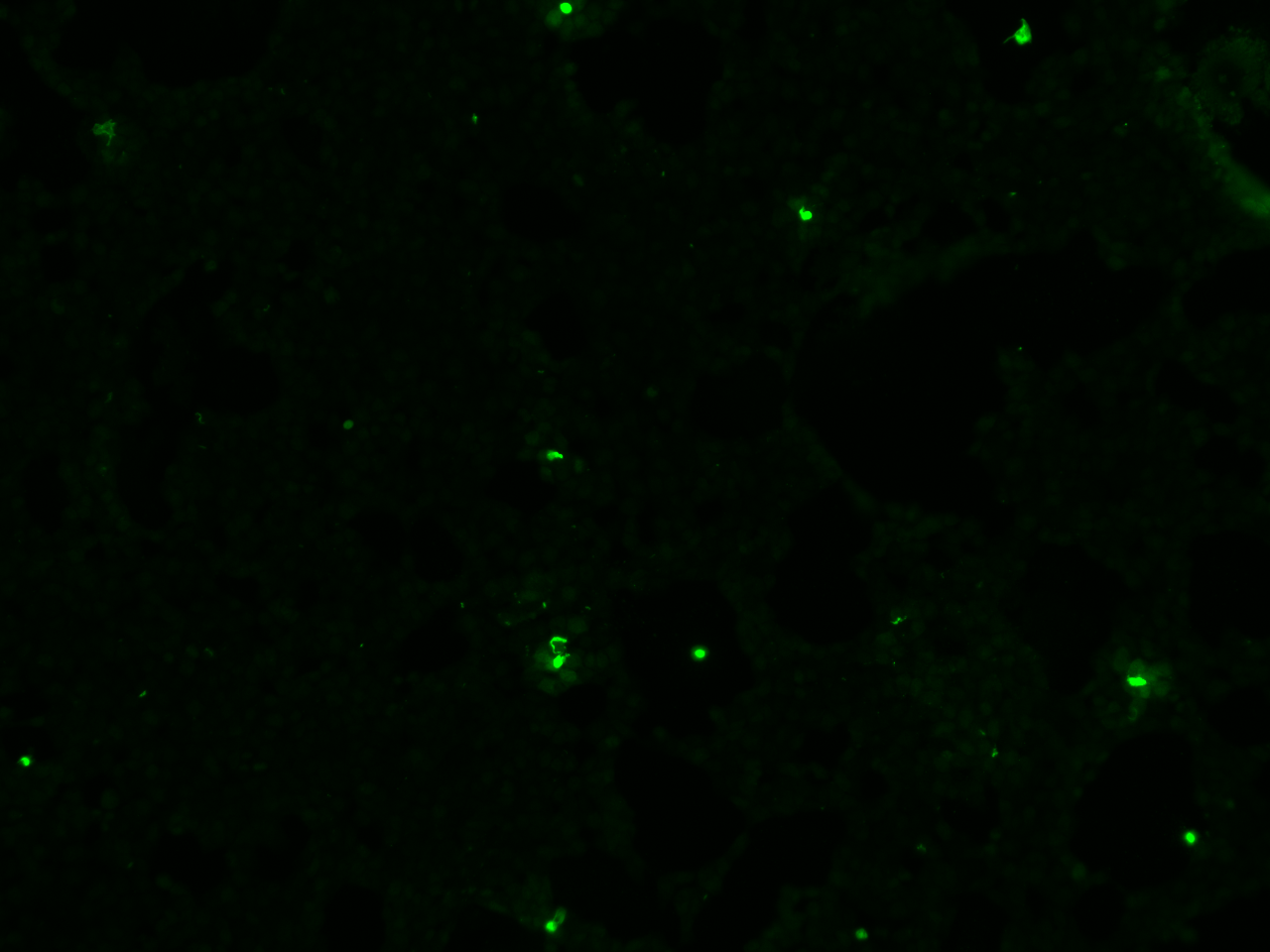

Supplement: Supplementary file 7 — Source Data for Figure 4 [file EMMM-15-e17078-s004.zip › Figure 4/4A_IF images/2_WT_0.2_s.tif]

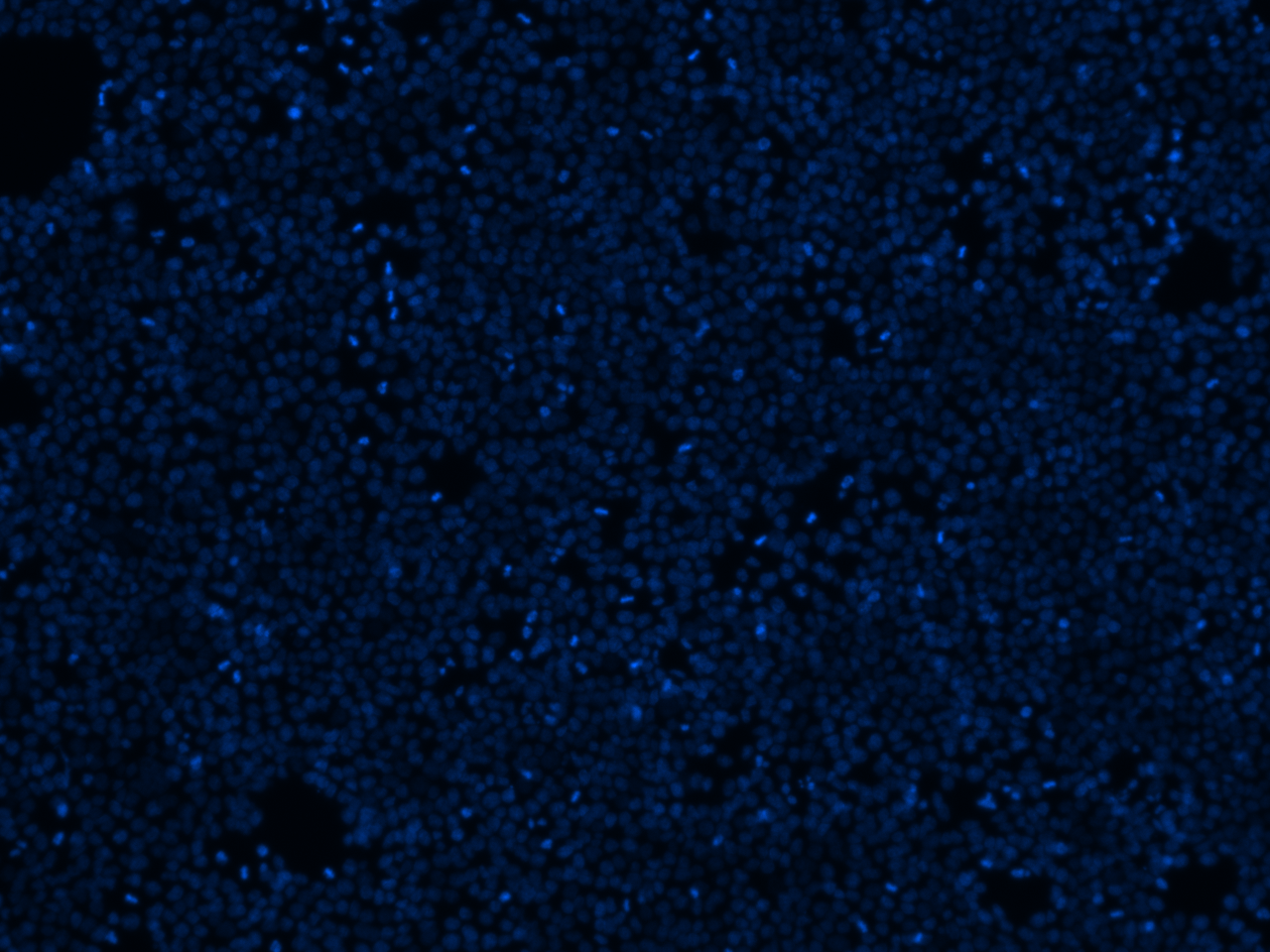

Supplement: Supplementary file 7 — Source Data for Figure 4 [file EMMM-15-e17078-s004.zip › Figure 4/4A_IF images/3_WT_0.8_d.tif]

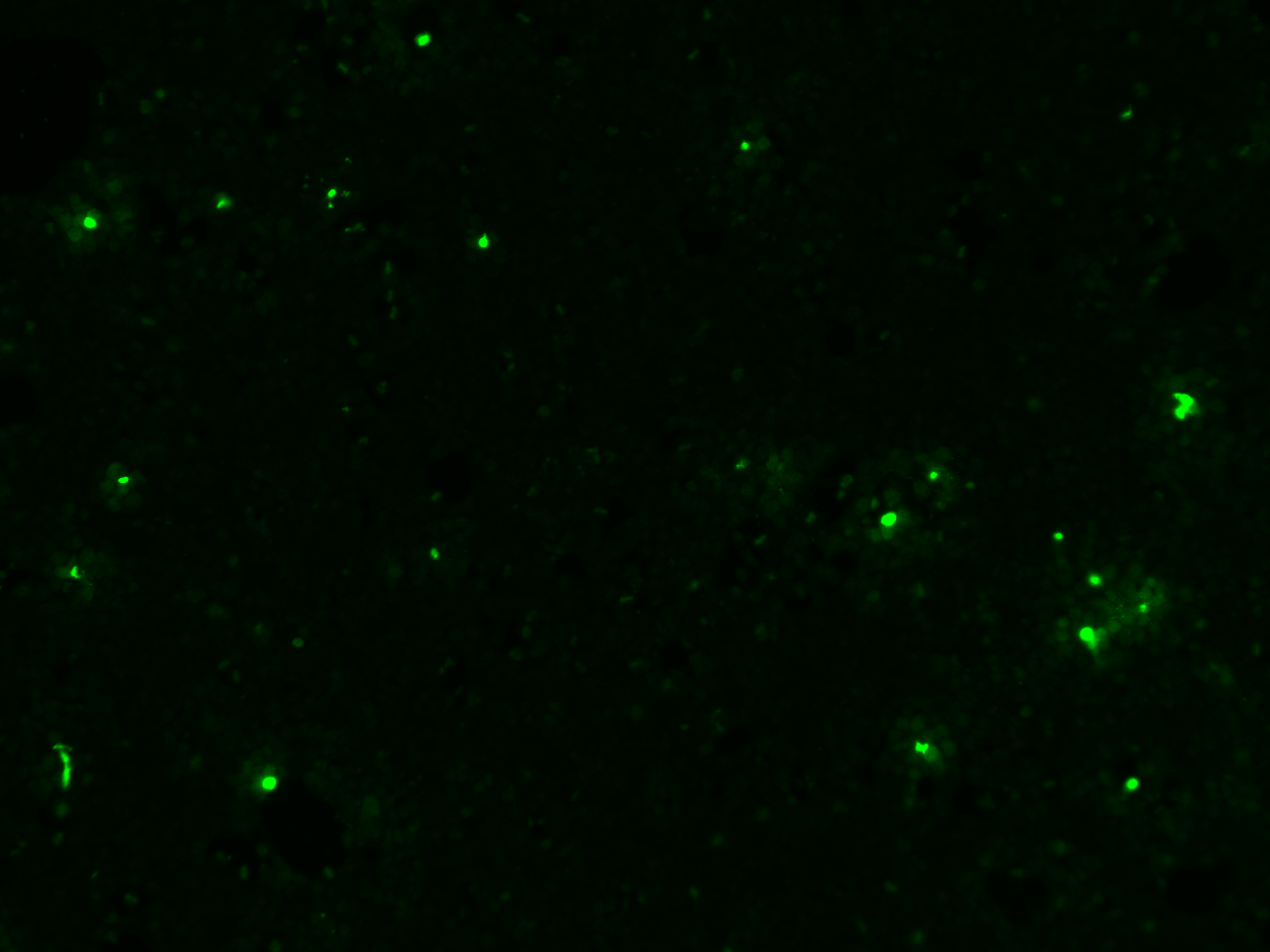

Supplement: Supplementary file 7 — Source Data for Figure 4 [file EMMM-15-e17078-s004.zip › Figure 4/4A_IF images/3_WT_0.8_s.tif]

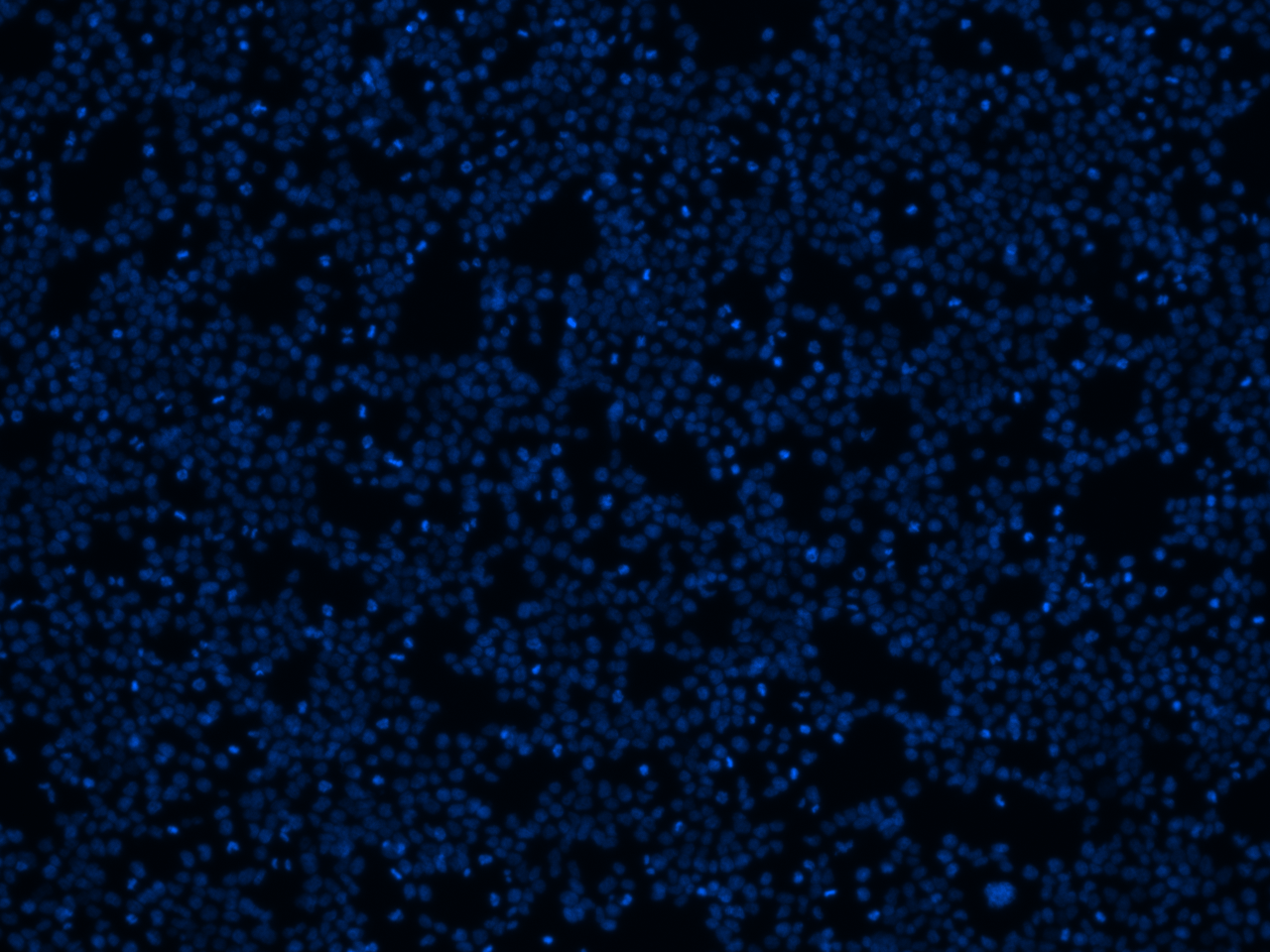

Supplement: Supplementary file 7 — Source Data for Figure 4 [file EMMM-15-e17078-s004.zip › Figure 4/4A_IF images/4_WT_1.6_d.tif]

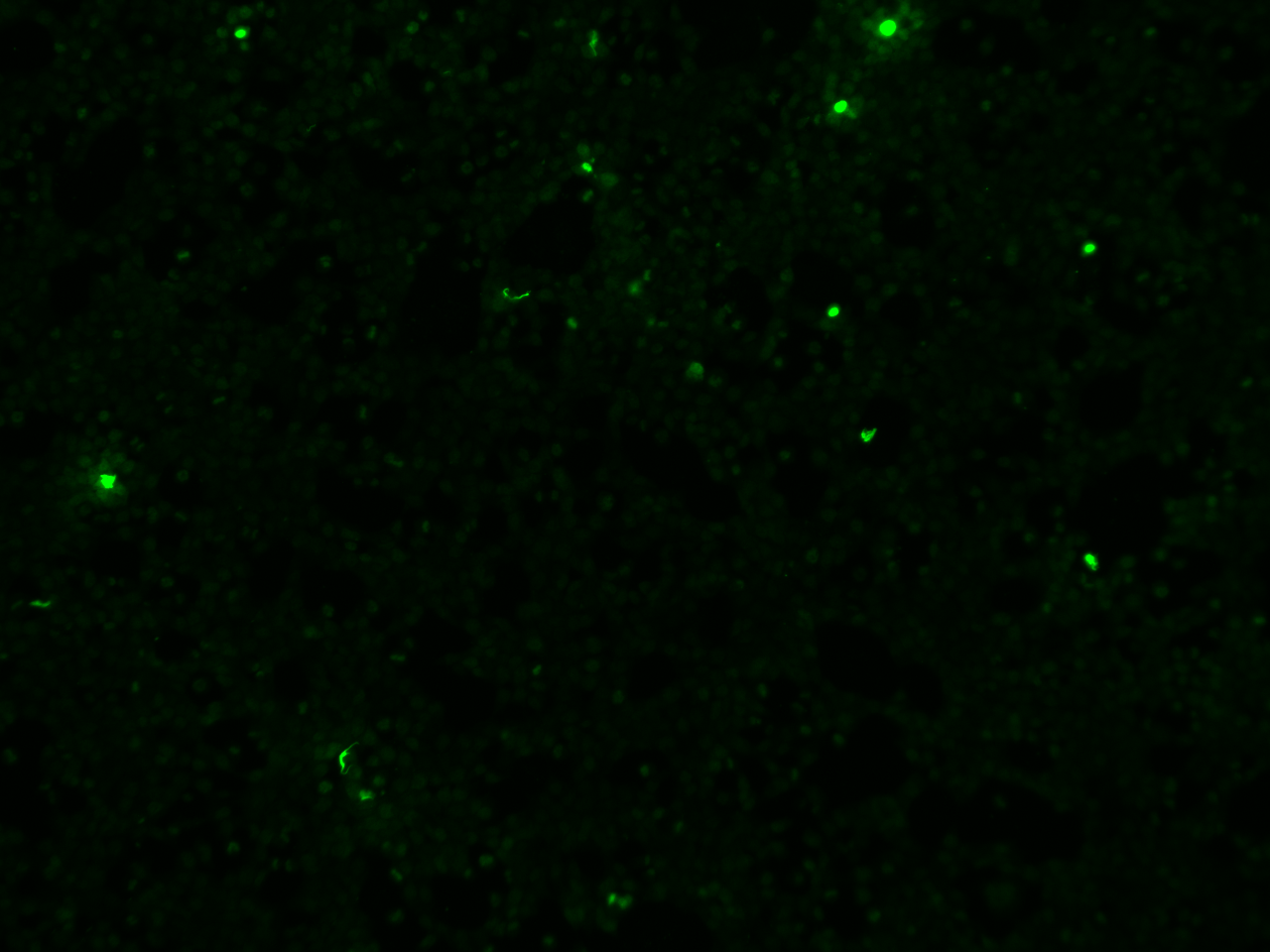

Supplement: Supplementary file 7 — Source Data for Figure 4 [file EMMM-15-e17078-s004.zip › Figure 4/4A_IF images/4_WT_1.6_s.tif]

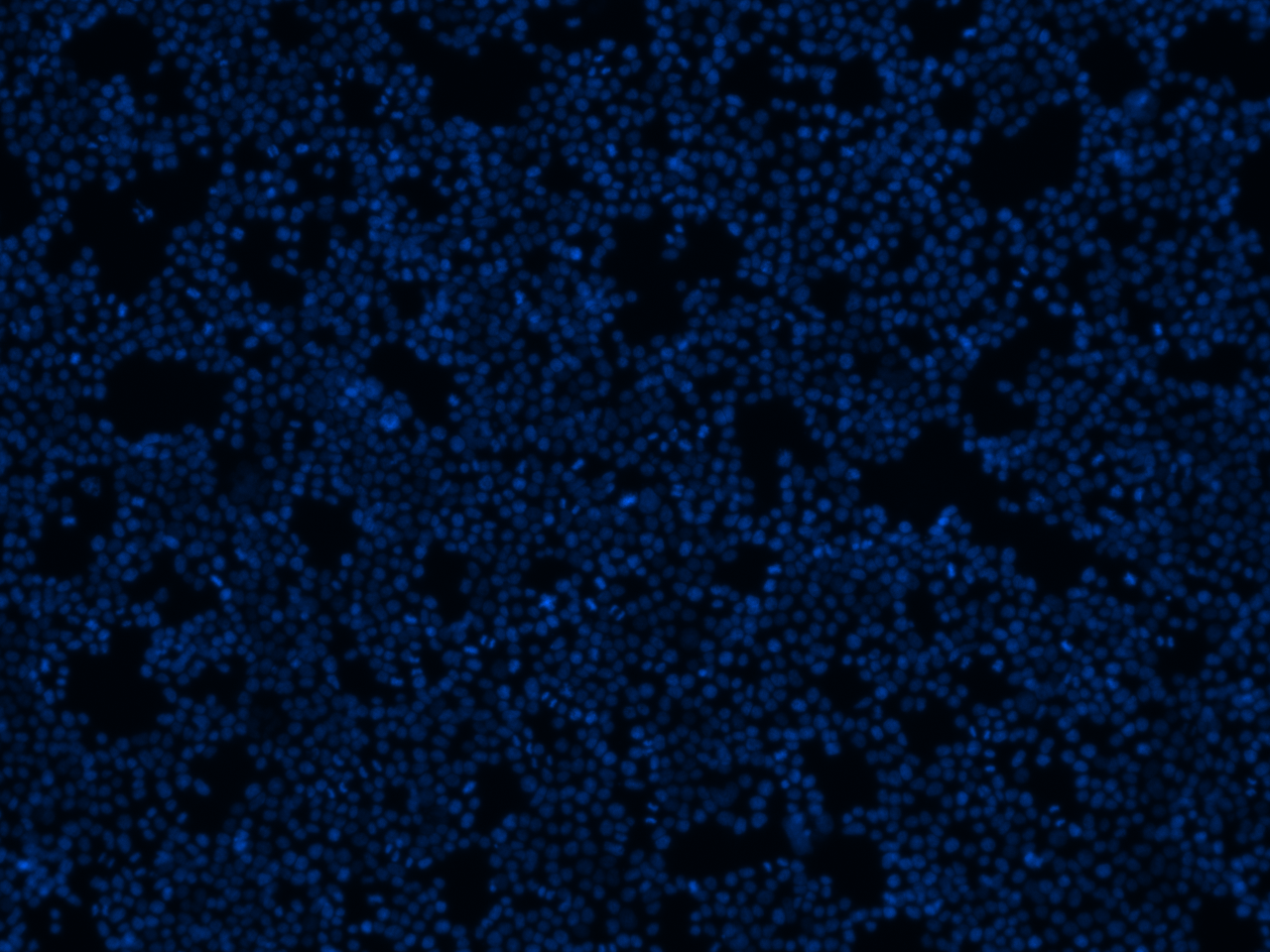

Supplement: Supplementary file 7 — Source Data for Figure 4 [file EMMM-15-e17078-s004.zip › Figure 4/4A_IF images/5_KI_0_d.tif]

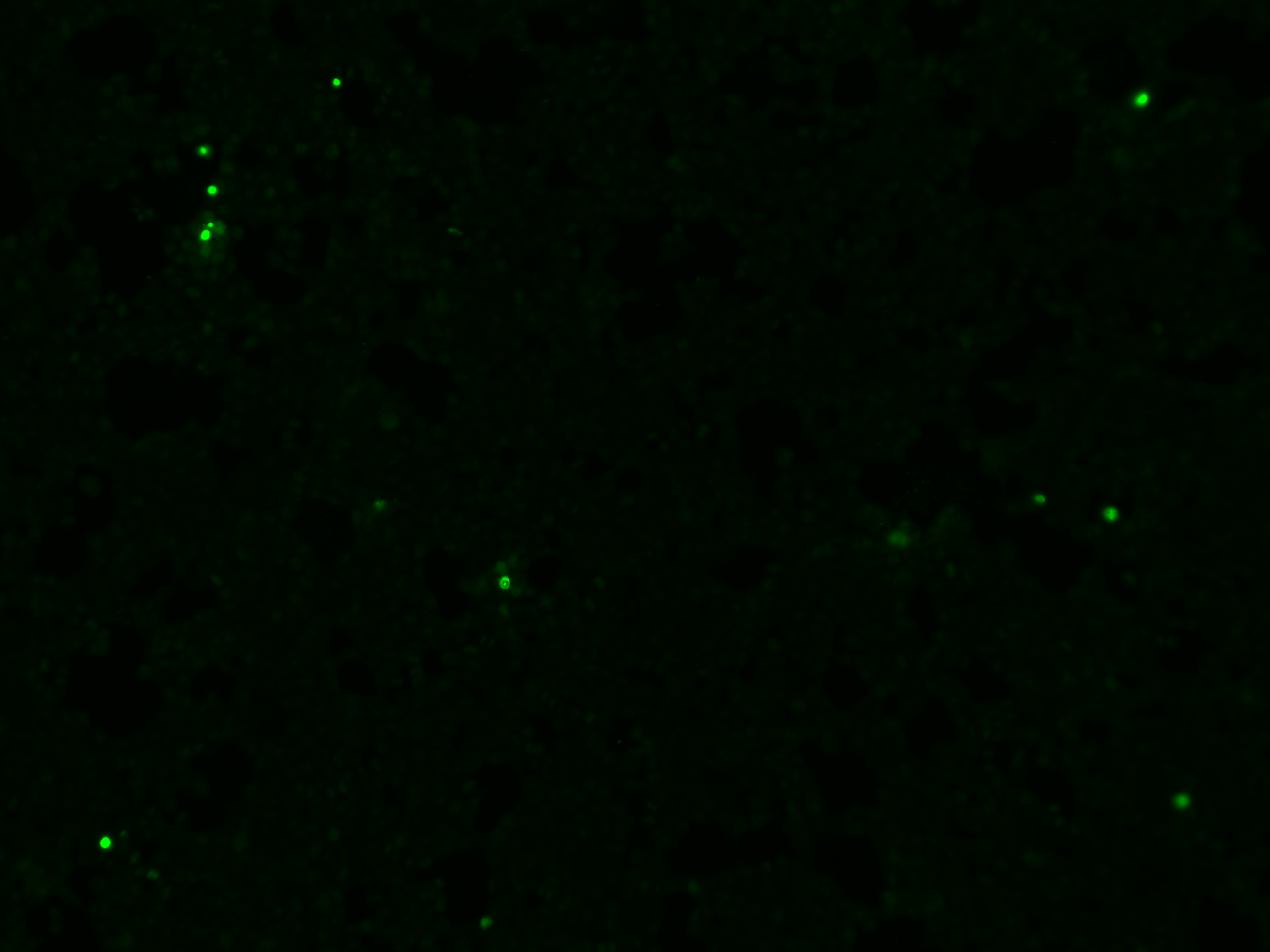

Supplement: Supplementary file 7 — Source Data for Figure 4 [file EMMM-15-e17078-s004.zip › Figure 4/4A_IF images/5_KI_0_s.tif]

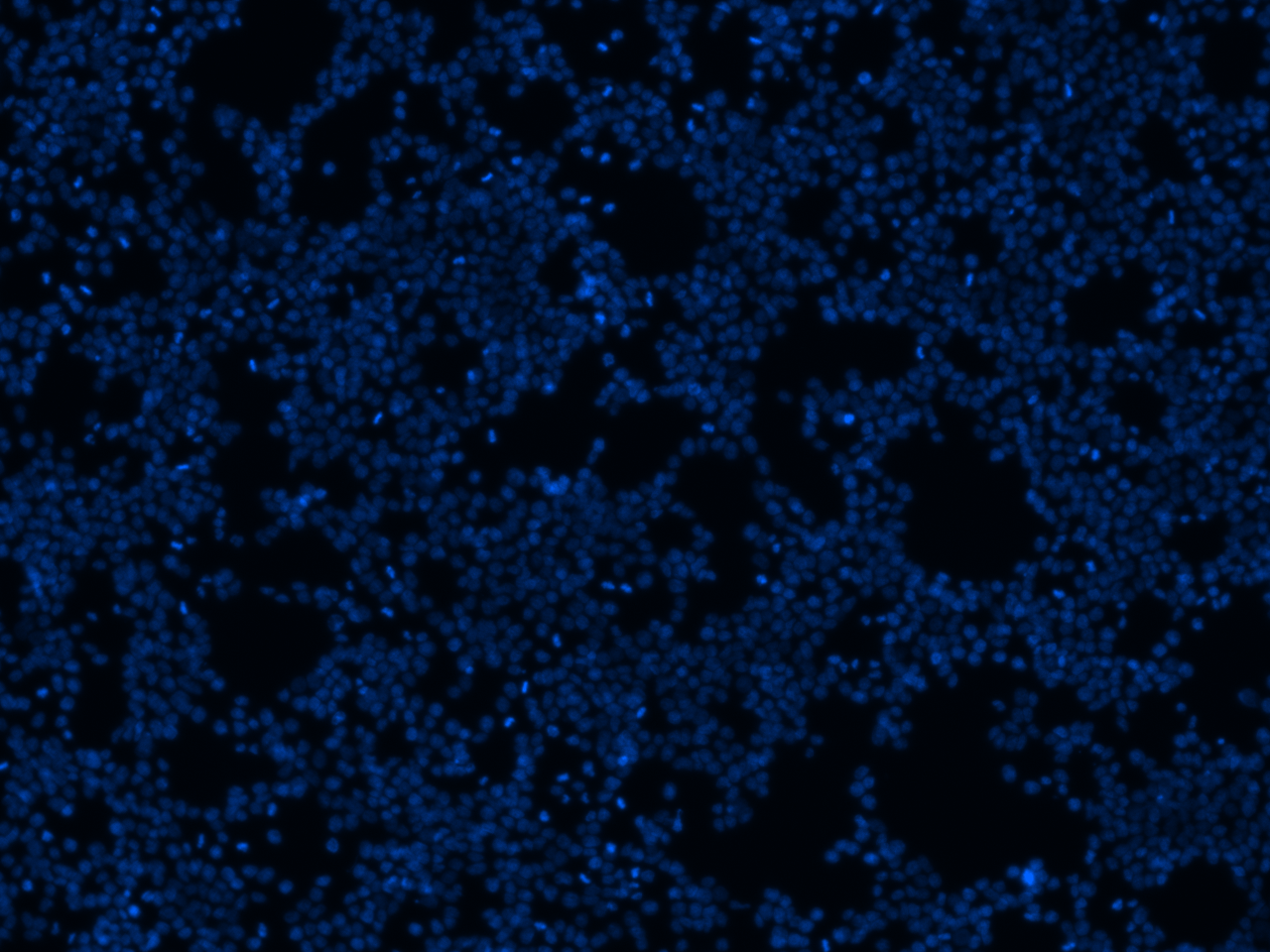

Supplement: Supplementary file 7 — Source Data for Figure 4 [file EMMM-15-e17078-s004.zip › Figure 4/4A_IF images/6_KI_0.2_d.tif]

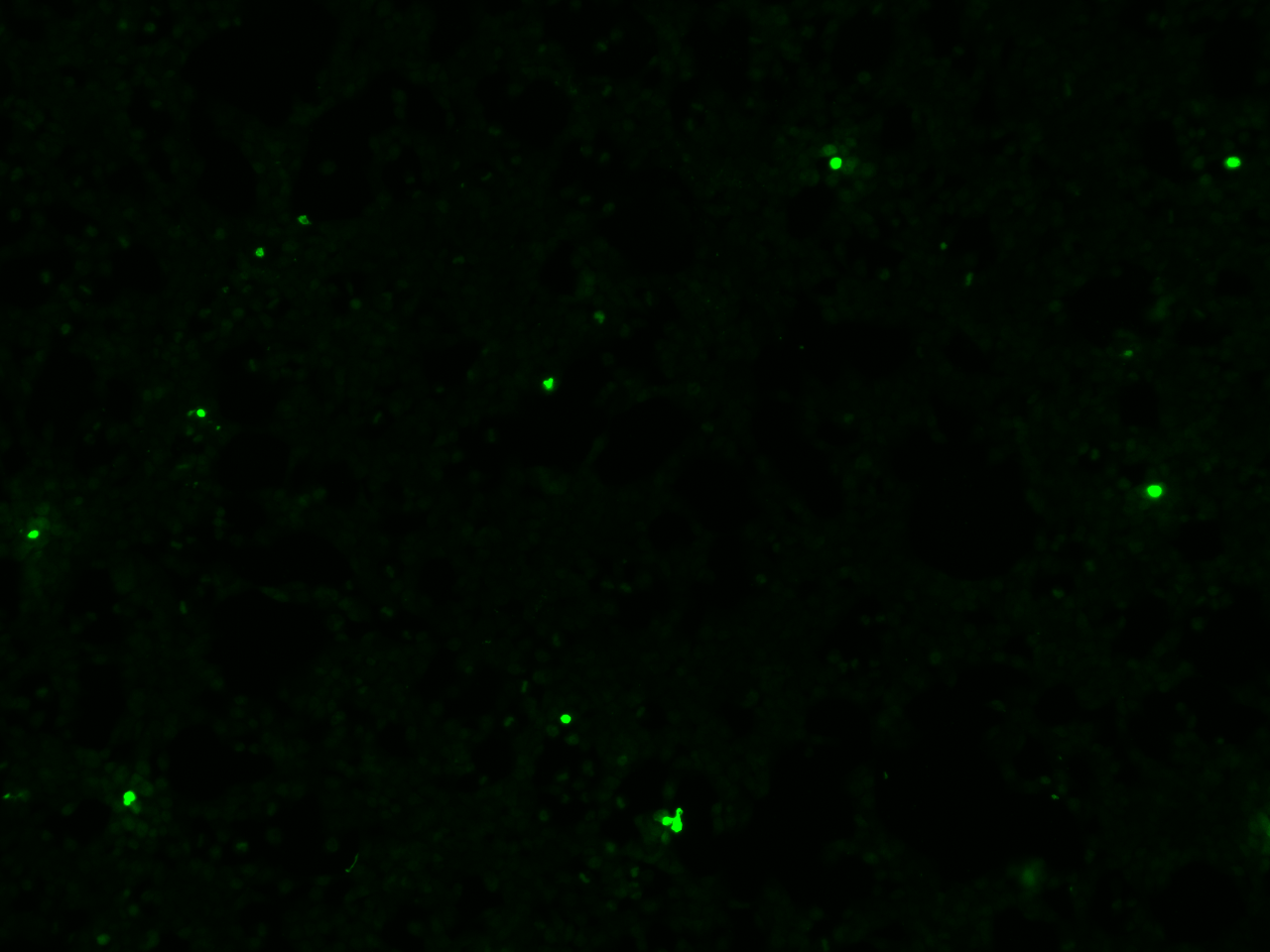

Supplement: Supplementary file 7 — Source Data for Figure 4 [file EMMM-15-e17078-s004.zip › Figure 4/4A_IF images/6_KI_0.2_s.tif]

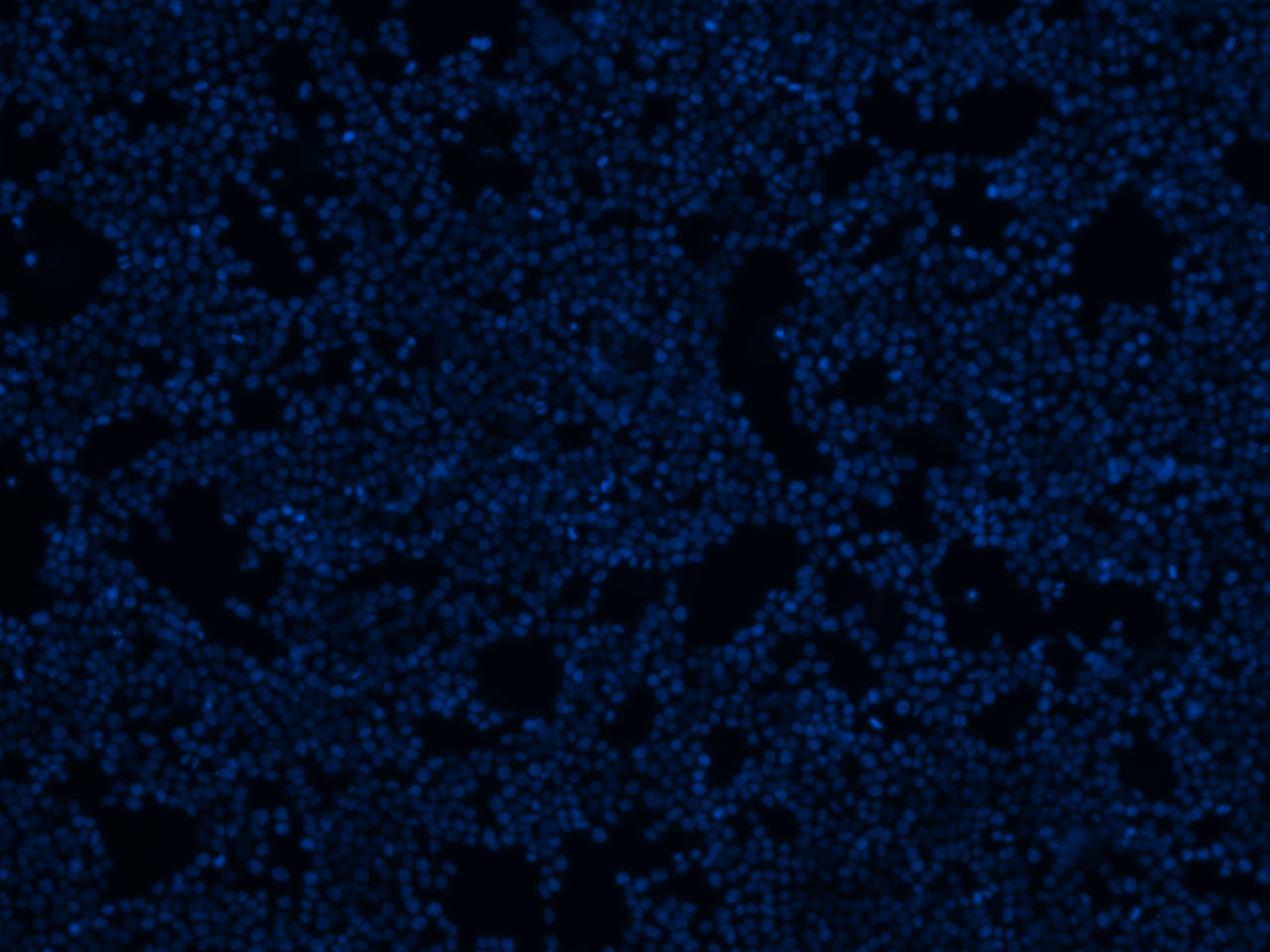

Supplement: Supplementary file 7 — Source Data for Figure 4 [file EMMM-15-e17078-s004.zip › Figure 4/4A_IF images/7_KI_0.8_d.tif]

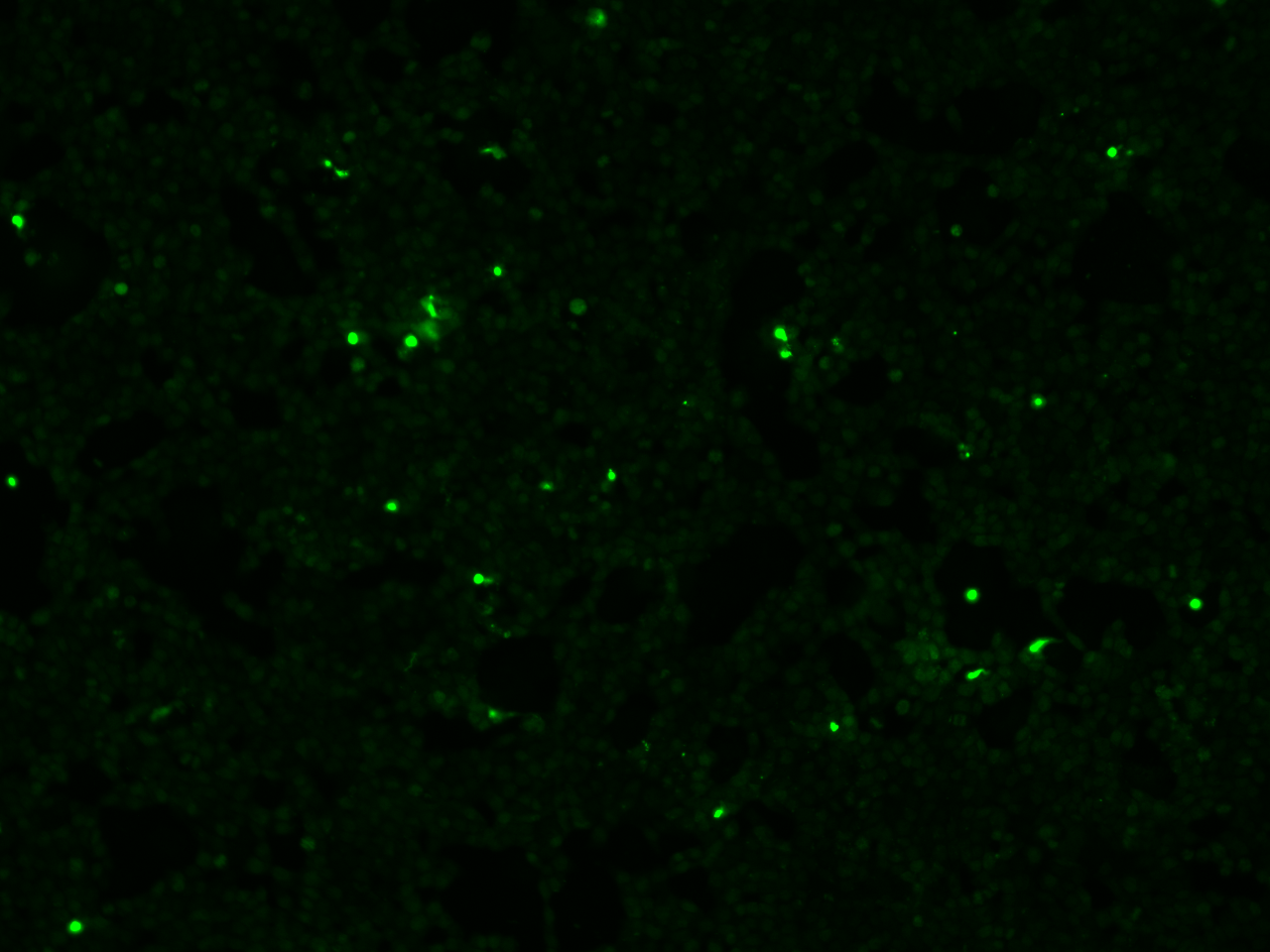

Supplement: Supplementary file 7 — Source Data for Figure 4 [file EMMM-15-e17078-s004.zip › Figure 4/4A_IF images/7_KI_0.8_s.tif]

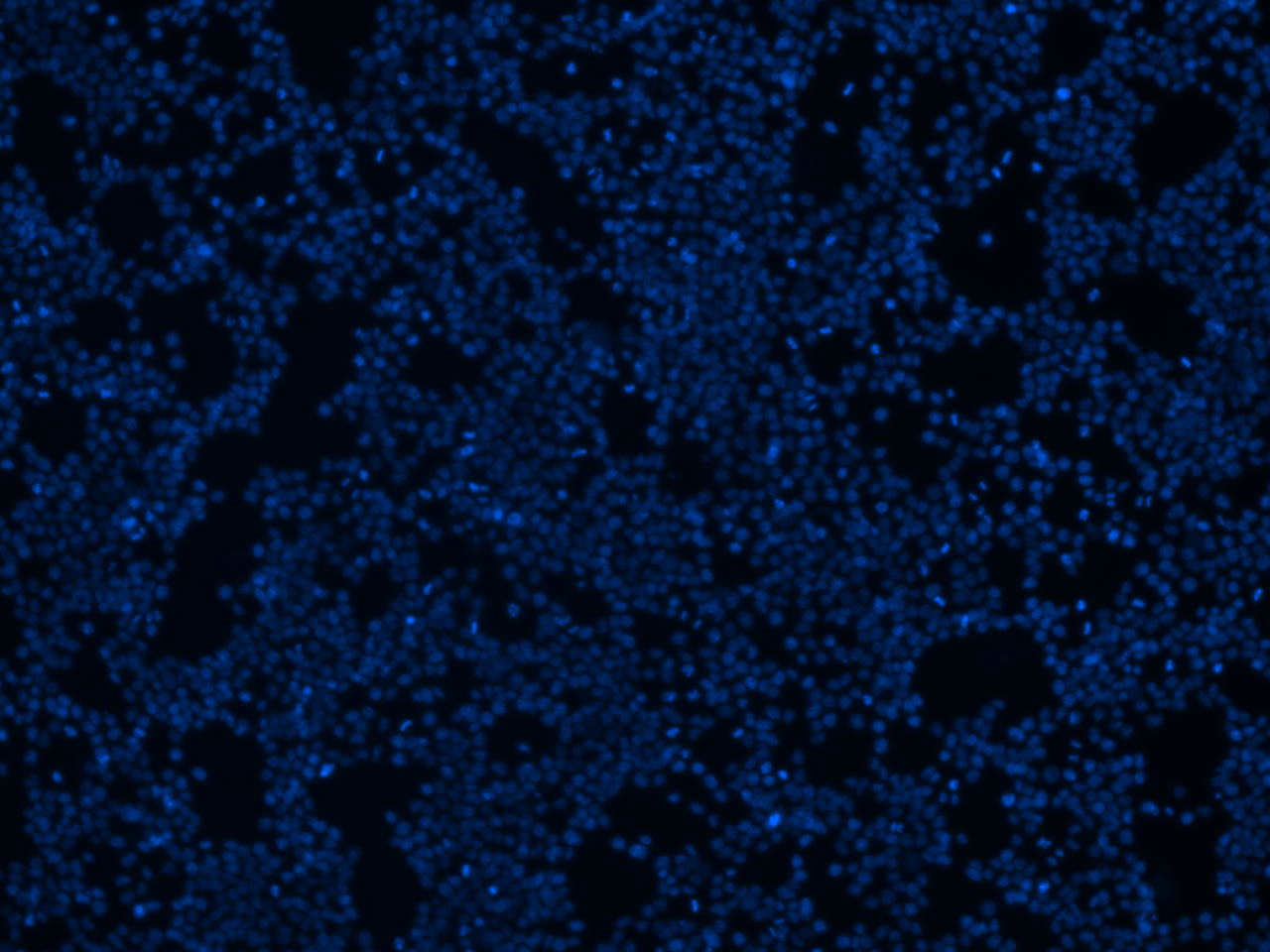

Supplement: Supplementary file 7 — Source Data for Figure 4 [file EMMM-15-e17078-s004.zip › Figure 4/4A_IF images/8_KI_1.6_4.tif]

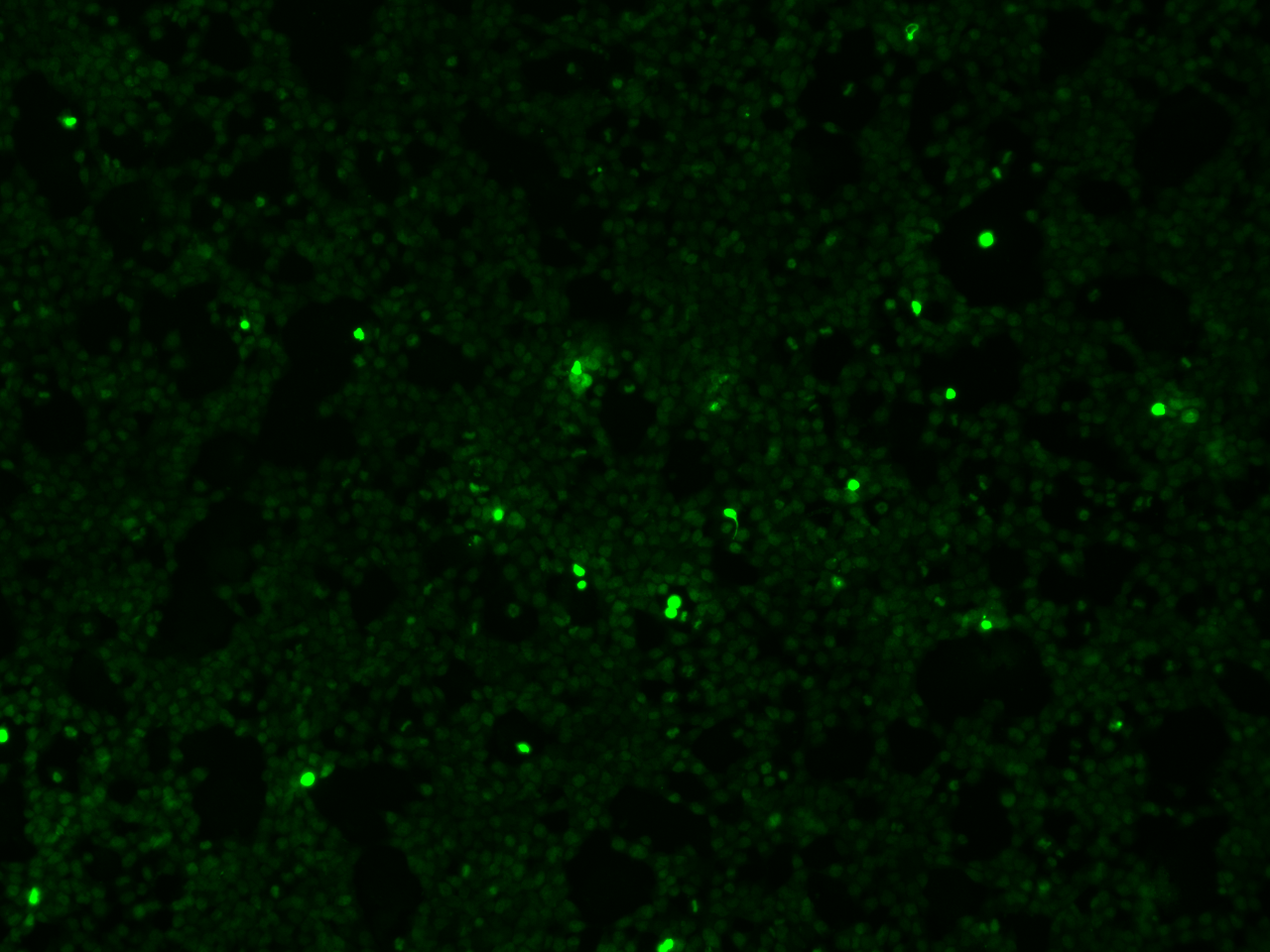

Supplement: Supplementary file 7 — Source Data for Figure 4 [file EMMM-15-e17078-s004.zip › Figure 4/4A_IF images/8_KI_1.6_s.tif]

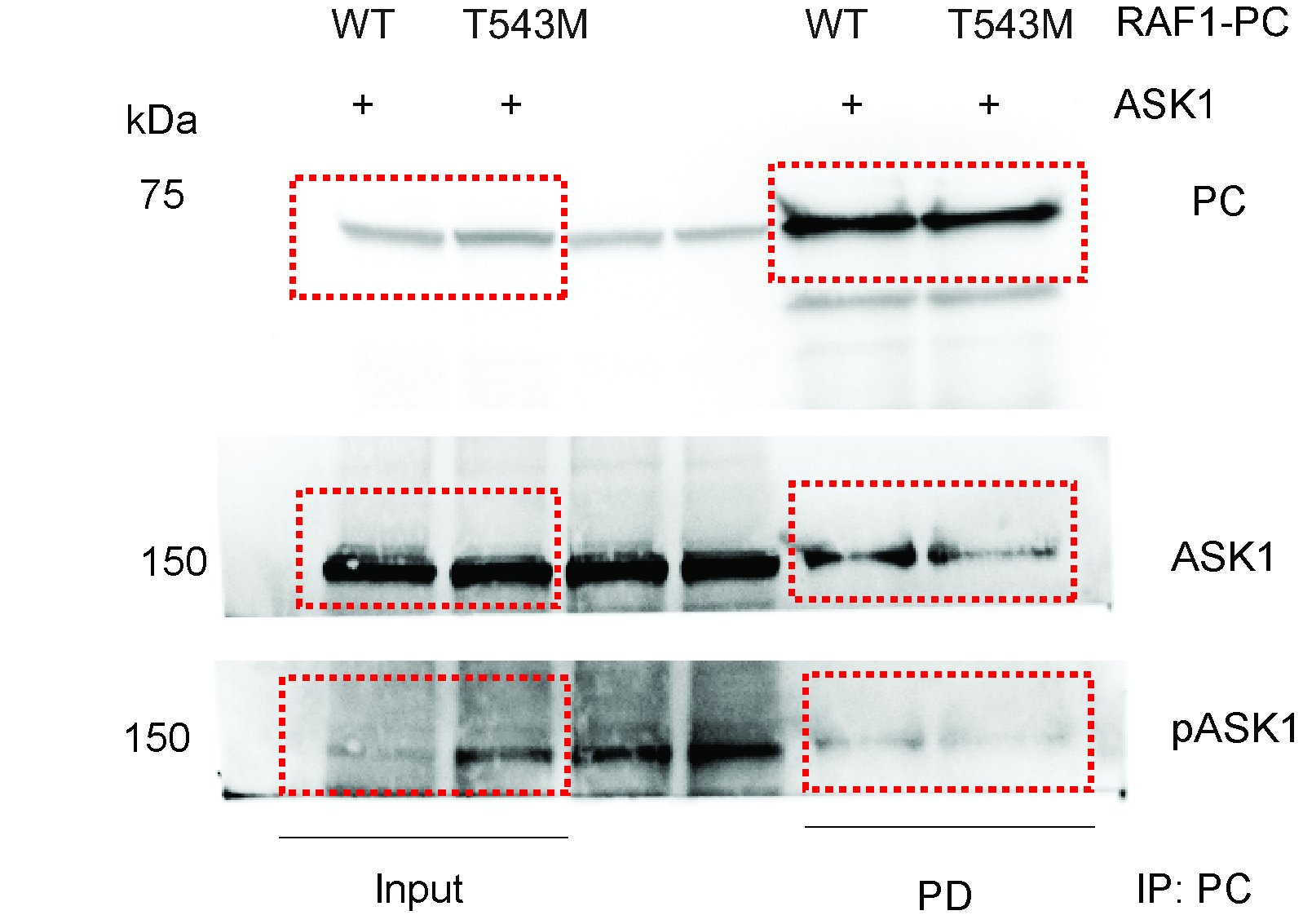

Supplement: Supplementary file 7 — Source Data for Figure 4 [file EMMM-15-e17078-s004.zip › Figure 4/4C_Western blot/Fig 4C.tif]

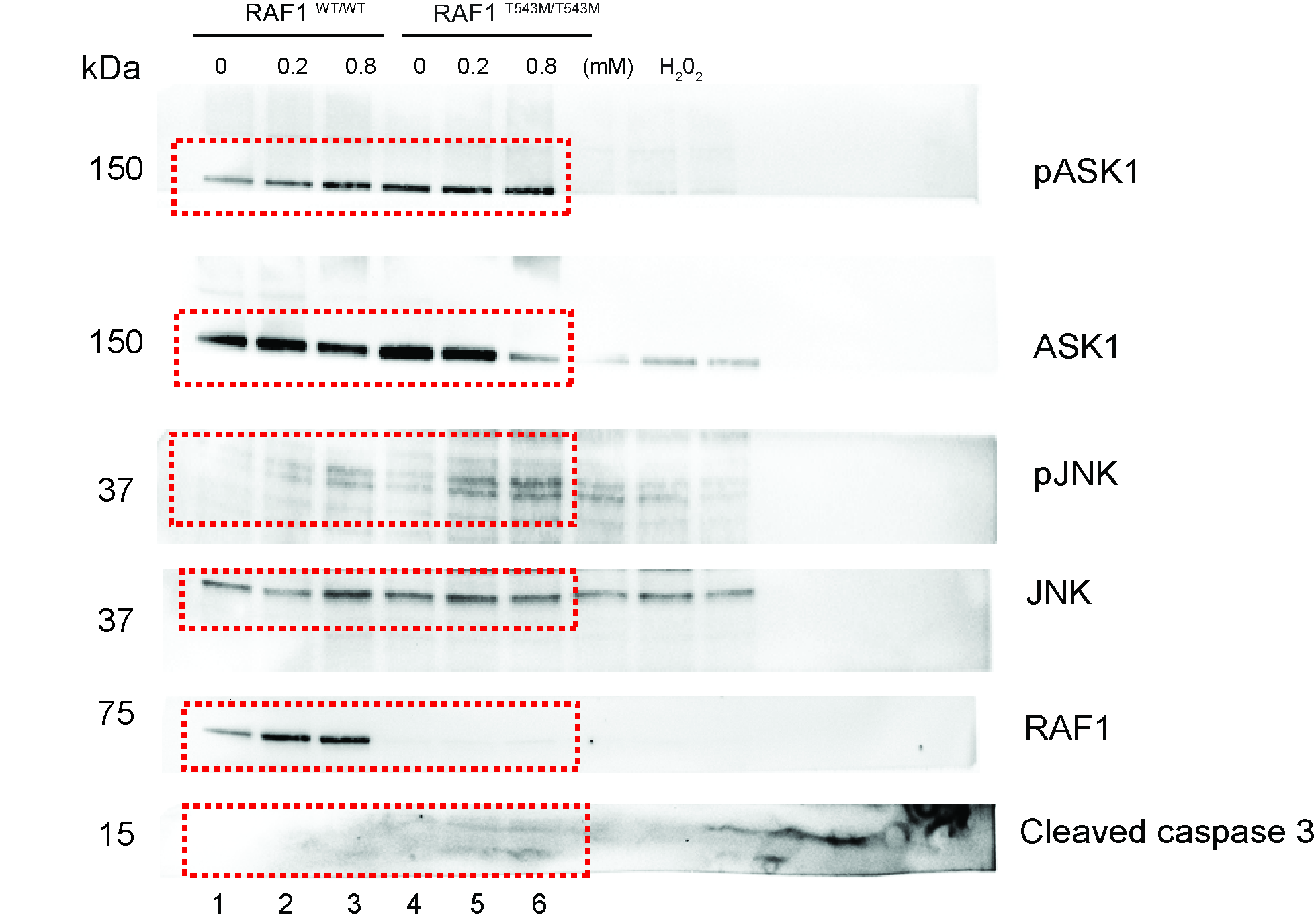

Supplement: Supplementary file 7 — Source Data for Figure 4 [file EMMM-15-e17078-s004.zip › Figure 4/4D_Western blot/Fig 4D.tif]
