# Supplementary material for: RAF1 deficiency causes a lethal syndrome that underscores RTK signaling during embryogenesis
Source: EMBO Mol Med. 2023 Apr 17;15(5):e17078. doi: 10.15252/emmm.202217078 (PMC10165362; doi:10.15252/emmm.202217078)
Supplement: Supplementary file 8 [file EMMM-15-e17078-s008.pdf]

EMBO Molecular Medicine

RAF1 deficiency causes a lethal syndrome that underscores RTK signaling during embryogenesis

Samantha WONG, Yu Xuan TAN, Abigail Loh, Kiat Yi TAN, Zainab AZIZ, Engin Özkan, Hulya Kayserili, Nathalie Escande-Beillard, Bruno REVERSADE, Stanley Nelson, and Hane Lee
DOI: 10.15252/emmm.202217078

Corresponding authors: Bruno REVERSADE (bruno@reversade.com) , Nathalie Escande-Beillard (nbeillard@ku.edu.tr), Hulya Kayserili (hkayserili@kuh.ku.edu.tr)

Review Timeline:

Submission Date:	20th Oct 22
Editorial Decision:	18th Nov 22
Revision Received:	3rd Jan 23
Editorial Decision:	17th Jan 23
Revision Received:	15th Mar 23
Accepted:	15th Mar 23

Editor: Lise Roth

Transaction Report:

Review COMMONS

This manuscript was transferred to EMBO Molecular Medicine following peer review at Review Commons.

(Note: With the exception of the correction of typographical or spelling errors that could be a source of ambiguity, letters and reports are not edited. Depending on transfer agreements, referee reports obtained elsewhere may or may not be included in this compilation. Referee reports are anonymous unless the Referee chooses to sign their reports.)

Review #1

1. Evidence, reproducibility and clarity:

Evidence, reproducibility and clarity (Required)

In the present manuscript, Wong et al describe for the first time the human phenotype resulting from the bi-allelic germline T543M mutation in the RAF1 proto-oncogene. Although somatic RAF1 mutations are outnumbered by BRAF mutations, they also occur in human cancer, but so far germline RAF1 mutations, usually dominant-acting ones abrogating RAF1 autoinhibition, have been mainly associated with RASopathies, in particular with Noonan syndrome. Here, Wong et al describe for the first time that the two individuals with a homozygous T543M mutation is associated with a neonatal lethal progeroid syndrome presenting with cutaneous, craniofacial, cardiac and limb
anomalies. Moreover, the affected residue, despite its conservation across RAF1 orthologues and in the ARAF and BRAF paralogues, has neither been described as a target of somatic and germline mutation in cancer and RASopathies, respectively.
The authors then use a combination of ectopic expression experiments in HEK293T cells and Xenopus embryos as well as CRISPR/Cas9 genome editing and structural bioinformatics to provide several lines of evidence that RAF1 T543M represents a hypomorph suppressing ERK activation.
In summary, this is a very interesting and well-written manuscript with a stimulating discussion of the novel and convincing findings and the mechanisms driving the described pathology. The suggestions below might further strengthen this otherwise already very advanced manuscript.

Major:

1. The data presented in this manuscript imply that RAF1 T543M represents a hypomorph suppressing ERK pathway activity. Nevertheless, it remains unclear whether this mutation reduces/abolishes the intrinsic activity of RAF1 or acts by a dominant-negative mechanism, e.g. by sequestrating critical components of RAF complexes, such as KSR proteins, or MEKs. To distinguish between both possibilities, it would be helpful, if the authors were able to document the intrinsic kinase activity of immunoprecipitated RAF1 T543M (and appropriate controls such as wildtype RAF1, the S257L gain-of-function allele and a truly kinase-dead variant, e.g. by mutating the aspartate of the DFG motif) towards recombinant and commercially available GST-MEK1.
2. RAF kinases engage in complex homo- and heterodimerization events. Does RAF1 T543M form homo- and heterodimers, e.g. with BRAF or ARAF, to a similar or different extent than wildtype RAF1?

Minor:

1. Abstract. To my knowledge, RAS but not RAF was the first human oncogene identified, albeit the discovery of RAF and its viral counterparts also took place very early in the oncogene discovery era.

2. Introduction, p. 2 "...while Braf-/- mice are embryonic lethal due to vascular defects.7" For a more balanced review, I would suggest citing additional and more recent papers describing the complex phenotypes of Braf deficient mice, e.g. PMIDs: 18332218 and PMID: 16432225
3. Figure 1D. Here, CRAF is used instead of RAF1. As the latter is the official gene/protein name RAF1 should be used to avoid confusion to readers outside of the RAF field.

2. Significance:

Significance (Required)

Very interesting novel findings to researchers being active in the signalling, cancer and developmental biology fiels as well as to human geneticists and pediatricians.

My expertise: MAPK signalling, functional characterization of oncogenic mutations

3. How much time do you estimate the authors will need to complete the suggested revisions:

Estimated time to Complete Revisions (Required)

(Decision Recommendation)

Between 1 and 3 months

4. Do you want to get recognition for this review on a Web of Science researcher profile?

Don't let your reviewing work go unnoticed! Researchers the world over use Web of Science Reviewer Recognition to effortlessly track their valuable peer review contributions for any journal. If you opt in, your Web of Science profile will automatically be updated to show a verified record of this review in full compliance with the journal's review policy. If you don't have a Web of Science profile, you will be prompted to create a free account.

Web of Science Reviewer Recognition

No

Review #2

1. Evidence, reproducibility and clarity:

Evidence, reproducibility and clarity (Required)

Summary:

The authors describe a homozygous missense variant in RAF1 identified in a child with a lethal malformation syndrome. The T543M variant not only blocks the kinase activity, but also destabilizes the RAF1 protein. In combination, this leads not only to a loss of MAPK signaling, but also to elevated apoptosis upon cell stress, which both together very likely explain the dramatic phenotype, which may correspond to the rarely described acro-cardio-facial syndrome.

Major comments:

The results of the functional analysis presented by the authors are highly convincing and clearly demonstrate a LOF effect of the identified variant. The experiments are described in sufficient detail and the statistical analysis is sound.
Depending on the target journal the clinical information might be a bit scarce. There are neither clinical pictures nor molecular data from the first affected sibling. For a more clinically oriented journal, a summary of clinical features in form of a table and a comparison to ACFS will be a prerequisite and facilitates appreciation of this very special phenotype.
The affected child seems much older than his chronological age, which seems due to the hypoplastic viscerocranium and the loss of subcutaneous fat tissue. The heart and limb defects have no link to chronological aging. Whether such phenotypes should be really called progeroid can be debated, but it is common practice (and always a good selling point). The authors do not discuss their findings in the light of chronological aging.
The hypothesis that the RAF1 LOF liberates ASK1 leading to increased apoptosis is very attractive. It would be very nice to show some experimental data proving this, but such data is not pivotal for the paper.

Minor comments:

The facial and overall progeroid phenotype of the affected child is quite different from most of the described ACFS patients. In contrast, the prominent neurocranium with low hairline and the hypoplastic viscerocranium remind this reviewer of Gorlin-Chaudhry-Moss/Fontaine-Petty syndrome. This differential diagnosis is also interesting since the disorder is caused by GOF variants in a mitochondrial ATP transporter increasing the sensitivity for apoptosis. This underlines the suggested link between RAF1 and the apoptosis inducer ASK1.
There is probably no cellular function that has not been linked to the MAPK pathway. One of these connections is to cellular aging. Strongly activating variants in pathway members lead to oncogene-induced cellular senescence. Here, we have a progeroid phenotype, but a variant with

LOF effect. This might be worthwhile to discuss.
The discussion could also use some sentences on the SHSF mechanism. FGFs produced by the AER are important for proliferation of the limb mesenchyme. What is the connection between MAPK and p63?

2. Significance:

Significance (Required)

This is a central signaling pathway and a prominent and historically outstanding oncogene. Up to now all variants in the MAPK pathway have been described as GOF and this is the first phenotype related to a LOF. Thus, this is a landmark paper widening the view on the pathway. The paper is interesting for clinical and molecular geneticists, tumor biologists, and cell biologists.
This reviewer is a biochemist and clinical geneticist with research focus on mechanisms of skeletal and connective tissue disorders.

3. How much time do you estimate the authors will need to complete the suggested revisions:

Estimated time to Complete Revisions (Required)

(Decision Recommendation)

Less than 1 month

4. Do you want to get recognition for this review on a Web of Science researcher profile?

Don't let your reviewing work go unnoticed! Researchers the world over use Web of Science Reviewer Recognition to effortlessly track their valuable peer review contributions for any journal. If you opt in, your Web of Science profile will automatically be updated to show a verified record of this review in full compliance with the journal's review policy. If you don't have a Web of Science profile, you will be prompted to create a free account.

Web of Science Reviewer Recognition

Yes

Review #3

1. Evidence, reproducibility and clarity:

Evidence, reproducibility and clarity (Required)

The manuscript by Wong et al., provides the first report of a homozygous loss-of-function mutation of the RAF1 gene in humans. The mutation (T543M) was found in two siblings of a consanguineous family in association with perinatal death and multiple developmental abnormalities. These abnormalities show strong similarities with a rare congenital malformation syndrome with unknown aetiology named Acro-cardio-facial syndrome (ACFS, MIM600460). Conversely, reported abnormalities are different from those observed in RASopathies, congenital diseases caused by gain-of-function mutations in either the RAF gene or genes implicated in the same signaling pathway (MAPK) and that induce its ectopic/over-activation. By performing functional experiments in cellular systems (cell line where the mutated RAF was either overexpressed or knocked-in) and in Xenopus Laevis embryos, the authors demonstrate that the reason for the phenotypic differences compared to RASopathies is that the RAF1T543M variant impairs the signaling activity of RAF and blunts MAPK pathway activation. In particular, the RAF1T543M variant: 1) is not actively phosphorylated at key activating residues, 2) is unable to transduce MAPK signaling towards MEK/ERK substrates, 3) is inherently unstable and prone to proteasome-mediated degradation and 4) is unable to block stress-induced apoptosis. On the basis of increased apoptosis detected in cellular systems and some morphological defects observed in the probands, the author classify this novel syndrome as a segmental progeroid syndrome.

Major comments:

The authors perform a thorough analysis of the structural and functional defects of the RAF1T543M protein, using in silico analyses and in vitro systems. The data are corroborated by an elegant and clear-cut experiment in Xenopus embryos that demonstrates that the mutated RAF is not able to transduce MAPK signaling when overexpressed in an in vivo model. The lack of patients' material prevents a validation in human cells, but I think the evidence collected in the manuscript is supportive of the loss-of-function mutation in RAF as the causative mutation of the observed phenotype.

The concept illustrated in the last sentence of the discussion, i.e. that different mutations in the same genes can either cause cancer (when overactivating) or premature aging (when blunting the activity of the enzyme) is fascinating. I think discussing this concept in the context of this manuscript is appropriate. However, the authors classify the syndrome caused by the RAF1T543M variant as a "novel segmental progeroid syndrome" in the title, abstract and first sentence of the discussion. I don't think that presented data are convincing for this classification. Indeed, two affected siblings die at very early post-natal stages (7 and 50 days, respectively)

likely because of malformations that are not compatible with life and that are due to altered in uthero development. Progeroid syndromes are a heterogeneous group of syndromes, a minority of which is characterized by malformations at birth. The more general concept is that physical abnormalities are progressively acquired during post-natal life, in specific tissues that show typical features associated with aging, including senescence, decline of the stem cell compartment, increased inflammation. In the case of individuals carrying the RAF1T543M variant, none of these tissue abnormalities have been reported (due to the lack of material from patients) and the classification as a progeroid syndrome is based on external inspection of organs. Reported phenotypes are not specific and a failure of in uthero development of heart, limbs and other organs seems like the absolutely predominant trait. This in uthero phenotype is perfectly consistent with the physiological role of the MAPK pathway downstream multiple RTKs that transduce morphogenetic, in addition to mitogenic, signals. As for the authors words: "endogenous FGF/FGFR signaling is required for proper mesoderm and neural induction" and supports the author claim that the analysis of individuals carrying the RAF1T543M variant "underscores the importance of RTK signaling during human development". I think that the "progeroid" phenotypes are less clear. It is still important to present the phenotypes reminiscent of progeroid syndromes and discuss them and perhaps more clearly explain the logical connection with the increased apoptosis observed in in vitro experiments.

In regard to the apoptosis experiment in figure 4, it supports the claim that RAF1 activity is necessary for protection from stress-induced apoptosis. However, the cartoon presented in figure 4C also shows that this process is mediated by increased ASK1/MST2 signaling. This part of the cartoon is based on the literature and has not been formally demonstrated. The authors can try to rescue the apoptotic status by silencing ASK1 in the RAF1T543M cells, similar to what has been done in Yamaguchi, O., 2004, Cardiac-specific disruption of the c-raf-1 gene induces cardiac dysfunction and apoptosis. J. Clin. Invest. 114, 937-943. Literature also suggests that RAF1-mediated inhibition of apoptosis is kinase-independent. This part is particularly interesting since the variant produces a protein that is both kinase-inactive and unstable. It would be a nice addition to the matuscript if the authors could clarify, at least in their cellular model, whether the increased apoptosis is due to the loss of either the protein or the kinase activity.

Minor comments:

A minor comment to the Xenopus ISH: the number of embryos that have been analyzed per condition is not reported anywhere. Figure legend explains that presented images are "representative pictures", but knowing the number of tested embryos and the penetrance of the phenotype would help undertsand the relevance of the RAF mutation in the signaling pathway under investigation.

2. Significance:

Significance (Required)

This work by Wong and colleagues provides conceptual advance and will be of interest for researchers dealing with RTK/MAPK signaling in multiple contexts including oncology, developmental biology and cardiomyopathy.

The gene under investigation is widely studied in cancer, where gain-of-function, oncogenic mutations are common. The role of RAF1 during embryonic development is less known. A couple of studies have investigated the developmental phenotype of mouse models carrying either a knock-out allele (Mikula, M, et al. Embryonic lethality and fetal liver apoptosis in mice lacking the c-raf-1 gene. EMBO J. 2001. 20:1952-1962) or an allele producing a truncated protein (Wojnowski L et al., (1998) Craf-1 protein kinase is essential for mouse development. Mech Dev, 76, 141-149). A few studies have investigated the conditional inactivation in specific tissues, such as the cardiac muscle (Yamaguchi, O., 2004, Cardiac-specific disruption of the c-raf-1 gene induces cardiac dysfunction and apoptosis. J. Clin. Invest. 114, 937-943.). And one study has found heterozygous carriers of a loss-of-function mutation among cohorts of children affected by dilated cardiomyopathy (Dhandapany, P.S., et al. (2014). RAF1 mutations in childhood-onset dilated cardiomyopathy. Nat. Genet. 46, 635-639). The manuscript reports the spectrum of defects acquired during embryonic development by carriers of a pathogenic mutation in the RAF1 gene. The mutation impairs both stability and kinase activity of the protein. The manuscript points out the non-redundant role of RAF1-mediated signaling in specific organs during embryonic development.

The person who is reviewing the manuscript has expertise in cellular signaling, mouse embryonic development and human aging.

3. How much time do you estimate the authors will need to complete the suggested revisions:

Estimated time to Complete Revisions (Required)

(Decision Recommendation)

Between 1 and 3 months

4. Do you want to get recognition for this review on a Web of Science researcher profile?

Don't let your reviewing work go unnoticed! Researchers the world over use Web of Science Reviewer Recognition to effortlessly track their valuable peer review contributions for any journal. If you opt in, your Web of Science profile will automatically be updated to show a verified record of this review in full compliance with the journal's review

policy. If you don't have a Web of Science profile, you will be prompted to create a free account.

Web of Science Reviewer Recognition

Yes

Full Revision

Review COMMONS

Manuscript number: RC -2022-01302
Corresponding author(s): Nathalie Escande-Beillard and Bruno Reversade

October 22nd 2022

Dear Editors and Reviewers,

We are grateful for your constructive comments and suggestions. As much as possible, we have attempted to address every question. For your easy reference, this is a list of all the notable changes we have implemented:

- A new Table 1 compares all 11 published ACFS cases with the two patients from this study.
- New panels C-D of Figure 4 reveal RAF1's control of ASK1 for stress-induced apoptosis.
- The term "progeroid" was removed from the title and is mentioned instead in the discussion. Our new title is" A neonatal lethal Syndrome Caused by RAF1 deficiency Underscores the importance of RTK signaling for Human Development".

Together with the revised manuscript we present herein our point-by-point rebuttal for your evaluation.

Bien a vous,

Bruno REVERSADE and Nathalie ESCANDE-BEILLARD

Full Revision

Review COMMONS

Manuscript number: RC -2022-01302
Corresponding author(s): Nathalie Escande-Beillard and Bruno Reversade

1. General Statements [optional]

We thank the reviewers for the comprehensive and insightful feedback on the manuscript. We have addressed each comment as comprehensively as possible, but would like to highlight the two major changes we implemented based on the reviewers' feedback on our initial submission.

First, we reassessed the use of the word "progeroid" to define the constellation of symptoms in our observed cases and agree with reviewers that the syndrome we observed should more accurately be described as a "neonatal lethal syndrome".

Second, we included additional data elucidating the mechanism behind how the T543M mutation affects apoptosis and how it relates to the phenotypic observations *in vitro* and in the described patients.

Additional details and explanations are found in the manuscript and in the point-by-point descriptions below.

2. Point-by-point description of the revisions

This section is mandatory. Please insert a point-by-point reply describing the revisions that were <u>already carried out and included</u> in the transferred manuscript.

Reviewer #1 (Evidence, reproducibility and clarity (Required)):

In the present manuscript, Wong et al describe for the first time the human phenotype resulting from the bi-allelic germline T543M mutation in the RAF1 proto-oncogene. Although somatic RAF1 mutations are outnumbered by BRAF mutations, they also occur in human cancer, but so far germline RAF1 mutations, usually dominant-acting ones abrogating RAF1 autoinhibition, have been mainly associated with RASopathies, in particular with Noonan syndrome. Here, Wong et al describe for the first time that the two individuals with a homozygous T543M mutation is associated with a neonatal lethal progeroid syndrome presenting with cutaneous, craniofacial, cardiac and limb
anomalies. Moreover, the affected residue, despite its conservation across RAF1 orthologues and in the ARAF and BRAF paralogues, has neither been described as a target of somatic and germ-line mutation in cancer and RASopathies, respectively.
The authors then use a combination of ectopic expression experiments in HEK293T cells and Xenopus embryos as well as CRISPR/Cas9 genome editing and structural bioinformatics to

provide several lines of evidence that RAF1 T543M represents a hypomorph suppressing ERK activation.
In summary, this is a very interesting and well-written manuscript with a stimulating discussion of the novel and convincing findings and the mechanisms driving the described pathology. The suggestions below might further strengthen this otherwise already very advanced manuscript.

Thank you for your expert and earnest review.

Major:
1. The data presented in this manuscript imply that RAF1 T543M represents a hypomorph suppressing ERK pathway activity. Nevertheless, it remains unclear whether this mutation reduces/abolishes the intrinsic activity of RAF1 or acts by a dominant-negative mechanism, e.g. by sequestrating critical components of RAF complexes, such as KSR proteins, or MEKs. To distinguish between both possibilities, it would be helpful, if the authors were able to document the intrinsic kinase activity of immunoprecipitated RAF1 T543M (and appropriate controls such as wildtype RAF1, the S257L gain-of-function allele and a truly kinase-dead variant, e.g. by mutating the aspartate of the DFG motif) towards recombinant and commercially available GST-MEK1.

It is accurate that we did not formally demonstrate the reduction of $RAF1^{T543M}$ enzymatic activity using an intrinsic kinase activity assay. We do not possess this capability. However, we believe that various independent experiments support the conclusion that T543M causes a reduction of its kinase activity. In particular, the following results support a hypomorphic rather than dominant-negative effect:

- Mutation is located in the kinase domain and is predicted to be deleterious using several independent *in silico* methods.
- Structural investigations (Fig. 2A to E) reveal that $RAF1^{T543M}$ abrogates RAF1 kinase activity potentially through disruption of a crucial local and possibly global structure within its kinase domain.
- Mutant $RAF1^{T543M}$ remained globally hypophosphorylated suggesting that it is refractory to EGF-triggered transduction (Fig.1E)(Dhillon et al. 2007).
- if T543M was endowed with dominant negative properties, its injection in *Xenopus* models would have shown reduced levels of FGF signaling and therefore no mesoderm induction. This was not seen in repeated experiments.
- In the same line of reasoning, if this allele was a dominant-negative variant, the parents who are heterozygous would be expected to have a phenotype, even if milder. However, they are entirely asymptomatic.

In addition, and in response to Reviewers 2 and 3's request, we have added in the last figure new experimental evidence showing a direct link between RAF1 LOF and ASK1 derepression. Particularly, we show that $RAF1^{T543M}$ is less efficient than $RAF1^{WT}$ to bind ASK1 (Fig. 4C) and

demonstrate an overall increase of the ASK1 pro-apoptotic signaling pathway in RAF1$^{T543M/T543M}$ cells compared to isogenic WT cells (Fig. 4D).

Interestingly, it has been shown that the mutation RAF1^{S338A} prevents RAF1/ASK1 complex formation (Alavi et al. 2007) because it prevents phosphorylation of Ser338 which itself is needed for efficient RAF1 and ERK activation. We too document reduced phosphorylation of Ser338 in RAF1^{T543M} (Fig. 1E) further supporting that this variant of RAF1 behaves as a LoF allele.

Albeit not a direct measurement as you requested, we are confident that the aforementioned results strongly lend credence to the notion that reduction of RAF1^{T543M} activity is rather due to intrinsic alterations in its kinase function rather than dominant-negative effects vis a vis binding partners.

2. RAF kinases engage in complex homo- and heterodimerization events. Does RAF1 T543M form homo- and heterodimers, e.g. with BRAF or ARAF, to a similar or different extent than wildtype RAF1?

Thank you for your suggestion. We have checked whether RAF1^{T543M} homodimerizes with RAF1^{WT} or heterodimerizes with BRAF. Unfortunately, the data are not always consistent but the overall trend seems to show that homodimerization to RAF1^{WT} and heterodimerization to BRAF are not really affected by RAF1^{T543M}. However homozygous T543M mutants cannot dimerise properly. We made the decision not to include these data in the revised manuscript as we cannot confidently conclude. The date is shown below for your evaluation (Fig. R1A-C) and will be seen along the reviews.

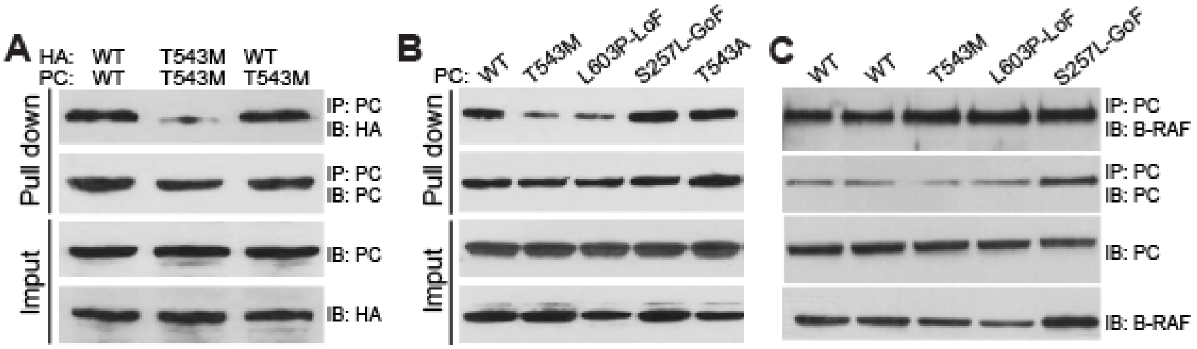

Fig. R1: (A) PC-tagged Immunoprecipitation showing that HA-RAF1 ^{WT} can dimerize with PC-RAF1 ^{WT} or PC-RAF1 ^{T543M} but PC-RAF1 ^{T543M} can not homodimerize. (B) Pull down showing that PC-RAF1 ^{T543M} and PC-RAF1 ^{L603P} can not dimerize with B-RAF while in a second pull down (C) they were able to bind B-RAF similarly to RAF1 ^{WT} or S257L-GoF.

Minor:
1. Abstract. To my knowledge, RAS but not RAF was the first human oncogene identified, albeit the discovery of RAF and its viral counterparts also took place very early in the oncogene discovery era.

Thank you for calling out this historical inaccuracy. It is true that RAS was discovered first in 1982 followed by c-RAF in 1984 (Malumbres and Barbacid 2003). To be more exact, we have updated the sentence in the abstract to “one of the first”.

2. Introduction, p. 2 "...while Braf-/- mice are embryonic lethal due to vascular defects.7" For a more balanced review, I would suggest citing additional and more recent papers describing the complex phenotypes of Braf deficient mice, e.g. PMIDs: 18332218 and PMID: 16432225

Thank you for the suggestion. We have expanded the mouse *Braf*$^{-/-}$ phenotype and added both citations in the introduction.

3. Figure 1D. Here, CRAF is used instead of RAF1. As the latter is the official gene/protein name RAF1 should be used to avoid confusion to readers outside of the RAF field.

You are right. We have corrected this mistake in Fig. 1D.

Reviewer #1 (Significance (Required)):

Very interesting novel findings to researchers being active in the signalling, cancer and developmental biology fields as well as to human geneticists and pediatricians.

My expertise: MAPK signalling, functional characterization of oncogenic mutations

Reviewer #2 (Evidence, reproducibility and clarity (Required)):

Summary:
The authors describe a homozygous missense variant in RAF1 identified in a child with a lethal malformation syndrome. The T543M variant not only blocks the kinase activity, but also destabilizes the RAF1 protein. In combination, this leads not only to a loss of MAPK signaling, but also to elevated apoptosis upon cell stress, which both together very likely explain the dramatic phenotype, which may correspond to the rarely described acro-cardio-facial syndrome.

Major comments:
The results of the functional analysis presented by the authors are highly convincing and clearly demonstrate a LOF effect of the identified variant. The experiments are described in sufficient detail and the statistical analysis is sound.

Thank you for your overall encouraging assessment.

Depending on the target journal the clinical information might be a bit scarce. There are neither clinical pictures nor molecular data from the first affected sibling. For a more clinically oriented journal, a summary of clinical features in form of a table and a comparison to ACFS will be a prerequisite and facilitates appreciation of this very special phenotype.

Thank you for your constructive suggestion. We have included an exhaustive clinical table (Table 1) that compares our cases to the 11 ACFS cases reported in the literature (Giannotti et al. 1995; Guion-Almeida, Zechi-Ceide, and Richieri-Costa 2000; Mingarelli et al. 2005; Kariminejad et al. 2008; Richieri-Costa 1987; Sivasli et al. 2007; Tanpaiboon et al. 2009; Hudson et al. 2014; Toschi et al. 2012). We hope this will provide more clinical context and can serve as a reference for the comparison of all reported ACFS cases.

The affected child seems much older than his chronological age, which seems due to the hypoplastic viscerocranium and the loss of subcutaneous fat tissue. The heart and limb defects have no link to chronological aging. Whether such phenotypes should be really called progeroid can be debated, but it is common practice (and always a good selling point). The authors do not discuss their findings in the light of chronological aging.

According to your observations and additional clinician input, we have decided not to emphasize the progeroid phenotype in the title and main part of the manuscript. Instead, we describe in the last part of the discussion how these clinical features may be reminiscent of a “progeroid” syndrome.

The hypothesis that the RAF1 LOF liberates ASK1 leading to increased apoptosis is very attractive. It would be very nice to show some experimental data proving this, but such data is not pivotal for the paper.

Thank you for your input. We agree that experimental evidence showing the direct link between RAF1 LOF and ASK1 would improve our manuscript.

Therefore, we have performed 2 additional assays to check whether (1) RAF1^{T543M} was less prone than RAF1^{WT} to bind ASK1 (Fig. 4C) and (2) ASK1 signaling was increased in RAF1$^{T543M/T543M}$ cells (Fig. 4D).

In the first set of experiments, we co-expressed ASK1 and PC-tagged RAF1^{WT} or RAF1^{T543M} in HEK293T cells and compared their binding by immunoprecipitation. PC-tagged RAF1^{T543M} pulled down ASK1 less efficiently than did PC-tagged RAF1^{WT}, suggesting that ASK1 may be disengaged in presence of RAF1^{T543M}. Moreover, the ratio of active pASK1 to total ASK1 was significantly increased when RAF1^{T543M} was overexpressed, confirming that unbound ASK1 in RAF1^{T543M} cells may be more prone to activation (Fig. 4C).

In the second set of experimentations, we compared, upon stress, endogenous ASK1 phosphorylation and downstream MAPKs apoptosis signaling pathway in our isogenic WT and RAF1 KI cells (Chen et al. 2001). Western blot shows a dose-dependent increase of activated pASK1 which was significantly greater in RAF1$^{T543M/T543M}$ cells. This was paralleled by an increase of c-Jun N-terminal kinase (JNK) phosphorylation and cleaved Caspase-3 (Fig. 4D). These new data are in line with those shown in Fig. 4A.

Taken together, these data confirm that stressed RAF1 mutant cells are more susceptible to apoptosis which we propose may be in part driven by ASK1 derepression (Fig. 4E).

Thank you for encouraging us to provide evidence for this phenomenon.
We hope this new data adds value and provides another line of interpretation for the pathogenesis of this disease.

Minor comments:

The facial and overall progeroid phenotype of the affected child is quite different from most of the described ACFS patients. In contrast, the prominent neurocranium with low hairline and the hypoplastic viscerocranium remind this reviewer of Gorlin-Chaudhry-Moss/Fontaine-Petty syndrome. This differential diagnosis is also interesting since the disorder is caused by GOF variants in a mitochondrial ATP transporter increasing the sensitivity for apoptosis. This underlines the suggested link between RAF1 and the apoptosis inducer ASK1.

There is probably no cellular function that has not been linked to the MAPK pathway. One of these connections is to cellular aging. Strongly activating variants in pathway members lead to oncogene-induced cellular senescence. Here, we have a progeroid phenotype, but a variant with LOF effect. This might be worthwhile to discuss.

The discussion could also use some sentences on the SHSF mechanism. FGFs produced by the AER are important for proliferation of the limb mesenchyme. What is the connection between MAPK and p63?

We thank the reviewers for highlighting these important points. For clarification, we have added the following paragraph in the discussion, which we are also showing here for your reference:

"Notably, the cutaneous, cardiac and limb anomalies of the two affected siblings we described above are similar to those documented in *Raf1* mutant animal models. Wojnowski and colleagues noted thinner and less differentiated dermal and epidermal layers in *Raf1*$^{-/-}$ mice[11], while Yamaguchi and colleagues found that heart-specific *Raf1*$^{-/-}$ mice exhibited cardiac dysfunction and apoptosis.[12] More recently, the recessive-acting *wingless2* mutation found in *Gallus gallus* – which results in a phenotype strikingly similar to this syndrome with truncated limbs, craniofacial, cardiac and skin/feather defects – was linked to a homozygous premature stop codon in *Raf1*.[42]

Additionally, these phenotypes have been observed in animal models containing mutations in other FGF signaling members. Mutations in mice of RAF1's downstream effector ERK2[43], and its upstream regulator p63[47–50], result in lethality with similar craniofacial, cardiovascular, and limb formation defects. These phenotypes were demonstrated to vary in severity dependent on global ERK activity.[43] Likewise, increasing severities of limb formation defects corresponded to decreasing levels of FGF signaling (ranging from loss of a single digit to entirely-truncated limbs) in mice with combinatorial knock-outs of Fgfr and Fgf gene family members in the apical ectodermal ridge (AER).[45,46] The SHFM characteristic of ACFS and our patient could similarly reflect a dampened FGF10-FGF8 positive feedback loop between the embryonic limb bud's mesoderm and its surrounding AER, and consequently reduced proliferation.[44] We note too that patients with mutations in components of the FGF pathway also present with highly-overlapping combinations of craniofacial, cardiovascular and limb phenotypes, collectively termed neuro-cardio-facial-cutaneous syndromes,[51] suggesting ACFS phenotypes could similarly be explained by defective FGF signaling."

Reviewer #2 (Significance (Required)):

This is a central signaling pathway and a prominent and historically outstanding oncogene. Up to now all variants in the MAPK pathway have been described as GOF and this is the first phenotype related to a LOF. Thus, this is a landmark paper widening the view on the pathway.
The paper is interesting for clinical and molecular geneticists, tumor biologists, and cell biologists.
This reviewer is a biochemist and clinical geneticist with research focus on mechanisms of skeletal and connective tissue disorders.

Reviewer #3 (Evidence, reproducibility and clarity (Required)):

The manuscript by Wong et al., provides the first report of a homozygous loss-of-function mutation of the RAF1 gene in humans. The mutation (T543M) was found in two siblings of a consanguineous family in association with perinatal death and multiple developmental abnormalities. These abnormalities show strong similarities with a rare congenital malformation syndrome with unknown aetiology named Acro-cardio-facial syndrome (ACFS, MIM600460). Conversely, reported abnormalities are different from those observed in RASopathies, congenital diseases caused by gain-of-function mutations in either the RAF gene or genes implicated in the same signaling pathway (MAPK) and that induce its ectopic/over-activation. By performing functional experiments in cellular systems (cell line where the mutated RAF was either overexpressed or knocked-in) and in Xenopus Laevis embryos, the authors demonstrate that the reason for the phenotypic differences compared to RASopathies is that the RAF1T543M variant impairs the signaling activity of RAF and blunts MAPK pathway activation. In particular, the RAF1T543M variant: 1) is not actively phosphorylated at key activating residues, 2) is unable to transduce MAPK signaling towards MEK/ERK substrates, 3) is inherently unstable and prone to proteasome-mediated degradation and 4) is unable to block stress-induced apoptosis. On the basis of increased apoptosis detected in cellular systems and some morphological defects observed in the probands, the author classify this novel syndrome as a segmental progeroid syndrome.

Major comments:
The authors perform a thorough analysis of the structural and functional defects of the RAF1T543M protein, using in silico analyses and in vitro systems. The data are corroborated by an elegant and clear-cut experiment in Xenopus embryos that demonstrates that the mutated RAF is not able to transduce MAPK signaling when overexpressed in an in vivo model. The lack of patients' material prevents a validation in human cells, but I think the evidence collected in the manuscript is supportive of the loss-of-function mutation in RAF as the causative mutation of the observed phenotype.

Thank you for your positive comments.

The concept illustrated in the last sentence of the discussion, i.e. that different mutations in the same genes can either cause cancer (when overactivating) or premature aging (when blunting the activity of the enzyme) is fascinating. I think discussing this concept in the context of this manuscript is appropriate. However, the authors classify the syndrome caused by the RAF1T543M variant as a "novel segmental progeroid syndrome" in the title, abstract and first sentence of the discussion. I don't think that presented data are convincing for this classification. Indeed, two affected siblings die at very early post-natal stages (7 and 50 days, respectively) likely because of malformations that are not compatible with life and that are due to altered in utero development. Progeroid syndromes are a heterogeneous group of syndromes, a minority of which is characterized by malformations at birth. The more general concept is that physical

abnormalities are progressively acquired during post-natal life, in specific tissues that show typical features associated with aging, including senescence, decline of the stem cell compartment, increased inflammation. In the case of individuals carrying the RAF1T543M variant, none of these tissue abnormalities have been reported (due to the lack of material from patients) and the classification as a progeroid syndrome is based on external inspection of organs. Reported phenotypes are not specific and a failure of in utero development of heart, limbs and other organs seems like the absolutely predominant trait. This in utero phenotype is perfectly consistent with the physiological role of the MAPK pathway downstream multiple RTKs that transduce morphogenetic, in addition to mitogenic, signals. As for the authors words: "endogenous FGF/FGFR signaling is required for proper mesoderm and neural induction" and supports the author claim that the analysis of individuals carrying the RAF1T543M variant "underscores the importance of RTK signaling during human development". I think that the "progeroid" phenotypes are less clear. It is still important to present the phenotypes reminiscent of progeroid syndromes and discuss them and perhaps more clearly explain the logical connection with the increased apoptosis observed in in vitro experiments.

Thank you for your comments. It has helped clarify the clinical features of our cases. Based on the points you raised and a secondary look at the literature, we agree that the term “progeroid” may not be the most appropriate description of the syndrome. Hence, we have decided not to emphasize the progeroid phenotype in the title, abstract and main part of the manuscript. Instead, we describe in the last part of the discussion how our reported cases resemble reported ACFS cases, but also emphasize that unlike other ACFS cases, we observed several clinical features reminiscent of a “progeroid” like syndrome.

Additionally, we included an exhaustive clinical table (Table 1) that compares our cases to the 11 ACFS cases reported in the literature (Richeri-Costa and Orquizas, 1987; Giannotti *et al*, 1995; Guion-Almeida *et al,* 2000; Mingarelli *et al,* 2005; Kariminejad *et al,* 2008; Sivasli *et al*, 2007; Tanpaiboon *et al,* 2009; Toschi *et al*, 2012; Hudson *et al*, 2014).

In regard to the apoptosis experiment in figure 4, it supports the claim that RAF1 activity is necessary for protection from stress-induced apoptosis. However, the cartoon presented in figure 4C also shows that this process is mediated by increased ASK1/MST2 signaling. This part of the cartoon is based on the literature and has not been formally demonstrated. The authors can try to rescue the apoptotic status by silencing ASK1 in the RAF1T543M cells, similar to what has been done in Yamaguchi, O., 2004, Cardiac-specific disruption of the c-raf-1 gene induces cardiac dysfunction and apoptosis. J. Clin. Invest. 114, 937-943. Literature also suggests that RAF1-mediated inhibition of apoptosis is kinase-independent. This part is particularly interesting since the variant produces a protein that is both kinase-inactive and unstable. It would be a nice addition to the manuscript if the authors could clarify, at least in their cellular model, whether the increased apoptosis is due to the loss of either the protein or the kinase activity.

Thank you for your suggestion and we agree that experimental evidence demonstrating a direct link between RAF1 LOF and ASK1 signaling would improve our manuscript. This was also requested by Reviewer 2.

To this end we have chosen to check whether (1) $RAF1^{T543M}$ was less efficient than $RAF1^{WT}$ to bind ASK1 (Fig. 4C) and (2) ASK1 signaling was increased in $RAF1^{T543M/T543M}$ cells (Fig. 4D).

In the first set of experiments, we co-expressed ASK1 and PC-tagged $RAF1^{WT}$ or $RAF1^{T543M}$ in HEK293T cells and compared their binding by immunoprecipitation. PC-tagged $RAF1^{T543M}$ pulled down ASK1 less efficiently than did PC-tagged $RAF1^{WT}$, suggesting that ASK1 may be disengaged in presence of $RAF1^{T543M}$. Moreover, the ratio of active pASK1 to total ASK1 was significantly increased when $RAF1^{T543M}$ was overexpressed, confirming that unbound ASK1 in $RAF1^{T543M}$ cells may be more prone to activation (Fig. 4C).

In the second set of experimentations, we examined, in isogenic WT and RAF1 KI cells that are subjected to stress, the levels of ASK1 phosphorylation and downstream MAPKs apoptosis signaling pathway (Chen et al, 2001). Indeed, we show by western blot a dose-dependent increase of activated pASK1 which was significantly greater in $RAF1^{T543M/T543M}$ cells. This was paralleled by an increase of c-Jun N-terminal kinase (JNK) phosphorylation and cleaved Caspase-3 (Fig. 4D). These new data are in line with those shown in Fig. 4A.

Taken together, these data confirm that stressed RAF1 mutant cells are more susceptible to apoptosis which we propose may be in part driven by ASK1 derepression (Figure 4E).

Thank you for prompting us to bring actual evidence, rather than just hypothesizing, a link between RAF1 and ASK1.

Minor comments:

A minor comment to the Xenopus ISH: the number of embryos that have been analyzed per condition is not reported anywhere. Figure legend explains that presented images are "representative pictures", but knowing the number of tested embryos and the penetrance of the phenotype would help understand the relevance of the RAF mutation in the signaling pathway under investigation.

Sorry for the oversight, all the numbers are now included in the corresponding legends (Figure 2).

Reviewer #3 (Significance (Required)):

This work by Wong and colleagues provides conceptual advance and will be of interest for researchers dealing with RTK/MAPK signaling in multiple contexts including oncology, developmental biology and cardiomyopathy.

The gene under investigation is widely studied in cancer, where gain-of-function, oncogenic mutations are common. The role of RAF1 during embryonic development is less known. A couple of studies have investigated the developmental phenotype of mouse models carrying either a knock-out allele (Mikula, M, et al. Embryonic lethality and fetal liver apoptosis in mice lacking the c-raf-1 gene. EMBO J. 2001. 20:1952-1962) or an allele producing a truncated protein (Wojnowski L et al., (1998) Craf-1 protein kinase is essential for mouse development. Mech Dev, 76, 141-149). A few studies have investigated the conditional inactivation in specific tissues, such as the cardiac muscle (Yamaguchi, O., 2004, Cardiac-specific disruption of the c-raf-1 gene induces cardiac dysfunction and apoptosis. J. Clin. Invest. 114, 937-943.). And one study has found heterozygous carriers of a loss-of-function mutation among cohorts of children affected by dilated cardiomyopathy (Dhandapany, P.S., et al. (2014). RAF1 mutations in childhood-onset dilated cardiomyopathy. Nat. Genet. 46, 635-639). The manuscript reports the spectrum of defects acquired during embryonic development by carriers of a pathogenic mutation in the RAF1 gene. The mutation impairs both stability and kinase activity of the protein. The manuscript points out the non-redundant role of RAF1-mediated signaling in specific organs during embryonic development.

The person who is reviewing the manuscript has expertise in cellular signaling, mouse embryonic development and human aging.

References:

Alavi, Alireza S., Lisette Acevedo, Wang Min, and David A. Cheresh. 2007. “Chemoresistance of Endothelial Cells Induced by Basic Fibroblast Growth Factor Depends on Raf-1–Mediated Inhibition of the Proapoptotic Kinase, ASK1.” *Cancer Research* 67 (6): 2766–72.

Chen, J., K. Fujii, L. Zhang, T. Roberts, and H. Fu. 2001. “Raf-1 Promotes Cell Survival by Antagonizing Apoptosis Signal-Regulating Kinase 1 through a MEK-ERK Independent Mechanism.” *Proceedings of the National Academy of Sciences*. https://doi.org/10.1073/pnas.141224398.

Dhillon, Amardeep S., Alex von Kriegsheim, Joan Grindlay, and Walter Kolch. 2007. “Phosphatase and Feedback Regulation of Raf-1 Signaling.” *Cell Cycle* 6 (1): 3–7.

Giannotti, A., M. C. Digilio, R. Mingarelli, and B. Dallapiccola. 1995. “An Autosomal Recessive Syndrome of Cleft Palate, Cardiac Defect, Genital Anomalies, and Ectrodactyly (CCGE).” *Journal of Medical Genetics* 32 (1): 72–74.

Guion-Almeida, M. L., R. M. Zechi-Ceide, and A. Richieri-Costa. 2000. “Cleft Lip/Palate, Abnormal Ears, Ectrodactyly, Congenital Heart Defect, and Growth Retardation: Definition of the Acro-Cardio-Facial Syndrome.” *Clinical Dysmorphology* 9 (4): 269–72.

Hudson, Cindy, Corbin Schwanke, John P. Johnson, Abdallah F. Elias, Sandy Phillips, Tammy Schwalbe, Mary Tunby, and Dongbin Xu. 2014. “Confirmation of 6q21-6q22.1 Deletion in Acro-Cardio-Facial Syndrome and Further Delineation of This Contiguous Gene Deletion Syndrome.” *American Journal of Medical Genetics. Part A* 164A (8): 2109–13.

Kariminejad, Ariana, Bita Bozorgmehr, Mohammad Ali Sedighi Gilani, Navid Almadani, and Mohamad Hasan Kariminejad. 2008. “Clinical Variability in Acro-Cardio-Facial-Syndrome.” *American Journal of Medical Genetics. Part A* 146A (15): 1977–79.

Malumbres, Marcos, and Mariano Barbacid. 2003. “RAS Oncogenes: The First 30 Years.” *Nature Reviews. Cancer* 3 (6): 459–65.

Mingarelli, Rita, Daniela Zuccarello, Maria Cristina Digilio, and Bruno Dallapiccola. 2005. “A New Observation of Acro-Cardio-Facial Syndrome Substantiates Interindividual Clinical Variability.” *American Journal of Medical Genetics. Part A* 136 (1): 84–86.

Richieri-Costa, A. 1987. “Tibial Hemimelia-Cleft Lip/Palate in a Brazilian Child Born to Consanguineous Parents.” *American Journal of Medical Genetics* 28 (2): 325–29.

Sivasli, O., E. A. Ozer, A. Ozer, H. Aydinlioglu, and M. Helvaci. 2007. “Acro-Cardio-Facial Syndrome Associated with Neuroepithelial Cyst: A Case Report.” *Genetic Counseling* 18 (2): 247–50.

Tanpaiboon, Pranoot, Rekwan Sittiwangkul, Prapai Dejkhamron, Metawee Srikummool, Warissara Sripathomsawat, and Piranit Kantaputra. 2009. “Expanding the Phenotypic Spectrum of Acro-Cardio-Facial Syndrome (ACFS): Exclusion of P63 Mutation.” *American Journal of Medical Genetics. Part A* 149 (8): 1749–53.

Toschi, Benedetta, Angelo Valetto, Veronica Bertini, Caterina Congregati, Massimiliano Cantinotti, Nadia Assanta, and Paolo Simi. 2012. “Acro-Cardio-Facial Syndrome: A Microdeletion Syndrome?” *American Journal of Medical Genetics. Part A* 158A (8): 1994–99.

18th Nov 2022

Dear Prof. Reversade,

Thank you for the submission of your manuscript to EMBO Molecular Medicine. We have now received the reports from the referees who re-reviewed your manuscript. As you will see below, they are now supportive of publication pending minor revisions, and I am therefore pleased to inform you that we will be able to accept your manuscript once the following points will be addressed:

1/ Referees comments:
- Please address all remaining concerns from referees #1 and #3 (including the discussion on the RAF1 T543M mutant mentioned by referee #1).

2/ Manuscript text:
- Please provide a .docx formatted version of the manuscript text (including legends for main figures, EV figures and tables). Please make sure that the changes are highlighted to be clearly visible.
- Please address the queries from our data editors in the Data edited MS file in track changes mode.
- We can accommodate a maximum of 5 keywords, please adjust accordingly.
- Remove "data not shown". As per our guidelines on "Unpublished Data", the journal does not permit citation of "Data not shown". All data referred to in the paper should be displayed in the main or Expanded View figures.
- Kindly remove the list of abbreviations, short title, and short summary from the manuscript file. Abbreviations should be defined the first time they appear in the text.
- Material and methods:
o This section should be placed after the discussion.
o Include a statement that informed consent was obtained from all subjects and that the experiments conformed to the principles set out in the WMA Declaration of Helsinki and the Department of Health and Human Services Belmont Report.
o Indicate the origin of the cells, and whether they were tested for mycoplasma contamination
o Please provide the antibody dilutions
o For animal work, confirm that all experiments were performed in accordance with relevant guidelines and regulations. The manuscript must include a statement in the Materials and Methods identifying the institutional and/or licensing committee approving the experiments.
- It is mandatory to include a 'Data Availability' section after the Materials and Methods. Before submitting your revision, primary datasets produced in this study need to be deposited in an appropriate public database, and the accession numbers and database listed under 'Data Availability'. Please remember to provide a reviewer password if the datasets are not yet public (see https://www.embopress.org/page/journal/17574684/authorguide#dataavailability). In case you have no data that requires deposition in a public database, please state so in this section (This study includes no data deposited in external repositories). Note that the Data Availability Section is restricted to new primary data that are part of this study.
- Acknowledgements: the complete funding information should be provided both in the acknowledgements and in the submission system.
- Author contributions: CRediT has replaced the traditional author contributions section because it offers a systematic machine-readable author contributions format that allows for more effective research assessment. Please remove the Authors Contributions from the manuscript and use the free text boxes beneath each contributing author's name in our system to add specific details on the author's contribution. More information is available in our guide to authors.
- Disclosure statement and competing interests: We updated our journal's competing interests policy in January 2022 and request authors to consider both actual and perceived competing interests. Please review the policy https://www.embopress.org/competing-interests and update your competing interests if necessary.
- Please reformat the references in alphabetical order, and with 10 authors listed before et al.

2/ Figures:
- Please provide exact p values (including for ns, non significant) in the figures or their legends.
- The Supplemental file should be renamed "Appendix", a table of content should be added, and the figures should be renamed "Appendix Figure S1/S2". Alternatively, you could choose to make these figures Expanded View (EV) Figures that are collapsible/expandable online. EV Figures should be cited as "Figure EV1, Figure EV2" in the text and their respective legends should be included in the main text after the legends of regular figures.

- Please add a title and legend to the file of Table 1.
- Please cover the eyes of the patient in Figure 1B with a black band.

3/ At EMBO Press we ask authors to provide source data for the main and EV figures.
Numerical data should be provided as individual .xls or .csv files (including a tab describing the data). For blots or microscopy, uncropped images should be submitted (using a zip archive if multiple images need to be supplied for one panel). Additional

information on source data and instruction on how to label the files are available at
.

4/ Please provide a complete author checklist, which you can download from our author guidelines (https://www.embopress.org/page/journal/17574684/authorguide#submissionofrevisions). Please insert information in the checklist that is also reflected in the manuscript. The completed author checklist will also be part of the RPF.

5/ We note that you currently have together with you a total of 3 co-corresponding authors. Is that correct? Do you confirm equal contribution of these 3 authors, able to take full responsibility for the paper and its content? While there is no limit per se to the number of co-corresponding authors, 3 is rare, and may not reflect as intended to the community.

6/ The paper explained: EMBO Molecular Medicine articles are accompanied by a summary of the articles to emphasize the major findings in the paper and their medical implications for the non-specialist reader. Please provide a draft summary of your article highlighting
- the medical issue you are addressing,
- the results obtained and
- their clinical impact.
This may be edited to ensure that readers understand the significance and context of the research. Please refer to any of our published articles for an example.

7/ Every published paper now includes a 'Synopsis' to further enhance discoverability. Synopses are displayed on the journal webpage and are freely accessible to all readers. They include a short stand first (maximum of 300 characters, including space) as well as 2-5 one-sentences bullet points that summarizes the paper. Please write the bullet points to summarize the key NEW findings. They should be designed to be complementary to the abstract - i.e. not repeat the same text. We encourage inclusion of key acronyms and quantitative information (maximum of 30 words / bullet point). Please use the passive voice. Please attach these in a separate file or send them by email, we will incorporate them accordingly.

Please also suggest a striking image or visual abstract to illustrate your article as a PNG file 550 px wide x 300-600 px high.

8/ As part of the EMBO Publications transparent editorial process initiative (see our Editorial at http://embomolmed.embopress.org/content/2/9/329), EMBO Molecular Medicine will publish online a Review Process File (RPF) to accompany accepted manuscripts.
This file will be published in conjunction with your paper and will include the anonymous referee reports, your point-by-point response and all pertinent correspondence relating to the manuscript. Let us know whether you agree with the publication of the RPF and as here, if you want to remove or not any figures from it prior to publication.
Please note that the Authors checklist will be published at the end of the RPF.

I look forward to receiving your revised manuscript.

Yours sincerely,

Lise Roth

Lise Roth, PhD
Senior Editor
EMBO Molecular Medicine

***** Reviewer's comments *****

Referee #1 (Remarks for Author):

As stated in my original assessment for Review Commons, this revised mansucript provides very interesting novel findings to

researchers being active in the signalling, cancer and developmental biology fields as well as to human geneticists and pediatricians. The authors have adequately responded to my comments, although the in vitro kinase assay experiment to confirm the absence of intrinsic kinase activity would have provided additional albeit not essential insights. The argumentation based on structural consideration represents strong evidence, but no definitive proof for the absence of kinase activity. Nevertheless, I welcome the authors' list of mostly convincing argumentation as to why the RAF1 T543M mutant is neither active nor acting in a dominant-negative manner. This could be included in the discussion at the editor's discretion.

I have only one comment that should be adressed prior to publication.

1. The authors often use the phosphorylation status of RAF1 as a surrogate marker for its activity or activation. It is correct that hyper-phosphorylation of RAF1, as reflected by its decreased electrophoretic mobility, indicates activation of the ERK pathway. However, it does not strictly correlate with RAF activity. There are multiple ERK mediated negative feedback phosphorylation sites contributing to this shift, which are rather involved in the downregulation of RAF1 activity (e.g. PMID: 15664191; PMID: 21613978). Of note, Dougherty et al. identified S289, S296, S301 as phosphosites associated with RAF1 inactivation PMID: 15664191. Therefore, the sentence on p.5/6. "Upon mitogenic stimulation, activating residues such as Ser338, Ser289, Ser296, Ser301 and Ser642 are phosphorylated while inhibitory residues like Ser259 are dephosphorylated" should be rephrased and I would also suggest to cite the original reports rather than or in addition to the review article (Ref. 13). Moreover, S259 is not an activating phosphorylation site. In fact seminal work by the Kolch (already cited in the revised manuscript), Rodriguez-Viciana and McCormick laboratories, as well as the very recent series of publications on the RAF/SHOC2 complex and the clustering of RASopathy mutations affecting the N-terminal 14-3-3 binding motif around S259 indicate that phosphorylation of this residue (and subsequent 14-3-3 binding) inhibit RAF1. Therefore, the sentence in the second paragraph of the discussion "...is not actively phosphorylated at key activating residues including Ser259.." should be rephrased. Dephosphorylation of S259 is necessary for RAF1 activation, while its (re)phosphorylation keeps (or brings) RAF1 (back) to the closed auto-inhibited conformation.

Referee #2 (Remarks for Author):

The authors have addressed all points raised by reviewers

Referee #3 (Comments on Novelty/Model System for Author):

The HEK293 model reliably shows the expected effects for the control RAF1 mutants. It is a widely used model system for studying genetic variants.

Referee #3 (Remarks for Author):

I thank the authors for answering my main queries and providing deeper insights into the increased stress sensitivity of the RAF1-T543M cell model.

Only some minor points remain to be addressed:

1. Please correct strange combination of lower and upper case letters in title.

2. It would strengthen the crucial message that a reduced binding of ASK1 to RAF1 leads to increased ASK1 phosphorylation and cellular stress sensitivity if the pulldown-efficiency for ASK1 represented by the blot in Fig. 4C was quantitatively and statistically evaluated.

3. Like in 3. the pASK1/ASK ratio in Fig. 4D should be quantified and evaluated statistically.

4. In the first sentence of the discussion it is said: "...etiology of a novel segmental neonatal syndrome with progeroid features...." This seems a misnomer. Moreover, I would not put the segmental progeroid features into the foreground. Better: "neonatal lethal malformation syndrome with progeroid features".

5." .. Thr543 which is mutated in this consanguineous family is unlikely to regulate RAF1 activity in a phosphorylation-dependent manner as substitutions with phosphorylation-inert residues have no effect on RAF1 activity. Incidentally, in zebrafish, the corresponding Tyr539 residue is a phenylalanine, ... " It takes several times reading until it gets clear that the authors want to state that phosphorylation not only of T543, but also of the opposing residue Y539 is unlikely to play a role. Please find clearer wording.

6. The discussion of the link between RAF1 LOF alleles and ACFS is slightly confusing. While the overlap between the patient described here and cases with ACFS is convincing this individual shows additional features not described for ACFS. I would therefore be more prudent and state that this "RASLOFopathy" belongs to the ACFS spectrum / is a progeroid variant of ACFS,

but not that it is ACSF per se like implied by this sentence: "In contrast to the more common gain- of-function RAF1 mutations seen in Noonan syndrome and other RASopathies, ACFS reflects instead on the physiological requirement of wild type RAF1,".

7. As often debated "progeroid features", which are surely present are not equivalent to "premature aging". A hypoplastic viscerocranium gives a progeroid impression, but is not a consequence of chronological aging. The only clear overlap between RAF1-related ACFS and aging I can see is the dermal and epidermal thinning.

***

Rev_Com_number: RC-2022-01302
New_manu_number: EMM-2022-17078
Corr_author: REVERSADE
Title: A neonatal lethal Syndrome Caused by RAF1 deficiency Underscores the importance of RTK signaling for Human Development

Point-by-point Response to Reviewers' Comments

EMM-2022-17078 | [RC-2022-01302] [REV]

Singapore, 21th December 2022

Dear Reviewers,

We are grateful for your constructive comments and suggestions. As much as possible, we have attempted to address every question carefully.

For your easy reference, the most notable changes incorporated in the revised manuscript are summarized below.

1- Fig. 4C: Addition of a graph showing quantification of pull down ASK1/RAF1 ratio.

2- New Expanded Fig. 3: Graph showing quantification of pASK1/ASK1 ratio.

3-Introduction: Rephrasing of RAF1 phosphorylation paragraph according to reviewer 1 suggestions.

3- Discussion: Rephrasing of several sentences according to reviewers 1 and 3 comments.

4- Title: Format of the letters have been unified and number of characters reduced.

Together with the revised manuscript we present herein our point-by-point responses (in blue) for your evaluation.

Best regards,

Bruno REVERSADE and Nathalie ESCANDE-BEILLARD

***** Reviewer's comments *****

Referee #1 (Remarks for Author):

As stated in my original assessment for Review Commons, this revised mansucript provides very interesting novel findings to researchers being active in the signalling, cancer and developmental biology fields as well as to human geneticists and pediatricians. The authors have adequately responded to my comments, although the in vitro kinase assay experiment to confirm the absence of intrinsic kinase activity would have provided additional albeit not essential insights. The argumentation based on structural consideration represents strong evidence, but no definitive proof for the absence of kinase activity.

Nevertheless, I welcome the authors' list of mostly convincing argumentation as to why the RAF1 T543M mutant is neither active nor acting in a dominant-negative manner. This could be included in the discussion at the editor's discretion.

I have **only one comment** that should be adressed prior to publication.

1. The authors often use the phosphorylation status of RAF1 as a surrogate marker for its activity or activation. It is correct that hyper-phosphorylation of RAF1, as reflected by its decreased electrophoretic mobility, indicates activation of the ERK pathway. However, it does not strictly correlate with RAF activity. There are multiple ERK mediated negative feedback phosphorylation sites contributing to this shift, which are rather involved in the downregulation of RAF1 activity (e.g. PMID: 15664191; PMID: 21613978). Of note, Dougherty et al. identified S289, S296, S301 as phosphosites associated with RAF1 inactivation PMID: 15664191. Therefore, **the sentence on p.5/6.** "Upon mitogenic stimulation, activating residues such as Ser338, Ser289, Ser296, Ser301 and Ser642 are phosphorylated while inhibitory residues like Ser259 are dephosphorylated" **should be rephrased** and I would also suggest to cite the original reports rather than or in addition to the review article (Ref. 13). Moreover, S259 is not an activating phosphorylation site. In fact seminal work by the Kolch (already cited in the revised manuscript), Rodriguez-Viciana and McCormick laboratories, as well as the very recent series of publications on the RAF/SHOC2 complex and the clustering of RASopathy mutations affecting the N-terminal 14-3-3 binding motif around S259 indicate that phosphorylation of this residue (and subsequent 14-3-3 binding) inhibit RAF1. **Therefore, the sentence in the second paragraph of the discussion** "...is not actively phosphorylated at key activating residues including Ser259.." **should be rephrased**. Dephosphorylation of S259 is necessary for RAF1 activation, while its (re)phosphorylation keeps (or brings) RAF1 (back) to the closed auto-inhibited conformation.

Thank you for pointing out this important distinction. We have rephrased the necessary sentences in the Introduction and the Discussion section to be more precise. We hope it captures the intricacy of RAF1 regulation by phosphorylation better.

Referee #2 (Remarks for Author):

The authors have addressed all points raised by reviewers

We are glad to have satisfactorily addressed your comments.

Referee #3 (Comments on Novelty/Model System for Author):

The HEK293 model reliably shows the expected effects for the control RAF1 mutants. It is a widely used model system for studying genetic variants.

Thank you for acknowledging our earnest efforts to improve and expand on our first submission.

Referee #3 (Remarks for Author):

I thank the authors for answering my main queries and providing deeper insights into the increased stress sensitivity of the RAF1-T543M cell model.

Only some minor points remain to be addressed:

1. Please correct strange combination of lower and upper case letters in title.

Thank you for pointing this out. We have corrected the title format according to your comments.

2. It would strengthen the crucial message that a reduced binding of ASK1 to RAF1 leads to increased ASK1 phosphorylation and cellular stress sensitivity if the pulldown-efficiency for ASK1 represented by the blot in Fig. 4C was quantitatively and statistically evaluated.

We agree and have performed the quantification and statistics in 3 independent experiments and have included the corresponding graph in the fig. 4C.

3. Like in 3. the pASK1/ASK ratio in Fig. 4D should be quantified and evaluated statistically.

Similarly, we have performed the quantification and statistics in 3 independent experiments and have included the corresponding graph in a new figure (Expanded fig.3).

4. In the first sentence of the discussion it is said: "...etiology of a novel segmental neonatal syndrome with progeroid features...." This seems a misnomer. Moreover, I would not put the segmental progeroid features into the foreground. Better: "neonatal lethal malformation syndrome with progeroid features".

Thank you for the suggestion. We accept this reviewer's suggestion as it is indeed a more precise description.

5." .. Thr543 which is mutated in this consanguineous family is unlikely to regulate RAF1 activity in a phosphorylation-dependent manner as substitutions with phosphorylation-inert residues have no effect on RAF1 activity. Incidentally, in zebrafish, the corresponding Tyr539 residue is a phenylalanine, ... " It takes several times reading until it gets clear that the authors want to state

that phosphorylation not only of T543, but also of the opposing residue Y539 is unlikely to play a role. **Please find clearer wording.**

Thank you for the suggestion. We have clarified the sentences by making them more concise.

6. The discussion of the link between **RAF1 LOF alleles and ACFS is slightly confusing**. While the overlap between the patient described here and cases with ACFS is convincing this individual shows additional features not described for ACFS. I would therefore be more prudent and state that this "RASLOFopathy" belongs to the ACFS spectrum / is a progeroid variant of ACFS, but not that it is ACSF per se like implied by this sentence: "In contrast to the more common gain- of-function RAF1 mutations seen in Noonan syndrome and other RASopathies, ACFS reflects instead on the physiological requirement of wild type RAF1,".

Thank you for the suggestion. We have clarified the distinction between our case, ACFS and RASopathies as a whole.

7. As often debated "progeroid features", which are surely present are not equivalent to "premature aging". A hypoplastic viscerocranium gives a progeroid impression, but is not a consequence of chronological aging. The only clear overlap between RAF1-related ACFS and aging I can see is the dermal and epidermal thinning.

Thank you for the suggestion. We have edited the text to clarify this. Additionally, we re-assessed the main text to strike a balance between avoiding giving the impression that our described syndrome is a definite premature aging syndrome, while still drawing attention to the additional progeroid features not common to ACFS.

<u>Point-by-point Response to Editors' Comments</u>

EMM-2022-17078 | [RC-2022-01302] [REV]

Singapore, 21th December 2022

1/ Referees comments:
- Please address all remaining concerns from referees #1 and #3 (including the discussion on the RAF1 T543M mutant mentioned by referee #1).

Please see " Point-by-point Response to Reviewers' Comments"

2/ Manuscript text:

- Please provide a .docx formatted version of the manuscript text (including legends for main figures, EV figures and tables). Please make sure that the changes are highlighted to be clearly visible.

Please see the revised manuscript uploaded. All the changes are highlighted in yellow in the manuscript

- Please address the queries from our data editors in the Data edited MS file in track changes mode.
- We can accommodate a maximum of 5 keywords, please adjust accordingly.

We have reduced to 5 keywords.

- Remove "data not shown". As per our guidelines on "Unpublished Data", the journal does not permit citation of "Data not shown". All data referred to in the paper should be displayed in the main or Expanded View figures.

Done

- Kindly remove the list of abbreviations, short title, and short summary from the manuscript file. Abbreviations should be defined the first time they appear in the text.

Done

- Material and methods:
o This section should be placed after the discussion.

Done
o Include a statement that informed consent was obtained from all subjects and that the experiments conformed to the principles set out in the WMA Declaration of Helsinki and the Department of Health and Human Services Belmont Report.

Done in “human subject” section

o Indicate the origin of the cells, and whether they were tested for mycoplasma contamination.

Done in “cell culture” section.

o Please provide the antibody dilutions

Done in “antibody” section. We also added the RRID for all of them.

o For animal work, confirm that all experiments were performed in accordance with relevant guidelines and regulations. The manuscript must include a statement in the Materials and Methods identifying the institutional and/or licensing committee approving the experiments.

Done in “*Xenopus* method” section.

- It is mandatory to include a 'Data Availability' section after the Materials and Methods. Before submitting your revision, primary datasets produced in this study need to be deposited in an appropriate public database, and the accession numbers and database listed under 'Data Availability'. Please remember to provide a reviewer password if the datasets are not yet public (see https://www.embopress.org/page/journal/17574684/authorguide#dataavailability). In case you have no data that requires deposition in a public database, please state so in this section (This study includes no data deposited in external repositories). Note that the Data Availability Section is restricted to new primary data that are part of this study.

Section added stating that “This study includes no data deposited in external repositories”.

- Acknowledgements: the complete funding information should be provided **both in the** acknowledgements and in the submission system.

Done

- Author contributions: CRediT has replaced the traditional author contributions section because it offers a systematic machine-readable author contributions format that allows for more effective research assessment. Please remove the Authors Contributions from the manuscript and use the free text boxes beneath each contributing author's name in our system to add specific details on the author's contribution. More information is available in our guide to authors.

Done

- Disclosure statement and competing interests: We updated our journal's competing interests policy in January 2022 and request authors to consider both actual and perceived competing interests. Please review the policy https://www.embopress.org/competing-interests and update your competing interests if necessary.

Section added at the end of the Material & Method section. No competing interests

- Please reformat the references in alphabetical order, and with 10 authors listed before et al.
Done according to the format requested

- Figures:
- Please provide exact p values (including for ns, non significant) in the figures or their legends.

Exact p values have been added in the figures.

- The Supplemental file should be renamed "Appendix", a table of content should be added, and the figures should be renamed "Appendix Figure S1/S2". Alternatively, you could choose to make these figures Expanded View (EV) Figures that are collapsible/expandable online. EV Figures should be cited as "Figure EV1, Figure EV2" in the text and their respective legends should be included in the main text after the legends of regular figures. https://www.embopress.org/page/journal/17574684/authorguide#expandedview

All the supplemental files have been renamed as "Expanded View". There are a total of 3 and they are cited as "Fig EVX" in the text.

- Please add a title and legend to the file of Table 1.

Done
- Please cover the eyes of the patient in Figure 1B with a black band.

Done

3/ At EMBO Press we ask authors to provide **source data for the main and EV figures**. Numerical data should be provided as individual .xls or .csv files (including a tab describing the data). For blots or microscopy, uncropped images should be submitted (using a zip archive if multiple images need to be supplied for one panel). Additional information on source data and instruction on how to label the files are available at https://www.embopress.org/page/journal/17574684/authorguide#sourcedata.

We prepared all the raw data and have submitted them in the online system.

4/ Please provide a **complete author checklist**, which you can download from our author guidelines (https://www.embopress.org/page/journal/17574684/authorguide#submissionofrevisions). Please insert information in the checklist that is also reflected in the manuscript. The completed author checklist will also be part of the RPF.

Done and submitted in the online system.

5/ We note that you currently have together with you a total of **3 co-corresponding authors**. Is that correct? Do you confirm equal contribution of these 3 authors, able to take full responsibility for the paper and its content? While there is no limit per se to the number of co-corresponding authors, 3 is rare, and may not reflect as intended to the community.

Yes we confirm that we are 3 co-corresponding authors.

6/ The paper explained: EMBO Molecular Medicine articles are accompanied by a summary of the articles to emphasize the major findings in the paper and their medical implications for the non-specialist reader. Please provide a draft summary of your article highlighting
- the medical issue you are addressing,
- the results obtained and
- their clinical impact.
This may be edited to ensure that readers understand the significance and context of the research. Please refer to any of our published articles for an example.

We added the "paper explained" section in the manuscript file after the "material & method" section.

7/ Every published paper now includes **a 'Synopsis'** to further enhance discoverability. Synopses are displayed on the journal webpage and are freely accessible to all readers. They include a short stand first (maximum of 300 characters, including space) as well as 2-5 one-sentences bullet points that summarizes the paper. Please write the bullet points to summarize the key NEW findings. They should be designed to be complementary to the abstract - i.e. not repeat the same text. We encourage inclusion of key acronyms and quantitative information (maximum of 30 words / bullet point). Please use the passive voice. Please attach these in a separate file or send them by email, we will incorporate them accordingly.

Please also suggest a striking image or visual abstract to illustrate your article as a PNG file 550 px wide x 300-600 px high.

Synopsis image and short stand first have been added in the online system.

8/ As part of the EMBO Publications transparent editorial process initiative (see our Editorial at http://embomolmed.embopress.org/content/2/9/329), EMBO Molecular Medicine will publish online a **Review Process File (RPF)** to accompany accepted manuscripts.
This file will be published in conjunction with your paper and will include the anonymous referee reports, your point-by-point response and all pertinent correspondence relating to the manuscript. Let us know whether you agree with the publication of the RPF and as here, if you want to remove or not any figures from it prior to publication.
Please note that the Authors checklist will be published at the end of the RPF.

We agree with the Review Process File.

Submission of Revisions

1. The manuscript text in LaTeX, RTF or MS Word format.
2. A letter with a detailed description of the changes made in response to the referees.
3. Three to four 'bullet points' highlighting the main findings of your study
4. A short 'blurb' text summarizing in two sentences the study (max. 250 characters). short 'blurb' text should be submitted as a separate manuscript file in LaTeX, RTF or MS Word format.

5. A "synopsis image", which can be used as a "visual title" for the synopsis section of your paper. The image should be PNG or JPG format with pixel dimensions of 550 x 300-600 (width x height).
6. *The latter three are for the paper's synopsis, which ensures the paper is more visible and browsable by a wide readership - this text is also used in condensed form on the electronic Table of Contents to complement the titles of papers.*

17th Jan 2023

Dear Prof. Reversade,

Thank you for the submission of your revised manuscript to EMBO Molecular Medicine and please accept my apologies for the delay in getting back to you during this busy time of the year.
Thank you for addressing the remaining concerns from the reviewers. I am therefore pleased to inform you that I will be able to accept your manuscript once the following editorial issues are addressed:

1/ Materials and Methods:
Please include a statement about randomization, blinding, and inclusion/exclusion criteria in the statistic section.

2/ Data Availability section:
Before submitting your revision, primary datasets produced in this study need to be deposited in an appropriate public database, and the accession numbers and database listed under 'Data Availability'.
Please deposit your whole-exome sequencing data in an appropriate repository. As it may take time before you get an accession number, and because the manuscript can only be published once you receive this number, I would suggest depositing your dataset as soon as possible.

3/ Checklist:
Please fill in the section about "Human research participants", as well as the section on statistics (blinding, randomization, exclusion criteria).

4/ I slightly edited your synopsis text to fit our size requirement, please let me know if you agree with the following:
"A novel recessive variant in RAF1 was identified as a possible cause of a hitherto unknown lethal neonatal syndrome with progeroid features. This private p.Thr543Met mutation abolished downstream ERK pathway signaling while increasing susceptibility to apoptosis via ASK1 de-repression.

- A homozygous p.Thr543Met mutation in RAF1 was found in a neonate presenting with a syndrome encompassing cutaneous, craniofacial, cardiac and limb anomalies.
- In cultured cells and in vivo using Xenopus assays the p.Thr543Met mutation behaved as a loss-of-function allele that abolishes downstream ERK pathway signaling in response to growth factor stimulation.
- p.Thr543Met knock-in in 293T cells revealed that RAF1T543M is unstable and more sensitive to stress-induced apoptosis via ASK1 de-repression.
- These findings describe, for the first time, the consequences of a loss of RAF1 function during human development, contrasting with gain-of-function mutations seen in cancer-prone RASopathies."

Thank you for providing a nice synopsis picture. Please upload it as a PNG/JPEG/TIFF file 550 px wide x 300-600 px high, and make sure that the text remains legible.

Once you have made the changes in the manuscript, please remove the highlighted text.

I look forward to receiving your revised manuscript.

Yours sincerely,

Lise Roth

Lise Roth, PhD

Senior Editor

EMBO Molecular Medicine

***
Rev_Com_number: RC-2022-01302
New_manu_number: EMM-2022-17078-V2
Corr_author: REVERSADE
Title: RAF1 deficiency causes a lethal syndrome that underscores RTK signaling during embryogenesis

The authors addressed the minor editorial issues.

15th Mar 2023

Dear Prof. Reversade,

Thank you for submitting your revised files, and for addressing as best as possible the sequencing data availability issue.

I am pleased to inform you that your manuscript is accepted for publication and is now being sent to our publisher to be included in the next available issue of EMBO Molecular Medicine.

Congratulations on your interesting work!

With kind regards,

Lise

Lise Roth, Ph.D
Senior Editor
EMBO Molecular Medicine

Follow us on Twitter @EmboMolMed
Sign up for eTOCs at embopress.org/alertsfeeds

*** *** *** IMPORTANT INFORMATION *** *** ***

SPEED OF PUBLICATION
The journal aims for rapid publication of papers, using using the advance online publication "Early View" to expedite the process: A properly copy-edited and formatted version will be published as "Early View" after the proofs have been corrected. Please help the Editors and publisher avoid delays by providing e-mail address(es), telephone and fax numbers at which author(s) can be contacted.

Should you be planning a Press Release on your article, please get in contact with embomolmed@wiley.com as early as possible, in order to coordinate publication and release dates.

LICENSE AND PAYMENT:

All articles published in EMBO Molecular Medicine are fully open access: immediately and freely available to read, download and share.

EMBO Molecular Medicine charges an article processing charge (APC) to cover the publication costs. You, as the corresponding author for this manuscript, should have already received a quote with the article processing fee separately. Please let us know in case this quote has not been received.

Once your article is at Wiley for editorial production you will receive an email from Wiley's Author Services system, which will ask you to log in and will present you with the publication license form for completion. Within the same system the publication fee can be paid by credit card, an invoice, pro forma invoice or purchase order can be requested.

Payment of the publication charge and the signed Open Access Agreement form must be received before the article can be published online.

PROOFS

You will receive the proofs by e-mail approximately 2 weeks after all relevant files have been sent o our Production Office. Please return them within 48 hours and if there should be any problems, please contact the production office at embopressproduction@wiley.com.

Please inform us if there is likely to be any difficulty in reaching you at the above address at that time. Failure to meet our deadlines may result in a delay of publication.

All further communications concerning your paper proofs should quote reference number EMM-2022-17078-V3 and be directed to the production office at embopressproduction@wiley.com.

***

Rev_Com_number: RC-2022-01302
New_manu_number: EMM-2022-17078-V3
Corr_author: REVERSADE
Title: RAF1 deficiency causes a lethal syndrome that underscores RTK signaling during embryogenesis

EMBO Press Author Checklist

Corresponding Author Name: Bruno Reversade, Nathalie Escande-Beillard	
Journal Submitted to: Embo Molecular Medicine	
Manuscript Number: EMM-2022-17078	

USEFUL LINKS FOR COMPLETING THIS FORM
The EMBO Journal - Author Guidelines
EMBO Reports - Author Guidelines
Molecular Systems Biology - Author Guidelines
EMBO Molecular Medicine - Author Guidelines

Reporting Checklist for Life Science Articles (updated January 2022)
This checklist is adapted from Materials Design Analysis Reporting (MDAR) Checklist for Authors. MDAR establishes a minimum set of requirements in transparent reporting in the life sciences (see Statement of Task: 10.31222/osf.io/9sm4x). Please follow the journal's guidelines in preparing your manuscript.
Please note that a copy of this checklist will be published alongside your article.

Abridged guidelines for figures
1. Data
The data shown in figures should satisfy the following conditions:
- the data were obtained and processed according to the field's best practice and are presented to reflect the results of the experiments in an accurate and unbiased manner.
- ideally, figure panels should include only measurements that are directly comparable to each other and obtained with the same assay.
- plots include clearly labeled error bars for independent experiments and sample sizes. Unless justified, error bars should not be shown for technical replicates.
- if n<5, the individual data points from each experiment should be plotted. Any statistical test employed should be justified.
- Source Data should be included to report the data underlying figures according to the guidelines set out in the authorship guidelines on Data Presentation.

2. Captions
Each figure caption should contain the following information, for each panel where they are relevant:
- a specification of the experimental system investigated (eg cell line, species name).
- the assay(s) and method(s) used to carry out the reported observations and measurements.
- an explicit mention of the biological and chemical entity(ies) that are being measured.
- an explicit mention of the biological and chemical entity(ies) that are altered/varied/perturbed in a controlled manner.
- the exact sample size (n) for each experimental group/condition, given as a number, not a range;
- a description of the sample collection allowing the reader to understand whether the samples represent technical or biological replicates (including how many animals, litters, cultures, etc.).
- a statement of how many times the experiment shown was independently replicated in the laboratory.
- definitions of statistical methods and measures:
  - common tests, such as t-test (please specify whether paired vs. unpaired), simple χ2 tests, Wilcoxon and Mann-Whitney tests, can be unambiguously identified by name only, but more complex techniques should be described in the methods section;
  - are tests one-sided or two-sided?
  - are there adjustments for multiple comparisons?
  - exact statistical test results, e.g., P values = x but not P values < x;
  - definition of 'center values' as median or average;
  - definition of error bars as s.d. or s.e.m.

Please complete ALL of the questions below.
Select "Not Applicable" only when the requested information is not relevant for your study.

Materials

Newly Created Materials	Information included in the manuscript?	In which section is the information available? (Reagents and Tools Table, Materials and Methods, Figures, Data Availability Section)
New materials and reagents need to be available; do any restrictions apply?	Yes	Materials and Methods

Antibodies	Information included in the manuscript?	In which section is the information available? (Reagents and Tools Table, Materials and Methods, Figures, Data Availability Section)
For **antibodies** provide the following information:<br>- Commercial antibodies: RRID (if possible) or supplier name, catalogue number and or/clone number<br>- Non-commercial: RRID or citation	Yes	Materials and Methods (Antibodies section)

DNA and RNA sequences	Information included in the manuscript?	In which section is the information available? (Reagents and Tools Table, Materials and Methods, Figures, Data Availability Section)
**Short novel DNA or RNA including primers, probes**: provide the sequences.	Yes	Materials and Methods

Cell materials	Information included in the manuscript?	In which section is the information available? (Reagents and Tools Table, Materials and Methods, Figures, Data Availability Section)
**Cell lines:** Provide species information, strain. Provide accession number in repository **OR** supplier name, catalog number, clone number, and/**OR** RRID.	Yes	Materials and Methods
**Primary cultures:** Provide species, strain, sex of origin, genetic modification status.	Not Applicable	
Report if the cell lines were recently **authenticated** (e.g., by STR profiling) and tested for mycoplasma contamination.	Yes	Materials and Methods

Experimental animals	Information included in the manuscript?	In which section is the information available? (Reagents and Tools Table, Materials and Methods, Figures, Data Availability Section)
**Laboratory animals or Model organisms:** Provide species, strain, sex, age, genetic modification status. Provide accession number in repository **OR** supplier name, catalog number, clone number, **OR** RRID.	Yes	Materials and Methods
**Animal observed in or captured from the field:** Provide species, sex, and age where possible.	Not Applicable	
Please detail **housing and husbandry conditions**.	Yes	Materials and Methods

Plants and microbes	Information included in the manuscript?	In which section is the information available? (Reagents and Tools Table, Materials and Methods, Figures, Data Availability Section)
**Plants:** provide species and strain, ecotype and cultivar where relevant, unique accession number if available, and source (including location for collected wild specimens).	Not Applicable	
**Microbes:** provide species and strain, unique accession number if available, and source.	Not Applicable	

Human research participants	Information included in the manuscript?	In which section is the information available? (Reagents and Tools Table, Materials and Methods, Figures, Data Availability Section)
If collected and within the bounds of privacy constraints report on age, sex and gender or ethnicity for all study participants.	Yes	Materials and Methods

Core facilities	Information included in the manuscript?	In which section is the information available? (Reagents and Tools Table, Materials and Methods, Figures, Data Availability Section)
If your work benefited from core facilities, was their service mentioned in the acknowledgments section?	Not Applicable	

Design

Study protocol	Information included in the manuscript?	In which section is the information available? (Reagents and Tools Table, Materials and Methods, Figures, Data Availability Section)
If study protocol has been **pre-registered, provide DOI in the manuscript**. For clinical trials, provide the trial registration number **OR** cite DOI.	Not Applicable	
Report the **clinical trial registration number** (at ClinicalTrials.gov or equivalent), where applicable.	Not Applicable	

Laboratory protocol	Information included in the manuscript?	In which section is the information available? (Reagents and Tools Table, Materials and Methods, Figures, Data Availability Section)
Provide DOI OR other citation details if **external detailed step-by-step protocols** are available.	Not Applicable	

Experimental study design and statistics	Information included in the manuscript?	In which section is the information available? (Reagents and Tools Table, Materials and Methods, Figures, Data Availability Section)
Include a statement about **sample size** estimate even if no statistical methods were used.	Yes	Materials and Methods and figure legends
Were any steps taken to minimize the effects of subjective bias when allocating animals/samples to treatment (e.g. **randomization procedure**)? If yes, have they been described?	Yes	Materials and Methods
Include a statement about **blinding** even if no blinding was done.	Yes	Materials and Methods
Describe **inclusion/exclusion criteria** if samples or animals were excluded from the analysis. Were the criteria pre-established?<br><br>If sample or data points were omitted from analysis, report if this was due to attrition or intentional exclusion and provide justification.	Yes	Materials and Methods
For every figure, are **statistical tests** justified as appropriate? Do the data meet the assumptions of the tests (e.g., normal distribution)? Describe any methods used to assess it. Is there an estimate of variation within each group of data? Is the variance similar between the groups that are being statistically compared?	Yes	Materials and Methods and figure legends

Sample definition and in-laboratory replication	Information included in the manuscript?	In which section is the information available? (Reagents and Tools Table, Materials and Methods, Figures, Data Availability Section)
In the figure legends: state number of times the experiment was **replicated** in laboratory.	Yes	Figure legends and Material and Methods
In the figure legends: define whether data describe **technical or biological replicates**.	Yes	Figure legends and Material and Methods

Ethics

Ethics	Information included in the manuscript?	In which section is the information available? (Reagents and Tools Table, Materials and Methods, Figures, Data Availability Section)
Studies involving **human participants**: State details of **authority granting ethics approval** (IRB or equivalent committee(s), provide reference number for approval.	Yes	Material and Methods
Studies involving **human participants**: Include a statement confirming that **informed consent** was obtained from all subjects and that the experiments conformed to the principles set out in the WMA Declaration of Helsinki and the Department of Health and Human Services Belmont Report.	Yes	Materials and Methods
Studies involving **human participants:** For publication of **patient photos**, include a statement confirming that consent to publish was obtained.	Yes	Materials and Methods
Studies involving experimental **animals**: State details of **authority granting ethics approval** (IRB or equivalent committee(s), provide reference number for approval. Include a statement of compliance with ethical regulations.	Yes	Materials and Methods
Studies involving **specimen and field samples:** State if relevant **permits** obtained, provide details of authority approving study; if none were required, explain why.	Not Applicable	

Dual Use Research of Concern (DURC)	Information included in the manuscript?	In which section is the information available? (Reagents and Tools Table, Materials and Methods, Figures, Data Availability Section)
Could your study fall under dual use research restrictions? Please check biosecurity documents and list of **select agents and toxins** (CDC): https://www.selectagents.gov/sat/list.htm	Not Applicable	
If you used a select agent, is the security level of the lab appropriate and reported in the manuscript?	Not Applicable	
If a study is subject to dual use research of concern regulations, is the name of the **authority granting approval and reference number** for the regulatory approval provided in the manuscript?	Not Applicable	

Reporting

The MDAR framework recommends adoption of discipline-specific guidelines, established and endorsed through community initiatives. Journals have their own policy about requiring specific guidelines and recommendations to complement MDAR.

Adherence to community standards	Information included in the manuscript?	In which section is the information available? (Reagents and Tools Table, Materials and Methods, Figures, Data Availability Section)
State if relevant guidelines or checklists (e.g., **ICMJE, MIBBI, ARRIVE, PRISMA**) have been followed or provided.	Not Applicable	
For **tumor marker prognostic studies,** we recommend that you follow the **REMARK** reporting guidelines (see link list at top right). See author guidelines, under 'Reporting Guidelines'. Please confirm you have followed these guidelines.	Not Applicable	
For **phase II and III randomized controlled trials**, please refer to the **CONSORT** flow diagram (see link list at top right) and submit the CONSORT checklist (see link list at top right) with your submission. See author guidelines, under 'Reporting Guidelines'. Please confirm you have submitted this list.	Not Applicable	

Data Availability

Data availability	Information included in the manuscript?	In which section is the information available? (Reagents and Tools Table, Materials and Methods, Figures, Data Availability Section)
Have **primary datasets** been deposited according to the journal's guidelines (see 'Data Deposition' section) and the respective accession numbers provided in the Data Availability Section?	Not Applicable	
Were **human clinical and genomic datasets** deposited in a public access-controlled repository in accordance to ethical obligations to the patients and to the applicable consent agreement?	Yes	Data Avaivility Section
Are **computational models** that are central and integral to a study available without restrictions in a machine-readable form? Were the relevant accession numbers or links provided?	Not Applicable	
If publicly available data were reused, provide the respective **data citations in the reference list**.	Not Applicable	
